# Asymptotic Posterior Normality of Multivariate Latent Traits in an IRT Model

**DOI:** 10.1007/s11336-021-09838-2

**Published:** 2022-02-11

**Authors:** Mia J. K. Kornely, Maria Kateri

**Affiliations:** Institute of Statistics, RWTH Aachen University, Aachen, Germany

**Keywords:** multidimensional item response theory, empirical Bayes, posterior distribution, ability estimation, consistency, normal approximation, Bernstein–von Mises theorem

## Abstract

The asymptotic posterior normality (APN) of the latent variable vector in an item response theory (IRT) model is a crucial argument in IRT modeling approaches. In case of a single latent trait and under general assumptions, Chang and Stout (Psychometrika, 58(1):37–52, 1993) proved the APN for a broad class of latent trait models for binary items. Under the same setup, they also showed the consistency of the latent trait’s maximum likelihood estimator (MLE). Since then, several modeling approaches have been developed that consider multivariate latent traits and assume their APN, a conjecture which has not been proved so far. We fill this theoretical gap by extending the results of Chang and Stout for multivariate latent traits. Further, we discuss the existence and consistency of MLEs, maximum a-posteriori and expected a-posteriori estimators for the latent traits under the same broad class of latent trait models.

**Supplementary Information:**

The online version contains supplementary material available at 10.1007/s11336-021-09838-2.

## Introduction

In the context of item response theory (IRT) methodology, statistical inference for the examinee’s ability relies often on the assumption that its posterior distribution given the test response is a normal distribution. As this is usually hard to justify and in contradiction to common models of the examinees abilities distribution in the population, it can assumed to be, for a long test, well approximated by a normal distribution. This assumption of asymptotic posterior normality (APN) is part of the famous Dutch identity conjecture of Holland (1990), who mentioned then that he was not aware of a thorough discussion of APN of latent variables and this would be an interesting area for future research. Shortly after, Chang and Stout (1993) proved the APN for univariate latent traits (LTs), mentioning that APN for multivariate LTs can be proved, but without providing further details or discussing the associated regularity conditions required.

As far as we know, APN of multivariate LTs has not been proved so far for IRT models of a general context, although posterior normality or APN is assumed quite often under various IRT setups (e.g., Anderson & Vermunt, 2000; Anderson & Yu, 2017; Anderson et al., 2007; Hessen, 2012; Li, 2010; Paek, 2016). For example, Pelle et al. (2016) assume posterior multivariate normality for the latent variable vector of a log-linear multidimensional Rasch model for capture–recapture analysis of registration data.

Sometimes the APN-assumption is justified by the APN in a Bayesian framework (pointing to the “Bernstein–von Mises Theorem”) without however proceeding to further details (e.g., the computationally efficient adaptive quadrature methods for high-dimensional item factor analysis (Schilling & Bock, 2005) and for generalized linear mixed models (Rabe-Hesketh et al., 2002) are based on the APN assumption).

In this work, we study the APN for multivariate latent trait models, focusing on models for dichotomous items and targeting at conditions that are tailored to IRT models and thus simpler to verify. APN of LTs, univariate or multivariate, is related to Bayesian asymptotics. In the light of this connection, we deepen in the approach of Ghosal et al. (1995), who discussed asymptotic posterior distributions in a very general setup that includes the regular cases and some non-regular cases as well. They also proved a general result on the asymptotic equivalence of the Bayes and maximum likelihood estimators, a well-known result for the regular cases. In particular, we generalize the approach and results of Chang and Stout (1993), CS hereafter, linking them to the semiproper centering concept of Ghosal et al. (1995), GGS hereafter, and embedding them in their approach. We provide conditions for multivariate APN that correspond one to one to the conditions of CS for univariate LTs, which is the standard approach for IRT models, as alternatives to the conditions imposed in Ghosal et al. (1995). Even for the case of univariate LTs, the proposed approach could be an interesting alternative to that of CS, since it has the advantage of applying also to models with non-monotone item response functions, which is not the case in the CS setup. Furthermore, we discuss conditions under which the existence of the maximum likelihood estimators (MLEs) for latent variable vectors is ensured. The consistency of MLEs under mild conditions, which was indicated as an open issue by Sinharay (2015), follows as a natural consequence of the proof of the APN. Finally, we prove the consistency of maximum a-posteriori (MAP) and expected a-posteriori (EAP) estimators for multivariate LTs.

The paper is organized as follows. Basic notation and the adopted IRT framework is set in Sect. 2, while the CS-theory for a univariate LT is briefly reviewed in Sect. 3. The approach of Ghosal et al. (1995) is discussed and linked to the APN of LTs and the CS-results in Sect. 4. The CS-conditions are generalized for the multivariate case and commented in Sect. 5 while they are verified for characteristic examples in Sect. 6. The main result on APN for multivariate LTs and properties of the MLEs, MAPs and EAPs of LTs are provided in Sect. 7 and supported by a simulation study in Sect. 8. Finally, the results are summarized in Sect. 9. A brief version of the proofs of the results of Sect. 7 is given in “Appendix” while their extended version can be found in the web-appendix. For a preliminary version of these results, see also Chapter 3 in Kornely (2021).

## Preliminaries

Consider a test consisting of *d* binary response variables $Y_i$, $i\in [d]:=\{1,\ldots ,d\}$, with $Y_i\in \{0,1\}$ for the *i*-th item, where 1 (0) denotes a correct (incorrect) response, and defined over a probability space $(\Omega ,\mathcal {A},\mathsf {P})$. Consider further the response vector ${\varvec{\mathsf {Y}}}^{(d)}=(Y_1, \ldots , Y_d)^{^{\intercal }}$, with superscript $^{^{\intercal }}$ denoting the transpose of a vector. Thus, the manifest probability for a specific response pattern ${\varvec{\mathsf {y}}}^{(d)}$ is given by $P({\varvec{\mathsf {y}}}^{(d)})=\mathsf {P}({\varvec{\mathsf {Y}}}^{(d)}= {\varvec{\mathsf {y}}}^{(d)})$. In an multidimensional IRT (MIRT) modeling framework, manifest probabilities are derived via conditioning on an absolutely continuous latent variable vector ${\varvec{\eta }}=(\eta _1,\ldots ,\eta _q)^{^{\intercal }}\in \Theta \subseteq \mathbb {R}^q$, defined over the same probability space as the binary items with probability density function (pdf) and cumulative distribution function (cdf) $\mathfrak {h}$ and $\mathcal {H}$, respectively. In particular, the conditional probability mass function (pmf) of $Y_i\,|\,\varvec{\eta }$ is thus given by

1 $$\begin{aligned} \mathsf {P}(Y_i=y\,|\,\varvec{\eta })=P_i(\varvec{\eta })^y(1-P_i(\varvec{\eta }))^{1-y},y\in \{0,1\}, \ \ i\in [d], \end{aligned}$$

with $P_i(\varvec{\eta })$ being known as the *i*-th item response function. In MIRT modeling, specific assumptions are usually imposed on the conditional distribution $\mathsf {P}({\varvec{\mathsf {Y}}}^{(d)}={\varvec{\mathsf {y}}}^{(d)}\,|\,\varvec{\eta })$; namely the assumption of *local independence*

2 $$\begin{aligned} P^{(d)}({\varvec{\mathsf {y}}}^{(d)}\,|\,\varvec{\eta }):=\mathsf {P}({\varvec{\mathsf {Y}}}^{(d)}={\varvec{\mathsf {y}}}^{(d)}\,|\,\varvec{\eta })&=\prod _{i=1}^d\mathsf {P}(Y_i=y_i\,|\,\varvec{\eta }),\quad {\varvec{\mathsf {y}}}^{(d)}\in \{0,1\}^d, \end{aligned}$$

and that of *monotonicity* for the item response functions $P_i(\varvec{\eta })$, i.e., for $i\in [d]$

3 $$\begin{aligned} P_i(\varvec{\eta }) \ \text {is strictly monotonic in every dimension of} \ \varvec{\eta }\ \text {being measured.} \end{aligned}$$

Note that assumption (3), which is required in the CS-approach, is relaxed in our setup. In the sequel, we denote by $\{Y_i\}_{i\in \mathbb {N}}\sim {\mathcal {P}}(\varvec{\eta })$ a sequence of Bernoulli random variables that fulfill (1) and (2) for all $d\in \mathbb {N}$.

Due to assumption (2) and using (1), the manifest probabilities are derived through the following integral

4 $$\begin{aligned} P({\varvec{\mathsf {y}}}^{(d)})=\int \ldots \int \Bigg (\prod _{i=1}^d P_i(\varvec{\eta })^{y_i}(1-P_i(\varvec{\eta }))^{1-y_i}\Bigg ) \mathfrak {h}(\varvec{\eta })\mathrm {d}\varvec{\eta }. \end{aligned}$$

### Remark 1

For simplicity of notation, we use $\varvec{\eta }$ to denote the random latent variable vector as well as a realization of it. If not clear from the context, we write explicitly $\varvec{\eta }\in \Theta$ for a realization or $\varvec{\eta }\sim \mathcal {H}$ for the random vector with values in $\Theta$. In the sequel, we abbreviate the term latent variable vector to latent vector.

The posterior density of $\varvec{\eta }$, given an observed response ${\varvec{\mathsf {y}}}^{(d)}\in \{0,1\}^d$, is then given by

5 $$\begin{aligned} h(\varvec{\eta }\,|\,{\varvec{\mathsf {y}}}^{(d)}):=\mathfrak {h}(\varvec{\eta }\,|\,{\varvec{\mathsf {Y}}}^{(d)}={\varvec{\mathsf {y}}}^{(d)})=\frac{\mathsf {P}({\varvec{\mathsf {Y}}}^{(d)}={\varvec{\mathsf {y}}}^{(d)}\,|\,\varvec{\eta })\mathfrak {h}(\varvec{\eta })}{\mathsf {P}({\varvec{\mathsf {Y}}}^{(d)}={\varvec{\mathsf {y}}}^{(d)})} =\frac{\exp (\ell ^{(d)}(\varvec{\eta }\,|\,{\varvec{\mathsf {y}}}^{(d)}))\mathfrak {h}(\varvec{\eta })}{P({\varvec{\mathsf {y}}}^{(d)})}, \end{aligned}$$

where $\ell ^{(d)}(\cdot \,|\,{\varvec{\mathsf {y}}}^{(d)}))$ is the log-likelihood corresponding to (1), given by

6 $$\begin{aligned} \ell ^{(d)}(\varvec{\eta }\,|\,{\varvec{\mathsf {y}}}^{(d)}))= \sum _{i=1}^d\left( y_i\lambda _i(\varvec{\eta })-\psi (\lambda _i(\varvec{\eta }))\right) , \end{aligned}$$

with $\lambda _i$ denoting the item logit, i.e.,

7 $$\begin{aligned} \lambda _i(\varvec{\eta }):=\log \left( \frac{P_i(\varvec{\eta })}{1-P_i(\varvec{\eta })}\right) , \end{aligned}$$

and the function $\psi (\cdot )$ being defined as $\psi (x)=\log \left( 1+\exp (x)\right)$, $x\in \mathbb {R}$.

Let ${\varvec{\hat{\eta }}}_d = {\varvec{\hat{\eta }}}({\varvec{\mathsf {y}}}^{(d)})$ denote the MLE of the true value of the latent vector $\varvec{\eta }_0$, based on a test realization ${\varvec{\mathsf {y}}}^{(d)}$. Furthermore, the Fisher information matrix of the test at point $\varvec{\eta }$ is given by

8 $$\begin{aligned} \mathcal {I}^{(d)}(\varvec{\eta }):=\mathsf {E}_{\varvec{\eta }}\left( \nabla \ell ^{(d)}(\varvec{\eta }\mid {\varvec{\mathsf {Y}}}^{(d)})\,\,\nabla ^{\intercal }\ell ^{(d)}(\varvec{\eta }\mid {\varvec{\mathsf {Y}}}^{(d)})\right) =\sum _{i=1}^d\mathcal {I}_i(\varvec{\eta }), \end{aligned}$$

where $\mathcal {I}_i(\cdot )$ is the *i*-th item information matrix

$$\begin{aligned} \mathcal {I}_i(\varvec{\eta }):=&\,\mathsf {E}_{\varvec{\eta }}\left( \nabla \log \Big (P_i(\varvec{\eta })^{Y_i}(1-P_i(\varvec{\eta }))^{1-Y_i}\Big )\,\,\nabla ^{\intercal }\log \Big (P_i(\varvec{\eta })^{Y_i}(1-P_i(\varvec{\eta }))^{1-Y_i}\Big ) \right) \\ =&P_i(\varvec{\eta })(1-P_i(\varvec{\eta }))\nabla \lambda _i(\varvec{\eta })\nabla \lambda _i(\varvec{\eta })^{\intercal },\quad \varvec{\eta }\in \Theta . \end{aligned}$$

This work studies the APN of $\varvec{\eta }$ for $d\rightarrow \infty$, based on a sequence of random variables $\{Y_i\}_{i\in \mathbb {N}}\sim {\mathcal {P}}(\varvec{\eta })$, as defined above. Particularly, we shall prove that, under certain conditions, (8) is invertible at ${\varvec{\hat{\eta }}}_d$ and $\varvec{\eta }\,|\,{\varvec{\mathsf {Y}}}^{(d)}={\varvec{\mathsf {y}}}^{(d)}$ is approximately normal distributed, ${\mathcal {N}}({\varvec{\hat{\eta }}}_d, [\mathcal {I}^{(d)}({\hat{\varvec{\eta }_d}})]^{-1})$, for a realization ${\varvec{\mathsf {y}}}^{(d)}$ of ${\varvec{\mathsf {Y}}}^{(d)}$. This enables the approximation of probabilities of the type

9 $$\begin{aligned} \mathsf {P}\left( \left. \mathcal {I}^{(d)}({\varvec{\hat{\eta }}}_d)^{1/2}\left( \varvec{\eta }-{\varvec{\hat{\eta }}}_d\right) \in B \,\right| \, {\varvec{\mathsf {Y}}}^{(d)}\right) , \ \ B\in \mathcal {B}^q, \end{aligned}$$

where $\mathcal {B}^q$ denotes the Borel-$\sigma$-algebra of $\mathbb {R}^q$. Practically speaking, a set *B* can be any countable union or intersection of *q*-dimensional real cubes.

Next, we define some functions that are useful for the sequel derivations. For all $d\in \mathbb {N}$, set $Z^{(d)}:\Theta \times \Theta \rightarrow \mathbb {R}$ with

10 $$\begin{aligned} Z^{(d)}(\varvec{\eta },\varvec{\eta }'):= \prod _{i=1}^d Z_i(\varvec{\eta },\varvec{\eta }'), \end{aligned}$$

where $Z_i:\Theta \times \Theta \rightarrow \mathbb {R}$, $i\in \mathbb {N}$, are defined as

11 $$\begin{aligned} Z_i(\varvec{\eta },\varvec{\eta }'):=\frac{P_i(\varvec{\eta })^{Y_i}(1-P_i(\varvec{\eta }))^{1-Y_i}}{P_i(\varvec{\eta }')^{Y_i}(1-P_i(\varvec{\eta }'))^{1-Y_i}} \ . \end{aligned}$$

Note that for given *d* and $\varvec{\eta },\,\varvec{\eta }'\in \Theta$, (10) is the likelihood ratio of the likelihoods for $\varvec{\eta }$ and $\varvec{\eta }'$. Furthermore,

12 $$\begin{aligned} \log \left( Z^{(d)}(\varvec{\eta },\varvec{\eta }')\right) =\sum _{i=1}^d\log Z_i(\varvec{\eta },\varvec{\eta }')=\ell ^{(d)}(\varvec{\eta }\mid {\varvec{\mathsf {Y}}}^{(d)})-\ell ^{(d)}(\varvec{\eta }'\mid {\varvec{\mathsf {Y}}}^{(d)}), \end{aligned}$$

while $-\mathsf {E}_{\varvec{\eta }_0}(\log Z_i(\varvec{\eta },\varvec{\eta }_0))$ is the Kullback–Leibler divergence between the conditional distributions of $Y_i$ given $\varvec{\eta }$ and $\varvec{\eta }_0$, respectively. A basic approach for deriving APN results relies on a quadratic approximation of (12).

## Review of APN for Univariate Latent Traits

In case of a single latent variable ($q=1$, $\varvec{\eta }=\eta$), Chang and Stout (1993) proved the APN of the univariate latent trait, adopting the approach of Walker (1969) for binary $Y_i$, $i\in [d]$, that are independent but not identically distributed (inid). We briefly review their results, so that we can extend in the sequel their approach to the multivariate case ($q>1$).

Additional to the general assumptions (2) and (3), they also introduced the following regularity conditions.

1.
  [i] Let $\eta \in \Theta$, where $\Theta \subseteq (-\infty ,\infty )$ is a bounded or unbounded interval.
  [ii] Let the prior density $\mathfrak {h}$ be continuous and positive at the true value $\eta _0$.
2. $P_i(\eta )$ is twice continuously differentiable with the first two derivatives being uniformly bounded in absolute value with respect to both $\eta$ and *i* in some closed interval $\Theta _0\subset \Theta$ around $\eta _0$.
3. For every fixed $\eta \ne \eta _0$, $\eta \in \Theta$, there is a $c(\eta )>0$ such that
  13 $$\begin{aligned} \limsup _{d\longrightarrow \infty }\frac{1}{d}\sum _{i=1}^d\mathsf {E}_{\eta _0}\log Z_i(\eta ,\eta _0)\le&-c(\eta ), \end{aligned}$$
  and $$\sup _{i\in \mathbb {N}}|\lambda _i(\eta )|<\infty $$.
4. If restricted to $\Theta _0$, the following sets of functions are uniformly bounded:
  $$\begin{aligned} \left\{ \left| \frac{\mathrm {d}\mathcal {I}_i}{\mathrm {d}\eta }\right| \mid i\in \mathbb {N}\right\} ,\quad \left\{ \left| \frac{\mathrm {d}^2 \lambda _i}{\mathrm {d}\eta ^2}\right| \mid i\in \mathbb {N}\right\} ,\quad \left\{ \left| \frac{\mathrm {d}^3 \lambda _i}{\mathrm {d}\eta ^3}\right| \mid i\in \mathbb {N}\right\} . \end{aligned}$$
5. Asymptotically, the average information at $\eta _0$ is bounded away from 0, i.e.,
  $$\begin{aligned} \liminf _{d\longrightarrow \infty }\frac{\mathcal {I}^{(d)}(\eta _0)}{d}>0. \end{aligned}$$

### Remark 2

With respect to the prior of $\eta$, additional to (CS1[ii]), Chang and Stout (1993) implicitly assumed its properness, which was stated explicitly in the earlier associated technical report (Chang & Stout, 1991, p. 15).

### Remark 3

Reasonable models for applications do not depend on a specific compact interval in $\Theta$ since usually $\eta _0$ is unknown. For this, also the conditions depending on $\eta _0$ should be satisfied for almost all $\eta _0\in \Theta$ and for almost each $\eta _0$ there should be some (arbitrary small) interval $\Theta _0$. In the usual models these conditions are satisfied.

Chang and Stout (1993) argued convincingly that conditions (CS1)–(CS5) are realistic and non-restrictive in practice for commonly used IRT models of well-designed tests. They particularly commented condition (CS3) and (13), which plays an important role in the proof of their main theorem. (CS3) is required when the item responses $\{Y_i\}_{i\in \mathbb {N}}$ are independent but not identically distributed. If they are iid, (CS3) is automatically satisfied, which however is not necessarily the case in IRT models. Their main results are expressed in the three theorems given below.

### Theorem 1

(Chang & Stout, 1993, Theorem 1) Suppose that conditions (CS1) through (CS5) hold for a fixed $\eta _0$. Let ${\hat{\eta }}_d$ be the MLE of $\eta _0$ and ${\hat{\sigma }}_d=(\mathcal {I}^{(d)}({\hat{\eta }}_d))^{-1/2}$. Then, for $-\infty \le a<b\le \infty$, the posterior probability of ${\hat{\eta }}_d+a{\hat{\sigma }}_d<\eta <{\hat{\eta }}_d+b{\hat{\sigma }}_d$ approaches the probability of $Z\in (a,b)$ in $\mathsf {P}_{\eta _0}$ for $Z\sim \mathcal {N}(0,1)$, that means

$$\begin{aligned} A_d \equiv \int _{{\hat{\eta }}_d+a{\hat{\sigma }}_d}^{{\hat{\eta }}_d+b{\hat{\sigma }}_d}h(\eta \mid {\varvec{\mathsf {Y}}}^{(d)})\,\mathrm {d}\eta \overset{\mathsf {P}_{\eta _0}}{\longrightarrow } \frac{1}{\sqrt{2\pi }}\int _a^b\exp \left( -\frac{1}{2}\eta ^2\right) \,\mathrm {d}\eta \equiv A,\quad \quad d\rightarrow \infty . \end{aligned}$$

### Theorem 2

(Chang & Stout, 1993, Theorem 2) Suppose that conditions (CS1) through (CS5) hold for fixed $\eta _0$ and let ${\hat{\eta }}_d$ and ${\hat{\sigma }}_d$ be defined as in Theorem 1. Then, for $-\infty \le a<b\le \infty$, the posterior probability $A_d$ approaches *A* $\mathsf {P}_{\eta _0}$-almost surely, as $d\rightarrow \infty$.

### Theorem 3

(Chang & Stout, 1993, Theorem 3) Assume $\Theta =\Theta _0$, a finite interval. Suppose that conditions (CS1) through (CS5) hold for all $\eta _0\in \Theta _0$ and let ${\hat{\eta }}_d$ and ${\hat{\sigma }}_d$ be defined as in Theorem 1. Then, for $-\infty \le a<b\le \infty$, the posterior probability $A_d$ approaches *A* in manifest probability $\mathsf {P}$, as $d\rightarrow \infty$.

The result of Theorem 3 does not depend on the true value $\eta _0$ and is thus of special practical interest for estimation and prediction purposes. As Chang and Stout (1993) comment, Theorems 1 and 3 treat sampling from a fixed ability sub-population and from the whole population, respectively. An important by-product of the proof of the APN of latent variables distributions was the establishment of the weak and strong consistency of the MLE of $\eta$ under milder conditions than Lord (1983).

Due to the theorems above, the following approximation for a large *d* and any observed response pattern ${\varvec{\mathsf {y}}}\in \{0,1\}^d$, i.e., the construction of asymptotic credible intervals, is justified

14 $$\begin{aligned} \mathsf {P}(a\le \eta \le b\mid {\varvec{\mathsf {Y}}}^{(d)}={\varvec{\mathsf {y}}})\approx \Phi _1(b\,;\,{\hat{\eta }}_d,{\hat{\sigma }}^2)-\Phi _1(a\,;\,{\hat{\eta }}_d,{\hat{\sigma }}^2), \end{aligned}$$

for $-\infty \le a\le b \le \infty \in \mathbb {R}$, where ${\hat{\eta }}_d$ is the MLE of $\eta _0$ based on the sample ${\varvec{\mathsf {y}}}$, ${\hat{\sigma }}^2=(\mathcal {I}^{(d)}({\hat{\eta }}_d))^{-1}$ and $\Phi _1(\cdot \,;\,{\hat{\eta }}_d,{\hat{\sigma }}^2)$ denotes the cdf of ${\mathcal {N}}({\hat{\eta }}_d,{\hat{\sigma }}^2)$. Approximation (14) is of special practical importance in the context of long tests where the exact computation of posterior probabilities for latent variables is commonly intractable. Furthermore, (14) allows the approximation of the posterior if the exact distribution $\mathcal {H}$ of $\eta$ is unavailable or uncertain.

Finally, Chang and Stout (1993) noted that their theory, under suitable regularity conditions, can be extended to prove the APN for latent vectors of general multidimensional IRT models, without however commenting further the proving procedure or the regularity conditions required. Next, we discuss the asymptotic posterior distribution of multivariate latent traits in the context of MIRT.

## APN for Multivariate Latent Traits

The theory of APN of the latent variables is naturally linked to Bayesian procedures and results on the convergence of posterior distributions. In particular, interesting and inspiring is the fundamental contribution by GGS (Ghosal et al., 1995), who consider asymptotic multivariate posterior distributions (not necessarily normal) in a very general and flexible framework discussing different types of convergence, relying on earlier works by Ghosh et al. (1994) and Ibragimov and Has’minskii (1981), denoted as IH hereafter. In particular, they studied posterior convergence of suitably centered and normalized posteriors. Their results provide a very general framework, which can be adopted for the APN in the IRT setup. Next, we adjust the GGS approach for MIRT models and discuss their conditions, embedding the CS approach in the GGS framework.

Following Ghosal et al. (1995, Definition 2), we distinguish two types of APN and link them to the statistic used for the centering of the posterior distribution of the latent vector.

### Definition 1

Let ${\varvec{\mathsf {Z}}}\sim \mathcal {N}_q({\varvec{\mathsf {0}}}, {\varvec{\mathsf {I}}}_q)$ be a *q*-variate standard normal distributed random vector. A $\mathbb {R}^q$-valued statistic ${\tilde{\varvec{\eta }}}_d$ is called a *proper centering (with limiting normal distribution)* if

15 $$\begin{aligned} \sup _{A\in \mathcal {B}^q}\left| \mathsf {P}\left( \mathcal {I}^{(d)}({\tilde{\varvec{\eta }}}_d)^{1/2}(\varvec{\eta }-{\tilde{\varvec{\eta }}}_d)\in A\left| \ {\varvec{\mathsf {Y}}}^{(d)}\right. \right) -\mathsf {P}({\varvec{\mathsf {Z}}}\in A)\right| \overset{\mathsf {P}_{\varvec{\eta }_0}}{\longrightarrow }0, \ \text {as} \ d\rightarrow \infty . \end{aligned}$$

A statistic ${\tilde{\varvec{\eta }}}_d$ is called *semiproper centering (with limiting normal distribution)* if, for all $A\in \mathcal {B}^q$,

16 $$\begin{aligned} \mathsf {P}\left( \mathcal {I}^{(d)}({\tilde{\varvec{\eta }}}_d)^{1/2}(\varvec{\eta }-{\tilde{\varvec{\eta }}}_d)\in A\left| \ {\varvec{\mathsf {Y}}}^{(d)}\right. \right) \overset{\mathsf {P}_{\varvec{\eta }_0}}{\longrightarrow }\mathsf {P}({\varvec{\mathsf {Z}}}\in A), \ \text {as} \ d\rightarrow \infty . \end{aligned}$$

A statistic ${\tilde{\varvec{\eta }}}_d$ is called *compatible* (with the posterior), if

$$\begin{aligned} \left( \mathcal {I}^{(d)}({\tilde{\varvec{\eta }}}_d)^{1/2}({\tilde{\varvec{\eta }}}_d-\varvec{\eta }_0),h^{*}\left( \,\cdot \,\left| \,{\varvec{\mathsf {Y}}}^{(d)}\right) \right. \right) , \end{aligned}$$

as a random element in $\mathbb {R}^q\times L^1(\mathbb {R}^q)$, converges in distribution for $d\rightarrow \infty$, where $h^{*}(\,\cdot \mid {\varvec{\mathsf {Y}}}^{(d)})$ denotes the density of the posterior distribution of $\mathcal {I}^{(d)}(\varvec{\eta }_0)^{1/2}(\varvec{\eta }-\varvec{\eta }_0)$ and $L^1(\mathbb {R}^q)$ stands for the space of all *q*-variate Lebesgue-integrable real functions on $\mathbb {R}^q$.

Proper and semiproper centering correspond to uniform and pointwise convergence of the posterior of the standardized latent vector $\mathcal {I}^{(d)}({\tilde{\varvec{\eta }}}_d)^{1/2}(\varvec{\eta }-{\tilde{\varvec{\eta }}}_d)$, respectively. Hence, proper centering is a stronger property than semiproper centering, and is consequently expected to require stronger assumptions.

Under this view, one can easily recognize that Theorem 1 of Chang and Stout (1993) is the semiproper centering of the MLE, since it can be formulated as

$$\begin{aligned} \mathsf {P}\left( \mathcal {I}^{(d)}({\hat{\eta }}_d)^{1/2}\left( \eta -{\hat{\eta }}_d\right) \in [a,b]\left| \ {\varvec{\mathsf {Y}}}^{(d)}\right. \right) \overset{\mathsf {P}_{\eta _0}}{\longrightarrow }\mathsf {P}\big (Z\in [a,b]\,\big ),\quad \quad d\longrightarrow \infty , \end{aligned}$$

for $Z\sim \mathcal {N}(0,1)$, $A=[a,b]\subset \mathbb {R}$ and ${{{\tilde{\eta }}}}_d={\hat{\eta }}_d$ being the MLE of $\eta _0$ based on ${\varvec{\mathsf {Y}}}^{(d)}$. Thus, for the extension of the CS-theory for multivariate LTs, we focus on semiproper centering.

The asymptotic results of GGS adjusted in our setup, primarily focus on the convergence of the posterior distribution of the standardized latent vector

17 $$\begin{aligned} \varvec{\eta }^* = \mathcal {I}^{(d)}({\tilde{\varvec{\eta }}}_d)^{1/2} (\varvec{\eta }-\varvec{\eta }_0), \end{aligned}$$

with $\varvec{\eta }^*\in \Theta _d:= \mathcal {I}^{(d)}({\tilde{\varvec{\eta }}}_d)^{1/2} (\Theta -\varvec{\eta }_0)$. We need the likelihood ratio (10) expressed in terms of $\varvec{\eta }^*$, which is denoted by

18 $$\begin{aligned} Z^{*(d)}(\varvec{\eta }^*):=Z^{(d)}(\varvec{\eta }_0+\mathcal {I}^{(d)}({\tilde{\varvec{\eta }}}_d)^{-1/2}\varvec{\eta }^*,\varvec{\eta }_0). \end{aligned}$$

In our setup, for binary response variables $Y_i$, $i\in [d]$, and log-likelihoods given by (6), the likelihood ratio $Z^{*(d)}$ takes the form

$$\begin{aligned} Z^{*(d)}(\varvec{\eta }^*)=&\exp \Bigg (\sum _{i=1}^d\Bigg (Y_i\left( \lambda _i\left( \varvec{\eta }_0+\mathcal {I}^{(d)}({\tilde{\varvec{\eta }}}_d)^{-1/2}\varvec{\eta }^*\right) -\lambda _i\Big (\varvec{\eta }_0\Big )\right) \\&-\left( \psi \left( \lambda _i\left( \varvec{\eta }_0+\mathcal {I}^{(d)}({\tilde{\varvec{\eta }}}_d)^{-1/2}\varvec{\eta }^*\right) \right) -\psi \left( \lambda _i\left( \varvec{\eta }_0\right) \right) \right) \Bigg ), \end{aligned}$$

with the item logits $\lambda _i$ provided in (7).

The primary conditions of GGS for APN are given as follows:

1. For some $M>0$, $m_1\ge 0$ and $\alpha >0$ holds
  $$\begin{aligned} \mathsf {E}_{\varvec{\eta }_0}\left( \left| Z^{*(d)}(\varvec{\eta }^*_1)^{1/2}-Z^{*(d)}(\varvec{\eta }^*_2)^{1/2}\right| ^2\right) \le M(1+R^{m_1})\Vert \varvec{\eta }_1^*-\varvec{\eta }_2^*\Vert ^\alpha , \end{aligned}$$
  for all $\varvec{\eta }_j^*\in \Theta _d$, satisfying $\Vert \varvec{\eta }_j^*\Vert \le R$, $j=1,2$, where $\Vert \cdot \Vert$ is the Euclidean norm.
2. For all $\varvec{\eta }^*\in \Theta _d$ holds
  $$\begin{aligned} \mathsf {E}_{\varvec{\eta }_0}\left( Z^{*(d)}(\varvec{\eta }^*)^{1/2}\right) \le \exp \Big (-g_d\big (\Vert \varvec{\eta }^*\Vert \big )\Big ), \end{aligned}$$
  where $\{g_d\}_{d\in \mathbb {N}}$ is a sequence of real-valued functions on $[0,\infty )$ satisfying the following: (a) for a fixed $d\ge 1$, $\lim _{x\rightarrow \infty }g_d(x)=\infty$; (b) for any $N>0$,
  $$\begin{aligned} \lim _{x\rightarrow \infty }\lim _{d\rightarrow \infty } x^N\exp (-g_d(x))=\lim _{d\rightarrow \infty }\lim _{x\rightarrow \infty } x^N\exp (-g_d(x))=0. \end{aligned}$$
3. For all $n\in \mathbb {N}$ and $\varvec{\eta }^*_1,\ldots ,\varvec{\eta }^*_n\in \mathbb {R}^q$, the vector of the likelihood-ratios, defined in (18), satisfies
  $$\begin{aligned} (Z^{*(d)}(\varvec{\eta }^*_1),\ldots ,Z^{*(d)}(\varvec{\eta }^*_n))\overset{\mathcal {D}}{\longrightarrow }(Z(\varvec{\eta }^*_1),\ldots ,Z(\varvec{\eta }^*_n)), \end{aligned}$$
  for $d\longrightarrow \infty$, where $\overset{\mathcal {D}}{\longrightarrow }$ denotes convergence in distribution and $Z(\varvec{\eta }^*)=\exp (\varvec{\xi }^T\varvec{\eta }^*-\frac{1}{2}\Vert \varvec{\eta }^*\Vert ^2)$, $\varvec{\eta }^*\in \mathbb {R}^q$, where $\varvec{\xi }\sim \mathcal {N}_q(\pmb 0,\mathrm {I}_q)$.

Under these conditions, Ghosal et al. (1995) provided the following general result. Notice that they discussed a far more general framework, allowing further distributions for the response variable and considering cases for which the posterior may converge to another distribution than a normal. We refer to GGS for further details regarding these cases.

### Theorem 4

(Ghosal et al., 1995, Theorem 1) Assume that conditions (GGS1) through (GGS3) hold. If either a proper centering or a semiproper compatible centering sequence $\{{\tilde{\varvec{\eta }}}_d\}_{d\in \mathbb {N}}$ exists, then it exists a random vector $\pmb W$, such that (a) $\mathcal {I}^{(d)}({\tilde{\varvec{\eta }}}_d)^{1/2}({\tilde{\varvec{\eta }}}_d-\varvec{\eta }_0)\overset{\mathcal {D}}{\longrightarrow }\pmb W$ for $d\longrightarrow \infty$ and (b) for almost all $\pmb x\in \mathbb {R}^q$, $\frac{Z(\pmb x-\pmb W)}{\int _{\mathbb {R}^q}Z(\pmb x^*-\pmb W)\,\mathrm {d}\pmb x^*}$ is nonrandom, where *Z* is as defined in condition (GGS3). Conversely, if (b) holds for a random vector $\pmb W$, then any Bayes estimator (with respect to a prior and loss considered by Ghosal et al. (1995)) is a compatible proper centering.

Applying Theorem 4 for an appropriate Bayes estimator for $\varvec{\eta }_0$, the APN of an MIRT model under conditions (GGS1) to (GGS3) is derived. The extension of Theorem 4 for an MLE, i.e., for ${\tilde{\varvec{\eta }}}_d = {\varvec{\hat{\eta }}}_d$, is based on its asymptotic equivalence to an arbitrary Bayes estimator, which has been proved by Ghosal et al. (1995, cf. Corollary 1) under (GGS2)–(GGS3) and the following strengthened form of (GGS1):

1. For some $M>0$, $m_1\ge 0$ and $m\ge \alpha >q$ holds
  $$\begin{aligned} \mathsf {E}_{\varvec{\eta }_0}\left( \left| Z^{*(d)}(\varvec{\eta }^*_1)^{1/m}-Z^{*(d)}(\varvec{\eta }^*_2)^{1/m}\right| ^m\right) \le M(1+R^{m_1})\Vert \varvec{\eta }_1^*-\varvec{\eta }_2^*\Vert ^\alpha , \end{aligned}$$
  for all $\varvec{\eta }_j^*\in \Theta _d$, satisfying $\Vert \varvec{\eta }_j^*\Vert \le R$, $j=1,2$.

### Remark 4

Alternatively to the GGS conditions discussed above, one could consider the conditions of Ibragimov & Has’minskii (1981, Section III.4) for general regular models for independent non-necessarily identical distributed (inid) random variables. They proved that these conditions are sufficient for the set of conditions N1–N4 of IH, Section III.1, where N1 is the *uniform asymptotic normality* and corresponds to (GGS3), while N3 and N4 correspond to (GGS1) and (GGS2), respectively.

## Regularity Conditions for Asymptotic Properties of Latent Vectors

Aiming to generalize the CS approach, we provide conditions for APN of (multivariate) LTs that correspond one to one to the conditions of CS for univariate LTs, which is the standard approach for IRT models, as alternatives to the conditions imposed in Ghosal et al. (1995). Throughout, we assume that $\{Y_i\}_{i\in \mathbb {N}}\sim {\mathcal {P}}(\varvec{\eta })$, i.e., $\{Y_i\}_{i\in \mathbb {N}}$ are Bernoulli random variables fulfilling (1) and (2), and that the true latent vector $\varvec{\eta }_0$ lies in the interior of the parameter space, i.e., $\varvec{\eta }_0\in \Theta \setminus \partial \Theta$, where $\partial \Theta$ denotes the boundary of $\Theta$. The asymptotic results of Sect. 7 rely on the following regularity conditions.

1.
  [i] The set $\Theta$ is closed, convex and has non-empty interior.
  [ii] The prior density $\mathfrak {h}$ of $\varvec{\eta }$ is proper and continuous at $\varvec{\eta }_0$ with $\mathfrak {h}(\varvec{\eta }_0)>0$.
2. $P_i$ is thrice continuously differentiable, $i\in \mathbb {N}$. If restricted to a compact subset $K\subseteq \Theta$, all $\left| \frac{\partial P_i}{\partial \eta _k} \right|$ and $\left| \frac{\partial ^2 P_i}{\partial \eta _k\partial \eta _j} \right|$ are uniformly bounded for all $i\in \mathbb {N}$, $1\le j,k\le q$. Moreover, there exist constants $0<\zeta _0(K)<\zeta _1(K)<1$, which are independent of $i\in \mathbb {N}$, such that
  19 $$\begin{aligned} \zeta _0(K)\le \inf _{(i,\varvec{\eta })\in \mathbb {N}\times K} P_i(\varvec{\eta })\le \sup _{(i,\varvec{\eta })\in \mathbb {N}\times K} P_i(\varvec{\eta })\le \zeta _1(K). \end{aligned}$$
3. For each $\varvec{\eta }\in \Theta$, $\varvec{\eta }\ne \varvec{\eta }_0$, there is a $c(\varvec{\eta })<0$ such that
  $$\begin{aligned} \limsup _{d\rightarrow \infty }\frac{1}{d}\sum _{i=1}^d\mathsf {E}_{\varvec{\eta }_0}(\log Z_i(\varvec{\eta },\varvec{\eta }_0))=\limsup _{d\rightarrow \infty }\frac{1}{d}\mathsf {E}_{\varvec{\eta }_0}(\ell ^{(d)}(\varvec{\eta }\,|\,{\varvec{\mathsf {Y}}}^{(d)})-\ell ^{(d)}(\varvec{\eta }_0\,|\,{\varvec{\mathsf {Y}}}^{(d)}))\le c(\varvec{\eta }), \end{aligned}$$
  and if $\Theta$ is unbounded holds additionally
  20 $$\begin{aligned} \sup _{\varvec{\eta }\in \Theta \setminus B_\delta (\varvec{\eta }_0)}c(\varvec{\eta })<0 , \quad \quad \text {for all }\delta >0 , \end{aligned}$$
  where $B_\delta (\varvec{\eta }_0):=\{\varvec{\eta }\in \mathbb {R}^q:\Vert \varvec{\eta }-\varvec{\eta }_0\Vert <\delta \}$ is the open ball of radius $\delta$ and center $\varvec{\eta }_0$.
4. If restricted to any compact set $K\subseteq \Theta$, the following set of functions is uniformly bounded
  $$\begin{aligned} \Bigg \{\left| \frac{\partial ^3 P_i}{\partial \eta _k\partial \eta _g\partial \eta _u}\right| \,&:\,i\in \mathbb {N},1\le k,g,u\le q \Bigg \}. \end{aligned}$$
5. For all $\varvec{\eta }\in \Theta$ holds
  21 $$\begin{aligned} \liminf _{d\rightarrow \infty }\,\nu _\text {min}\left( \frac{1}{d}\sum _{i=1}^d\nabla \lambda _i(\varvec{\eta })\nabla \lambda _i(\varvec{\eta })^{\intercal }\right) >0, \end{aligned}$$
  where $\nu _\text {min}$ denotes the smallest eigenvalue.

These regularity conditions correspond one to one to conditions (CS1)–(CS5), given in Sect. 3. For the comparison of these conditions, have in mind that convexity and connectivity are equivalent properties in $\mathbb {R}$. The convexity condition in (CS1’) is at first place stronger but it does not impose a real practical restriction, since non-convex $\Theta$ are only rarely needed in MIRT. Analogue to the CS-theory (s. Remark 3), conditions involving $\varvec{\eta }_0$, like $\mathfrak {h}(\varvec{\eta }_0)>0$, should be interpreted as $\mathfrak {h}>0$ almost surely. Condition (CS1’[ii]) on $\mathfrak {h}$ seems more strict than (CS1[ii]). However, Chang and Stout (1993) require additional a proper prior (s. Remark 2). Thus, under the consideration that $\varvec{\eta }_0$ is still unknown and we consider $\mathbb {R}^q$ instead of $\mathbb {R}$, the requirements on proper priors in (CS1’[ii]) are analogue to (CS1[ii]). Finally note that in the generalization of conditions (CS3) and (CS4), some requirements have been removed as the remaining requirements on $\lambda _i$, $i\in \mathbb {N}$, and its derivatives are implied by conditions (CS2’) and (CS4’).

A common assumption in one-dimensional IRT models ($q=1$) is the strict monotonicity assumption (3) of $P_i$ in $\eta$, for all $i\in \mathbb {N}$. Conceptually, this represents the notion that a more able subject has a higher probability of responding correct in any item of an educational test. Thus, models fulfilling this strict monotonicity assumption are easier to interpret. However, models with non-generalized-linear latent variable effects can be more adequate in practice. For example, Rizopoulos and Moustaki (2008) considered IRT models with possibly non-monotonic latent variable dependencies (like polynomial effects). Due to this reason, in order to allow for more flexible modeling options, in our semiproper centering theory, we abandon the requirement on strict monotonicity of $\varvec{\eta }\mapsto P_i(\varvec{\eta })$, for all $i\in \mathbb {N}$, in each component. Since the results of Chang and Stout (1993) rely on this monotonicity assumption, the merit of the current contribution is not only the extension of the CS-results for latent vectors ($q>1$) but also for univariate latent variables in case of a non-monotonic latent variable effect.

If all $P_i$, $i\in \mathbb {N}$, are strictly monotonic in each component, then requirement (19) of condition (CS2’) is satisfied as in the univariate case. Otherwise, the requirement in (19) is generally not really restrictive; it is the technical formulation of the notion that the response probabilities can (but not necessarily have to) approach zero or one only if $\Vert \varvec{\eta }\Vert \longrightarrow \infty$. Assumption (20) in (CS3’) serves for ensuring the identifiability of the latent vector in case of $\Vert \varvec{\eta }\Vert \longrightarrow \infty$. Hence, this condition is quite natural for a statistical model. In the univariate case, (20) of (CS3’) is implied by the strict monotonicity, too. But in contrast to (19), (20) cannot be concluded directly from the strict monotonicity of all $P_i$ in each component if $q>1$. Moreover, while a single item can suffice in the univariate case for identifiability, there are always at least *q* needed in the *q*-dimensional one. Similarly, the average test information $\frac{1}{d}\mathcal {I}^{(d)}(\varvec{\eta })$ is always singular for $d<q$, since it is a sum of *d* rank-one matrices. Condition (CS5’) ensures that $\frac{1}{d}\mathcal {I}^{(d)}(\varvec{\eta })$ becomes regular for $d\rightarrow \infty$ and can be interpreted as a condition to ensure that the asymptotic posterior of $\varvec{\eta }$ is regular *q*-dimensional distributed and does not have a lower dimensional support (cf. Lemma W.6 in the web-appendix).

To get a better impression of the conditions, we exemplary discuss them next for model (22), the multidimensional version of a model of Lee and Bolt (2018) and a logit model with an interaction of the latent variables (Rizopoulos, 2006).

## Verification of the CS Regularity Conditions for Multidimensional IRT Models

We shall verify the proposed conditions (CS1’) to (CS5’) for a multidimensional version of a model by Lee and Bolt (2018) and discuss them also for the models of Pelle et al. (2016) and a logit model with interaction of the latent variables (Rizopoulos, 2006).

Consider first the IRT model by Lee and Bolt (2018) or its multidimensional version

22 $$\begin{aligned} P_i(\varvec{\eta })=\Phi _1\left( \frac{\varvec{\alpha }_i^{^{\intercal }}\varvec{\eta }+\beta _i}{\sqrt{2}(1+ \exp (-{\varvec{\delta }}_i^{^{\intercal }}\varvec{\eta }))^{-1/2}}\right) ,\quad \quad \varvec{\eta }\in \Theta ,\,i\in \mathbb {N}. \end{aligned}$$

If $\mathcal {H}$ is one of the usual structural models or any other regular distribution, for example $\mathcal {N}_q({\varvec{\mathsf {0}}},{\varvec{\mathsf {I}}}_q)$, a mixture of normals or a uniform distribution on some compact set, then (CS1’) is directly satisfied. Further, considering the model parameters as random variables, we assume that the parameter sequences $\{\varvec{\alpha }_i\}_{i\in \mathbb {N}}$ and $\{\varvec{\delta }_i\}_{i\in \mathbb {N}}$ behave as they were two independent iid sequences drawn from absolutely continuous regular distributions in some bounded region in $\mathbb {R}^q$ and the sequence $\{\beta _i\}_{i\in \mathbb {N}}$ is in an arbitrary bounded subset of $\mathbb {R}$, then conditions (CS2’) and (CS4’) are directly satisfied. The assumption of regular distributions with a bounded support for the model parameters is reasonable, since in IRT practice items with arbitrarily large discrimination are not realistic and items of arbitrarily high or low difficulty are avoided. Furthermore, in almost all cases, the latent vector is identifiable if *q* arbitrary items are given. Hence, (CS3’) is satisfied, too. The gradient of the response probabilities is given by

23 $$\begin{aligned} \begin{aligned} \nabla P_i(\varvec{\eta })=\phi _1\bigg (&\frac{\pmb \alpha _i^{\intercal }\varvec{\eta }+\beta _i}{\sqrt{2}(1+\exp (-\pmb \delta _i^{\intercal }\varvec{\eta }))^{-1/2}}\bigg )\\&\times \Bigg (\sqrt{\frac{1+\exp (-\pmb \delta _i^{\intercal }\varvec{\eta })}{2}}\pmb \alpha _i-\frac{\exp (-\pmb \delta _i^{\intercal }\varvec{\eta })}{2\sqrt{2}}\frac{\pmb \alpha _i^{\intercal }\varvec{\eta }+\beta _i}{\sqrt{1+\exp (-\pmb \delta _i^{\intercal }\varvec{\eta })}}\pmb \delta _i\Bigg ), \end{aligned} \end{aligned}$$

for all $i\in \mathbb {N}$ and $\varvec{\eta }\in \Theta$, where $\phi _q$ denotes the pdf of $\mathcal {N}_q(\pmb 0,\mathrm {I}_q)$. In particular, we see from (23), that $\{\nabla P_i(\varvec{\eta })\}_{i\in \mathbb {N}}$ behaves in almost all cases for all $\varvec{\eta }\in \Theta$ as an iid sequence drawn from a regular distribution with bounded support in $\mathbb {R}^q$, since the parameters are iid distributed for all items and every $\varvec{\eta }\in \Theta$ is considered separately, i.e., $\varvec{\eta }$ is held fixed. Exceptions are pathological cases like the one in which zero belongs to the support of the distributions of all model parameters and all model parameters equal zero, i.e., $P_i(\varvec{\eta })=0.5$ for all $\varvec{\eta }\in \Theta$ and $i\in \mathbb {N}$. However, the subset of such cases is of zero probability for regular continuous distributions, i.e., is a null-set. Thus,

$$\begin{aligned} \frac{1}{d}\sum _{i=1}^d\nabla P_i(\varvec{\eta })\nabla P_i(\varvec{\eta })^{\intercal }\end{aligned}$$

converges to the second moment of the distribution of $\nabla P_1(\varvec{\eta })$, as a random vector formed by the multivariate transformation of the randomly selected parameter values described directly after equation (23), and is thus positive definite.

With respect to (CS5’), note that (19) in (CS2’) implies that (21) is equivalent to

24 $$\begin{aligned} \liminf _{d\rightarrow \infty }\,\nu _\text {min}\left( \frac{1}{d}\sum _{i=1}^d\mathcal {I}_i(\varvec{\eta })\right)>0\quad \text {and to}\quad \liminf _{d\rightarrow \infty }\,\nu _\text {min}\left( \frac{1}{d}\sum _{i=1}^d\nabla P_i(\varvec{\eta })\nabla P_i(\varvec{\eta })^{\intercal }\right) >0, \end{aligned}$$

which in our case ensures that (CS5’) is satisfied (cf. Lemma W.6 in the web-appendix).

For illustrative purposes, consider an example with $d=30$ and $q=2$ and model parameter values, as given in Table 1, which are independently drawn from a uniform distribution on $(-2,2)$ for $\beta _i$, and on $(-0.5,1)$ for all other parameters.

**Table 1:** Hypothetical parameter values for model (22) and the first 30 items.

*i*	$\beta _i$	$\alpha _{i1}$	$\alpha _{i2}$	$\delta _{i1}$	$\delta _{i2}$	*i*	$\beta _i$	$\alpha _{i1}$	$\alpha _{i2}$	$\delta _{i1}$	$\delta _{i2}$
1	$-$ 0.906	0.946	$-$ 0.398	0.230	0.327	16	$-$ 0.104	$-$ 0.498	0.298	0.359	0.622
2	$-$ 1.337	0.893	$-$ 0.085	0.696	0.622	17	0.458	0.496	0.267	0.677	0.551
3	$-$ 0.913	$-$ 0.363	0.703	0.052	0.605	18	$-$ 1.882	$-$ 0.259	$-$ 0.484	0.101	$-$ 0.022
4	0.387	0.166	0.313	$-$ 0.352	$-$ 0.447	19	0.801	0.066	$-$ 0.488	0.282	0.697
5	0.676	0.677	0.199	0.701	0.283	20	1.953	$-$ 0.169	0.963	$-$ 0.467	0.443
6	$-$ 0.084	0.865	0.833	0.957	0.724	21	$-$ 1.712	0.326	0.979	$-$ 0.146	$-$ 0.347
7	0.138	0.688	$-$ 0.444	0.942	$-$ 0.043	22	1.725	0.146	0.875	0.843	0.092
8	1.608	0.296	0.472	$-$ 0.096	$-$ 0.101	23	1.980	0.826	0.517	0.195	0.825
9	$-$ 0.882	0.016	$-$ 0.378	0.088	0.458	24	0.171	0.197	0.722	0.069	$-$ 0.442
10	1.240	0.320	0.924	$-$ 0.250	0.386	25	0.499	$-$ 0.101	$-$ 0.319	0.943	0.071
11	$-$ 0.919	0.867	$-$ 0.391	0.051	0.202	26	$-$ 1.775	0.530	$-$ 0.188	0.819	$-$ 0.336
12	0.216	0.093	0.028	0.152	0.339	27	1.523	$-$ 0.302	0.646	0.581	0.718
13	1.900	0.726	0.007	0.842	$-$ 0.072	28	1.548	0.272	0.469	$-$ 0.207	$-$ 0.241
14	0.510	$-$ 0.068	$-$ 0.105	0.132	$-$ 0.256	29	$-$ 0.823	$-$ 0.113	$-$ 0.147	0.027	$-$ 0.282
15	$-$ 1.109	0.072	$-$ 0.017	0.370	0.623	30	0.822	0.661	$-$ 0.485	$-$ 0.277	$-$ 0.017

**Fig. 1:**
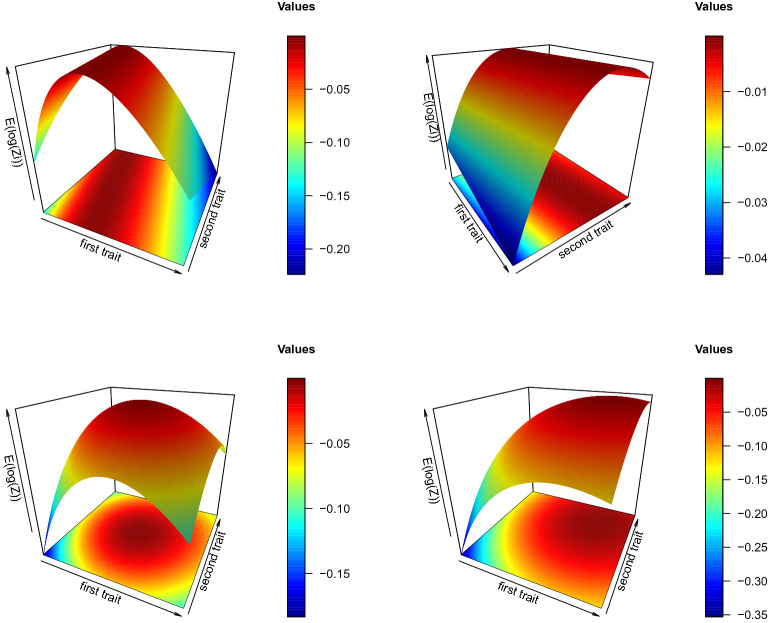
Top left:$\mathsf {E}_{(-0.5,0.1)}(\log (Z_5(\varvec{\eta },(-0.5,0.1))))$, top right: $\mathsf {E}_{(-0.1,0.3)}(\log (Z_9(\varvec{\eta },(-0.1,0.3))))$, bottom left: $\frac{1}{30}\sum _{i=1}^{30}\mathsf {E}_{(0,0)}(\log (Z_i(\varvec{\eta },(0,0))))$, bottom right: $\frac{1}{30}\sum _{i=1}^{30}\mathsf {E}_{(0.5,0.5)}(\log (Z_i(\varvec{\eta },(0.5,0.5))))$.

In Fig. 1 (top) visualizations of $\mathsf {E}_{\varvec{\eta }_0}(\log (Z_5(\varvec{\eta },\varvec{\eta }_0)))$ are provided for two exemplary values of $\varvec{\eta }_0\in \Theta$ and the parameters in Table 1. In particular, we can recognize lines in $\Theta$, for which $\mathsf {E}_{\varvec{\eta }_0}(\log (Z_5(\varvec{\eta },\varvec{\eta }_0)))=0$ holds. In Fig. 1 (bottom), surfaces of $\frac{1}{30}\sum _{i=1}^{30}\mathsf {E}_{\varvec{\eta }_0}(\log (Z_i(\varvec{\eta },\varvec{\eta }_0)))$ are illustrated for further two exemplary $\varvec{\eta }_0$ values. The surfaces are drawn over $[-1,1]^2$. There is a nearly parabolic surface, which illustrates that there is no reason to doubt for (20) in (CS3’).

**Fig. 2:**
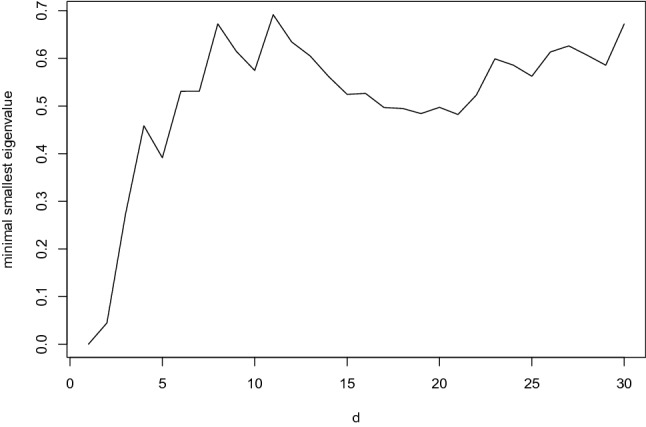
Minimal eigenvalue of $\frac{1}{d}\sum _{i=1}^d\nabla \lambda _i(\varvec{\eta })\nabla \lambda _i(\varvec{\eta })^{\intercal }$ for $\varvec{\eta }\in [-1,1]^2$.

Figure 2 provides the minimal smallest eigenvalue of $\frac{1}{d}\sum _{i=1}^d\nabla \lambda _i(\varvec{\eta })\nabla \lambda _i(\varvec{\eta })^{\intercal }$ on $[-1,1]^2$ for $d\in \{1,\ldots ,30\}$, cf. (CS5’). Overall, in this case, the regularity conditions can be considered as justified to apply the APN for arbitrary response patterns on the illustrated 30 items.

Conditions (CS1’) to (CS5’) for other models can be verified similarly. For example, the multidimensional Rasch model implemented in Pelle et al. (2016) is the logit model

25 $$\begin{aligned} \mathrm {logit}(\mathsf {P}(Y_i=1\mid \varvec{\eta })):=\log \left( \frac{\mathsf {P}(Y_i=1\mid \varvec{\eta })}{1-\mathsf {P}(Y_i=1\mid \varvec{\eta })}\right) =\alpha _{i0}+\pmb \alpha _i^{\intercal }\varvec{\eta },\quad \quad \varvec{\eta }\in \mathbb {R}^q,\quad i=1,\ldots ,d, \end{aligned}$$

where $\mathsf {P}(Y_i=1\mid \varvec{\eta })$ is the probability of inclusion in registration *i*, given the vector of latent variables. In this case,

$$\begin{aligned} \nabla \lambda _i(\varvec{\eta })=\pmb \alpha _i,\quad \quad i=1,\ldots ,d, \end{aligned}$$

would replace (23) for $\varvec{\eta }\in \Theta$, while the subsequent arguments are the same as above.

Another example is the two-dimensional model

$$\begin{aligned} \lambda _i(\varvec{\eta })=\alpha _{i0}+\alpha _{i1}\eta _1+\alpha _{i2}\eta _2+\alpha _{i3}\eta _1\eta _2,\quad \quad i=1,\ldots ,d, \end{aligned}$$

which is a logit model that contains an interaction term between the two latent variables and was considered by Rizopoulos (2006) (see Section 4). While it is still logit-linear in the model parameters $\alpha _{ij}$, $i\in [d]$, $j\in [4]$, it is no longer linear in the latent variables. However, with

$$\begin{aligned} \nabla \lambda _i(\varvec{\eta })=\begin{pmatrix} \alpha _{i1}+\alpha _{i3}\eta _2 \\ \alpha _{i2}+\alpha _{i3}\eta _1\end{pmatrix},\quad \quad \varvec{\eta }\in \mathbb {R}^2,\quad i=1,\ldots ,d, \end{aligned}$$

the same arguments still apply (compare also to Rizopoulos and Moustaki (2008), who discuss MIRT models within a more general form of the generalized latent variable model, allowing nonlinear effects of latent variables).

## Main Results

Our main contribution is the generalization of Theorems 1 and 3 of Chang and Stout (1993) for $q>1$, under the assumptions (CS1’) to (CS5’). Furthermore, we embed the CS-approach in the GGS framework (see Theorem 5 (iii)). Similarly to Chang and Stout (1993), the consistency of the MLE is received as a by-product, along with an assertion on its existence. Additionally, the consistency of a penalized MLE is derived. The results are provided in the next theorem, while their proofs along with some preliminary required lemmas are given in appendix.

### Theorem 5

Let ${\varvec{\mathsf {Z}}}\sim \mathcal {N}_q({\varvec{\mathsf {0}}}, {\varvec{\mathsf {I}}}_q)$ be a *q*-variate standard normal distributed random vector and $\{Y_i\}_{i\in \mathbb {N}}\sim {\mathcal {P}}(\varvec{\eta }_0)$ is a sequence of binary response variables for a sequence of item response functions $\{Y_i\}_{i\in \mathbb {N}}$ satisfying (CS1’[i]), (CS2’) and (CS3’) for $\varvec{\eta }_0\in \Theta \setminus \partial \Theta$. Then, the following statements holds:

(i) There is a sequence $\{{\varvec{\hat{\eta }}}_d\}_{d\in \mathbb {N}}$ of measurable mappings so that
  $$\begin{aligned}&\lim _{d\rightarrow \infty }\mathsf {P}_{\varvec{\eta }_0}\left( \nabla \ell ^{(d)}({\varvec{\hat{\eta }}}_d\,|\,{\varvec{\mathsf {Y}}}^{(d)})=\pmb 0\right) =1,\\&\lim _{d\rightarrow \infty }\mathsf {P}_{\varvec{\eta }_0}\left( \ell ^{(d)}({\varvec{\hat{\eta }}}_d\mid {\varvec{\mathsf {Y}}}^{(d)}) = \max _{\varvec{\eta }\in \Theta }\ell ^{(d)}(\varvec{\eta }\mid {\varvec{\mathsf {Y}}}^{(d)})\right) =1 \end{aligned}$$
  and $${\varvec{\hat{\eta }}}_d\overset{\mathsf {P}_{\varvec{\eta }_0}}{\longrightarrow }\varvec{\eta }_0$$ for $$d\rightarrow \infty $$.
(ii) Statement (i) remains valid if $\ell ^{(d)}$ is replaced by the penalized log-likelihood
  $$\begin{aligned} {\tilde{\ell }}^{(d)}(\varvec{\eta }\mid {\varvec{\mathsf {Y}}}^{(d)}))=\ell ^{(d)}(\varvec{\eta }\mid {\varvec{\mathsf {Y}}}^{(d)}) + \log (\mathcal {W}(\varvec{\eta })),\quad \quad \varvec{\eta }\in \Theta ,\, d\in \mathbb {N}, \end{aligned}$$
  for some continuously differentiable, positive and bounded function $\mathcal {W}$.

(iii) If additional (CS1’[ii]), (CS4’) and (CS5’) are satisfied, then the following statement holds: If $\varvec{\eta }_0$ is held fix, then, for all $B\in \mathcal {B}^q$,
  26 $$\begin{aligned} \mathsf {P}\left( \left. \mathcal {I}^{(d)}({\varvec{\hat{\eta }}}_d)^{1/2}\left( \varvec{\eta }-{\varvec{\hat{\eta }}}_d\right) \in B \,\right| \, {\varvec{\mathsf {Y}}}^{(d)}\right) \overset{\mathsf {P}_{\varvec{\eta }_0}}{\longrightarrow }\mathsf {P}({\varvec{\mathsf {Z}}}\in B). \end{aligned}$$
  That is, the MLE ${\varvec{\hat{\eta }}}_d$ is a semiproper centering (cf. Definition 1). If $\varvec{\eta }_0\sim \mathcal {G}$, where $\mathcal {G}$ is an absolutely continuous proper distribution with $\mathrm {supp}(\mathcal {G})\subseteq \Theta$, then furthermore
  27 $$\begin{aligned} \mathsf {P}\left( \left. \mathcal {I}^{(d)}({\varvec{\hat{\eta }}}_d)^{1/2}\left( \varvec{\eta }-{\varvec{\hat{\eta }}}_d\right) \in B \,\right| \, {\varvec{\mathsf {Y}}}^{(d)}\right) \overset{\mathsf {P}}{\longrightarrow }\mathsf {P}({\varvec{\mathsf {Z}}}\in B), \end{aligned}$$
  for all $$B\in \mathcal {B}^q$$.

### Remark 5

For $\mathcal {W}=\mathfrak {h}$, the penalized MLE in part Theorem 5 (ii) becomes the maximum a-posteriori estimator (MAP), which is an important estimator for $\varvec{\eta }$ in IRT, also because it ensures the existence of estimates in cases the MLE becomes infinite (for example when $\sum _{i=1}^d y_i=0$ or *d*). The restriction on $\mathfrak {h}$ in part (ii) is stronger than (CS1’[ii]), but still mild.

As already noted in Sect. 3, Theorem 5 (iii) can be used for the construction of credible regions for $\varvec{\eta }$. Additionally, it allows the interpretation of the MLE as a Bayesian estimator of $\varvec{\eta }_0$ and thus enables the use of ${\varvec{\hat{\eta }}}_d$ to derive some kind of *objective posterior*, in the sense that it is prior-free constructed.

An important concept in the asymptotic analysis of Bayesian procedures is the consistency of the posterior distribution, which forms a basis for the asymptotic validity of inferential methods, and is proved in Theorem 6 (i). The consistency of the EAP is stated in Theorem 6 (ii).

### Theorem 6

Consider the setup and the assumptions of Theorem 5(iii), the following statements hold:

(i) If $\varvec{\eta }_0$ is held fix, then
  $$\begin{aligned} \mathsf {P}(\varvec{\eta }\in B\mid {\varvec{\mathsf {Y}}}^{(d)})\overset{\mathsf {P}_{\varvec{\eta }_0}}{\longrightarrow }\delta _{\varvec{\eta }_0}(B):=\left\{ \begin{matrix} 1,&{}\varvec{\eta }_0\in B\\ 0,&{}\varvec{\eta }_0\not \in B \end{matrix}\right. ,\quad \quad d\rightarrow \infty , \end{aligned}$$
  for all Borel-sets $B\in \mathcal {B}^q$ with $\varvec{\eta }_0\not \in \partial B$.
(ii) Suppose that $\varvec{\eta }_0$ is held fix and that there is a continuous mapping $f:\Theta \rightarrow \mathbb {R}$ so that $\int _\Theta f(\varvec{\eta })\mathfrak {h}(\varvec{\eta })\,\mathrm {d}(\varvec{\eta })$ exists. Then, the posterior expected value $\mathsf {E}(f(\varvec{\eta })\mid {\varvec{\mathsf {Y}}}^{(d)})$ exists for all $d\in \mathbb {N}$ and is weakly consistent for $f(\varvec{\eta }_0)$, i.e.,
  $$\begin{aligned} \mathsf {E}(f(\varvec{\eta })\mid {\varvec{\mathsf {Y}}}^{(d)})\overset{\mathsf {P}_{\varvec{\eta }_0}}{\longrightarrow }f(\varvec{\eta }_0),\quad \quad \text { for }d\rightarrow \infty . \end{aligned}$$
  If in particular $\mathsf {E}(\varvec{\eta })$ exists, then the posterior expected value $\mathsf {E}(\varvec{\eta }\mid {\varvec{\mathsf {Y}}}^{(d)})$ exists for all $d\in \mathbb {N}$ and is weakly consistent for $\varvec{\eta }_0$.

## Simulation Study

The simulation study that follows examines the convergence to zero of the error for the approximation of the MLE-centered normalized posterior by a standard normal distribution and its relation to the convergence of the MLE, for the case of a bivariate latent variable vector ($q=2$). Convergences are evaluated based on the following measures. For the MLE, we use the root-mean-square error

$$\begin{aligned} \mathrm {RMSE}({\varvec{\hat{\eta }}}_d, \varvec{\eta }_0) = \sqrt{\frac{1}{2}\left( ({\hat{\eta }}_{d1}-\eta _{01})^2+({\hat{\eta }}_{d2}-\eta _{02})^2\right) }. \end{aligned}$$

For the approximation of the normalized posterior density $h^*$ by a bivariate normal pdf $\phi _2$, we compute the *density approximation error* (also known as $L^1$-distance)

$$\begin{aligned} \mathrm {DAE}(h^*, \phi _2)=\int _{\mathbb {R}^2}\left| h^*(\varvec{\eta })-\phi _2(\varvec{\eta })\right| \,\mathrm {d}\varvec{\eta }, \end{aligned}$$

the Hellinger-distance

$$\begin{aligned} \mathrm {HD}(h^*, \phi _2)=\sqrt{\frac{1}{2}\int _{\mathbb {R}^2}\left( \sqrt{h^*(\varvec{\eta })}-\sqrt{\phi _2(\varvec{\eta })}\right) ^2\,\mathrm {d}\varvec{\eta }} , \end{aligned}$$

and the Kullback–Leibler divergence

$$\begin{aligned} \mathrm {KLD}(h^*, \phi _2)=\int _{\mathbb {R}^2}\log \left( \frac{\phi _2(\varvec{\eta })}{h^*(\varvec{\eta })}\right) \phi _2(\varvec{\eta })\,\mathrm {d}\varvec{\eta }. \end{aligned}$$

The simulation study is based on model (22) with the same item parameters across all replications, to mimic the situation that different persons respond on the same test. These are generated as in Sect. 6. For the structural model we assume $\mathcal {H}=\mathcal {N}_2(\pmb 0,\mathrm {I}_2)$, resulting in $\Theta =\mathbb {R}^2$. The number of items *d* varies from 10 to 70 in steps of ten items, to mimic the asymptotic behavior with *test lengthening*. All involved integrals are approximated using an importance sampling Monte Carlo (MC) approximation with $\mathcal {N}_2(\pmb 0,\mathrm {I}_2)$ being the importance distribution.

We replicate 1000 times ($\ell =1,\ldots , 1000$) the following procedure.

1. Draw $$\varvec{\eta }_0^{(\ell )}\sim \mathcal {H}$$.
2. Draw ${\varvec{\mathsf {y}}}^{(70, \ell )}=(y_1^{(\ell )},\ldots ,y_{70}^{(\ell )})$ from model (22) with underlying true latent variable vector $\varvec{\eta }_0^{(\ell )}$ and item parameter values as described above (setting $y_i^{(\ell )}=1$ if $y_i^{*(\ell )}<P_i(\varvec{\eta }_0^{(\ell )})$ and $y_i=0$ otherwise, where $y_i^{*(\ell )}$ is drawn from iid $\mathcal {U}(0,1)$, $i=1,\ldots , 70$). Then set ${\varvec{\mathsf {y}}}^{(d,\ell )}=(y_1^{(\ell )},\ldots ,y_{d}^{(\ell )})$, for $d=10, 20, \ldots , 70$.
3. Compute the MLE ${\varvec{\hat{\eta }}}_d^{(\ell )}$ and the test information matrix $\mathcal {I}^{(d)}({\varvec{\hat{\eta }}}_d^{(\ell )})$, based on ${\varvec{\mathsf {y}}}^{(d,\ell )}$, for $d=10, 20, \ldots , 70$.
4. Derive the posterior pdf $h^{*(\ell )}$ of the normalized latent vector $\mathcal {I}^{(d)}({\varvec{\hat{\eta }}}_d)\big (\varvec{\eta }-{\varvec{\hat{\eta }}}_d\big )$, estimating its normalization constant by a MC quadrature.
5. Compute $\mathrm {RMSE}_{\ell }^{(d)} = \mathrm {RMSE}({\varvec{\hat{\eta }}}_d^{(\ell )}, \varvec{\eta }_0^{(\ell )})$, $\mathrm {DAE}_{\ell }^{(d)} = \mathrm {DAE}(h^{*(\ell )}, \phi _2)$, $\mathrm {HD}_{\ell }^{(d)} = \mathrm {HD}(h^{*(\ell )}, \phi _2)$ and $\mathrm {KLD}_{\ell }^{(d)} = \mathrm {KLD}(h^{*(\ell )}, \phi _2)$, for $d=10, 20, \ldots , 70$.

**Fig. 3:**
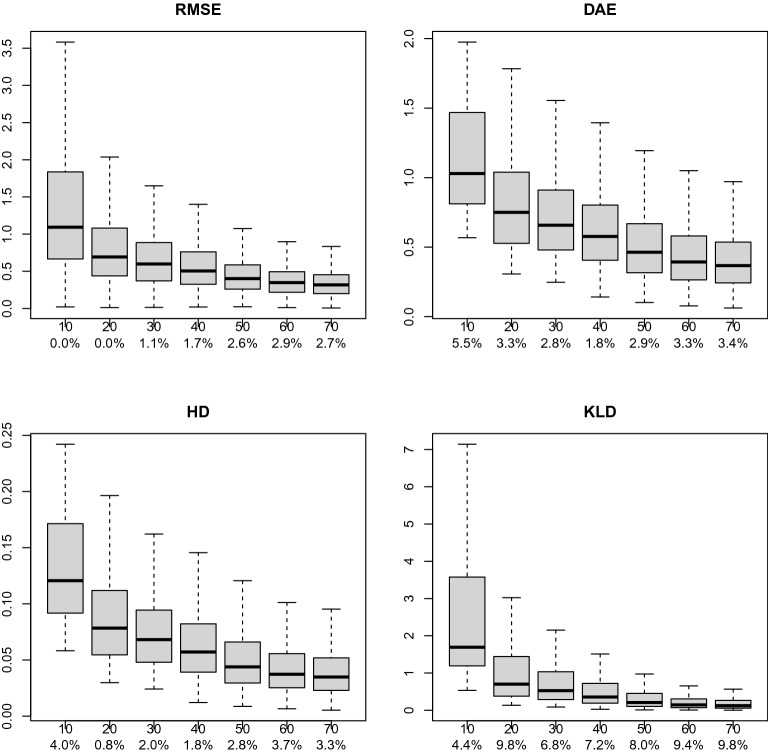
Box-Plots of the RMSE, DAE, HD and KLD simulated values for $d=10, 20, \dots , 70$. Under every *d* value on the horizontal axis, the percentage of points located outside the corresponding whiskers is given.

Our results are visualized in Fig. 3, where the box-plots of the RMS, DAE, HD and KLD values computed above are pictured, for all *d* values considered. As expected, all evaluation measures and their range are decreasing in *d*.

Table 2 provides the average values of the evaluation measures, i.e., $\overline{\mathrm {RMSE}}^{(d)} = \frac{\sum _{\ell } {\mathrm {RMSE}_{\ell }^{(d)}}}{1000}$, and $\overline{\mathrm {DAE}}^{(d)}$, $\overline{\mathrm {HD}}^{(d)}$, and $\overline{\mathrm {KLD}}^{(d)}$, defined analogously. Notice that in our simulation study in case of relatively small number of items ($d\le 30$), we observed simulation cycles for which the Kullback–Leibler divergence was numerically infinite (due to floating point arithmetics), indicating that the divergence between the two compared distributions for these cases was extremely large. In particular, this occurred in 112 cases for $d=10$, 20 cases for $d=20$ and one case for $d=30$ (out of 1000). These cases were excluded from the calculation of the corresponding average KLD-values reported in Table 2.

**Table 2:** Average values of the root-mean-square error (RMSE) for the MLE along with the density approximation error (DAE), the Hellinger distance (HD) and the Kullback–Leibler divergence (KLD) between the density of the MLE-centered normalized posterior distribution and a bivariate standard normal density, based on 1000 simulations of model (19) with $q=2$, for different numbers of items *d*.

*d*	$\overline{\mathrm {RMSE}}^{(d)}$	$\overline{\mathrm {DAE}}^{(d)}$	$\overline{\mathrm {HD}}^{(d)}$	$\overline{\mathrm {KLD}}^{(d)}$
10	1.4105	1.1416	0.1330	3.0803
20	0.8206	0.8267	0.0885	1.2698
30	0.6730	0.7272	0.0750	0.8601
40	0.5658	0.6314	0.0634	0.5922
50	0.4552	0.5222	0.0515	0.3850
60	0.3859	0.4510	0.0439	0.2776
70	0.3530	0.4184	0.0403	0.2303

**Fig. 4:**
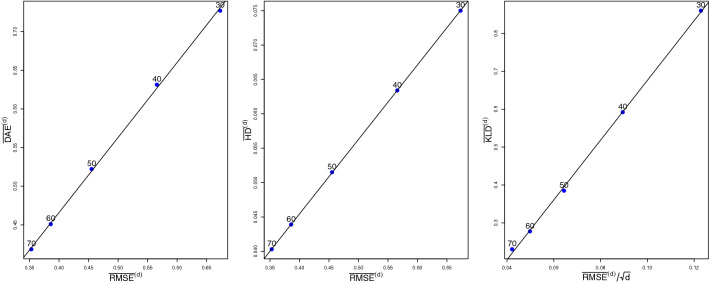
Linear regression of $\overline{\mathrm {DAE}}^{(d)}$ and $\overline{\mathrm {HD}}^{(d)}$ on $\overline{\mathrm {RMSE}}^{(d)}$ and of $\overline{\mathrm {KLD}}^{(d)}$ on $\overline{\mathrm {RMSE}}^{(d)}/\sqrt{d}$ for $d\ge 30$ (s. Table 2).

Figure 4 visualizes the relation of the divergence measures of the normalized posterior from the standardized normal distribution to the RMSE of the MLE for the values of Table 2, pictured for $d\ge 30$. Observe that as *d* increases, $\overline{\mathrm {DAE}}^{(d)}$ and $\overline{\mathrm {HD}}^{(d)}$ are linear in $\overline{\mathrm {RMSE}}^{(d)}$, while $\overline{\mathrm {KLD}}^{(d)}$ is linear in $\overline{\mathrm {RMSE}}^{(d)}/\sqrt{d}$. This is an indication that DAE and HD have the same rate of convergence to zero as RMSE while that of KLD is scaled by $d^{-1/2}$.

Notice that the convergence of the DAE to zero in probability is equivalent to proper centering. Thus, our simulation results suggest that the MLE is a proper centering for the multivariate version of the model of Lee and Bolt (2018) with the parameters we consider.

Simulation studies for other IRT models can be conducted similarly, expecting analogous results.

## Discussion

In this work, we proved the APN of LTs under mild conditions that are fulfilled by a broad class of MIRT models for binary items. Furthermore, we obtained as by-products the existence and consistency of the MLE and the MAP estimator. Note that though the MLE is commonly known as consistent in IRT and MIRT settings, Sinharay (2015) indicated the lack of asymptotic results under milder conditions than some of the usual ones (such as test lengthening by *strictly parallel forms*). Thus, Theorem 5 (i) is a contribution toward this direction.

The distribution $\mathcal {G}$ in Theorem 5 (iii) can be different from $\mathcal {H}$ used in the model. Hence, the asymptotic result above is robust to misspecifications of $\mathcal {H}$ as long as the support is sufficiently large. An interesting task for further investigation, pointed out by one of the reviewers, is the study of the effect of misspecified item response functions.

Under similar mild conditions we provided results on the weak consistency of posterior distributions. In Theorem 6 (ii), we get the existence and consistency of the expected a-posteriori estimator (EAP) for estimating $\varvec{\eta }_0$ as well as for estimating $f(\varvec{\eta }_0)$. To the best of our knowledge, a proof of these properties in such a general setup and under comparably mild or milder conditions on the MIRT model does not exist in the related literature.

Our results are under the assumption of a proper prior $\mathfrak {h}$. This is appropriate in IRT settings, where the prior is a model of the population distribution of the latent traits. However, in a Bayesian framework, improper priors can also be considered. If this is the case, the proper prior assumption in (CS1’[ii]) can be replaced by the following condition if the posterior is still proper

28 $$\begin{aligned} \frac{1}{P^{(d)}({\varvec{\mathsf {Y}}}^{(d)}\mid \varvec{\eta }_0)}\int _{\Theta \setminus B_\delta (\varvec{\eta }_0)}P^{(d)}({\varvec{\mathsf {Y}}}^{(d)}\mid \varvec{\eta })\mathfrak {h}(\varvec{\eta })\,\mathrm {d}\varvec{\eta }=o_{\mathsf {P}_{\varvec{\eta }_0}}\big (d^{-q/2}\big ), \ \ \ \text {for all} \ \ \ \delta >0 . \end{aligned}$$

Condition (28) is sufficient for the derivation of the results stated here and is satisfied by a proper prior.

The APN for a univariate LT for polytomous items was discussed by Chang (1996). The extension of the results for MIRT models with polytomous items is the subject of our current research.

Here, we derived conditions for APN of LTs by generalizing the contribution of Chang and Stout (1993) to $q>1$. The methodology of GGS/IH, discussed in Sect. 4, provides a general framework for APN in various contexts, including MIRT. The results of Ghosal (1997, 1999) are helpful in deriving alternative conditions for APN tailored for MIRT models.

### Supplementary Information

Below is the link to the electronic supplementary material.

Supplementary file 1 (pdf 409 KB)

## Appendix: Proofs of Theorems in Section 7

Here, we prove the main results Theorem 5 and 6 and provide required lemmas for their proof. More detailed versions of all proofs including preliminary results are provided in the web-appendix.

For the proof of Theorem 5 (i) the following lemma is required. This lemma ensures that for $d\rightarrow \infty$, a global maximum of the log-likelihood has to be in an arbitrarily small area around $\varvec{\eta }_0$, thus being the main step to prove the consistency of the MLE.

### Lemma 1

Consider $\{Y_i\}_{i\in \mathbb {N}}\sim {\mathcal {P}}(\varvec{\eta })$ and assume that conditions (CS1’[i]), (CS2’) and (CS3’) are satisfied for a fixed $\varvec{\eta }_0\in \Theta$. Then, for any $\delta >0$ there is a $k(\delta )<0$ so that

$$\begin{aligned} \lim _{d\rightarrow \infty }\mathsf {P}_{\varvec{\eta }_0}\left( \sup _{\varvec{\eta }\in \Theta \backslash B_\delta (\varvec{\eta }_0)}\frac{1}{d}\left( \ell ^{(d)}(\varvec{\eta }\,|\,{\varvec{\mathsf {Y}}}^{(d)})-\ell ^{(d)}(\varvec{\eta }_0\,|\,{\varvec{\mathsf {Y}}}^{(d)})\right) < k(\delta )\right) =1. \end{aligned}$$

### Proof

A more detailed version of the proof can be found in the web-appendix (p. 3).

Consider an arbitrary $\delta >0$. One can show that the associated sequence of item response functions $\{P_i\}_{i\in \mathbb {N}}$ is equicontinuous on each compact set (compare Lemma W.1 in the web-appendix). This implies, applying the strong law of large numbers, that

$$\begin{aligned} \lim _{d\rightarrow \infty }\mathsf {P}_{\varvec{\eta }_0}\left( \sup _{\varvec{\eta }\in K}\frac{1}{d}\left( \ell ^{(d)}(\varvec{\eta }\,|\,{\varvec{\mathsf {Y}}}^{(d)})-\ell ^{(d)}(\varvec{\eta }_0\,|\,{\varvec{\mathsf {Y}}}^{(d)})\right) <c_{K}\right) =1, \end{aligned}$$

for each compact $K\subset \Theta$ for which a $\delta >0$ exists such that $B_\delta (\varvec{\eta }_0)\not \subset K$ with a constant $c_K\le \sup _{\varvec{\eta }\in K}c(\varvec{\eta })/2<0$ (compare the more detailed version of the proof in the web-appendix). This is in particular true for

$$\begin{aligned} K=\Theta _j:=\Big \{\varvec{\eta }\in \Theta :\delta +j\le \Vert \varvec{\eta }-\varvec{\eta }_0\Vert \le \delta +j+1\Big \} \end{aligned}$$

for each $j\in \mathbb {N}_0$ and $\delta >0$. Finally, we get with probability tending to one for $d\rightarrow \infty$ that

$$\begin{aligned}&\sup _{\varvec{\eta }\in \Theta \backslash B_\delta (\varvec{\eta }_0)}\frac{1}{d}\left( \ell ^{(d)}(\varvec{\eta }\,|\,{\varvec{\mathsf {Y}}}^{(d)})-\ell ^{(d)}(\varvec{\eta }_0\,|\,{\varvec{\mathsf {Y}}}^{(d)})\right) \\&\quad \quad =\sup _{j\in \mathbb {N}_0}\Bigg (\sup _{\varvec{\eta }\in \Theta _j}\frac{1}{d}\left( \ell ^{(d)}(\varvec{\eta }\,|\,{\varvec{\mathsf {Y}}}^{(d)})-\ell ^{(d)}(\varvec{\eta }_0\,|\,{\varvec{\mathsf {Y}}}^{(d)})\right) \Bigg )\\&\quad \quad \le \sup _{j\in \mathbb {N}_0} c_j\le \sup _{\varvec{\eta }\in \Theta \setminus B_\delta (\varvec{\eta }_0)}c(\varvec{\eta })/2=:2\cdot k(\delta ). \end{aligned}$$

$$\square $$

### Proof of Theorem 5(i)–(ii)

A more detailed version of the proof can be found in the web-appendix (p. 7).

Analogously to the proof of Corollary 3.1 of Chang and Stout (1991), notice that

A1 $$\begin{aligned} \ell ^{(d)}({\varvec{\hat{\eta }}}_d\,|\,{\varvec{\mathsf {Y}}}^{(d)})-\ell ^{(d)}(\varvec{\eta }_0\,|\,{\varvec{\mathsf {Y}}}^{(d)})=\log \left( \frac{P^{(d)}({\varvec{\mathsf {Y}}}^{(d)}\,|\,{\varvec{\hat{\eta }}}_d)}{P^{(d)}({\varvec{\mathsf {Y}}}^{(d)}\,|\,\varvec{\eta }_0)}\right) \ge 0, \end{aligned}$$

if the MLE exists due to its definition as global maximum. From Lemma 1 follows, that every global maximum of the log-likelihood has to be in every arbitrary small region around $\varvec{\eta }_0$ with probability tending to one for $d\rightarrow \infty$, which implies consistency. The existence of the MLE and further its derivation as solution of the likelihood equations can be shown completely analogous to classical iid cases (e.g., Lehmann & Casella, 1998, Chapter 6, Theorem 5.1, p. 463).

Considering the modified log-likelihood function

$$\begin{aligned} {\tilde{\ell }}^{(d)}(\varvec{\eta }\mid {\varvec{\mathsf {Y}}}^{(d)}))=\ell ^{(d)}(\varvec{\eta }\mid {\varvec{\mathsf {Y}}}^{(d)}) + \log (\mathcal {W}(\varvec{\eta })),\quad \quad \varvec{\eta }\in \Theta ,\, d\in \mathbb {N}, \end{aligned}$$

of part (ii), the consistency is obtained by replacing $\ell$ by ${\tilde{\ell }}$ in Lemma 1 and part (i) of this theorem. $\square$

The following lemma ensures the log-likelihood-ratio can be well approximated by a quadratic form of the test information matrix, which is an essential part for the proof of Theorem 5(iii). Lemma 3 and Corollary 1 provided in the sequel, are additionally required for the proof of Theorem 5(iii) and Theorem 6.

### Lemma 2

Suppose that conditions (CS1’) through (CS5’) hold. Denote by $H_d(\,\cdot \,)$ the Hessian matrix of $\ell ^{(d)}(\cdot \,|\,{\varvec{\mathsf {Y}}}^{(d)})$. Set $\varvec{\Sigma }_d:=\mathcal {I}^{(d)}(\varvec{\eta }_0)^{-1}$, which is estimated by ${\widehat{\varvec{\Sigma }}}_d=\mathcal {I}^{(d)}({\varvec{\hat{\eta }}}_d)^{-1}$, if $\mathcal {I}^{(d)}({\varvec{\hat{\eta }}}_d)^{-1}$ exists, and by ${\widehat{\varvec{\Sigma }}}_d=\mathrm {I}_q$ otherwise, where $\mathrm {I}_q$ is the $q\times q$ identity matrix, $d\in \mathbb {N}$. Then, we have the following.

1. There is a sequence $\{a_d\}_{d\in \mathbb {N}}$, $a_i\in [0,1]$, such that for
  $$\begin{aligned} R_d(\varvec{\eta }):={\widehat{\varvec{\Sigma }}}_d\left( \mathcal {I}^{(d)}({\varvec{\hat{\eta }}}_d)+H_d(\varvec{\eta }^*_d)\right) = \mathrm {I}_q + \mathcal {I}^{(d)}({\varvec{\hat{\eta }}}_d)^{-1}H_d(\varvec{\eta }^*_d) \end{aligned}$$
  where $\varvec{\eta }^*_d:=a_d{\varvec{\hat{\eta }}}_d+(1-a_d)\varvec{\eta }$, it holds with probability tending to 1 for $d\rightarrow \infty$, that
  A2 $$\begin{aligned} \ell ^{(d)}(\varvec{\eta }\,|\,{\varvec{\mathsf {Y}}}^{(d)})-\ell ^{(d)}({\varvec{\hat{\eta }}}_d\,|\,{\varvec{\mathsf {Y}}}^{(d)})&=\frac{1}{2}(\varvec{\eta }-{\varvec{\hat{\eta }}}_d)^{\intercal }H_d(\varvec{\eta }^*_d)(\varvec{\eta }-{\varvec{\hat{\eta }}}_d) \end{aligned}$$
  A3 $$\begin{aligned}&=-\frac{1}{2}(\varvec{\eta }-{\varvec{\hat{\eta }}}_d)^{\intercal }\mathcal {I}^{(d)}({\varvec{\hat{\eta }}}_d)(\mathrm {I}_q-R_d(\varvec{\eta }))(\varvec{\eta }-{\varvec{\hat{\eta }}}_d), \end{aligned}$$
  $$\varvec{\eta }\in \Theta $$.
2. For any $\varepsilon >0$, there is a $\delta >0$ such that
  A4 $$\begin{aligned} \lim _{d\rightarrow \infty }\mathsf {P}_{\varvec{\eta }_0}\left( \sup _{\varvec{\eta }\in B_{\delta }(\varvec{\eta }_0)}\Vert R_d(\varvec{\eta })\Vert <\varepsilon \right) =1, \end{aligned}$$
  where $\Vert \pmb A\Vert$ denotes the *spectral norm* for a matrix $\pmb A$.
3. For any $\varepsilon >0$, there is a $\delta >0$ so that for all $\varvec{\eta }\in B_{\delta }(\varvec{\eta }_0)$
  $$\begin{aligned} \lim _{d\rightarrow \infty }\mathsf {P}_{\varvec{\eta }_0}&\bigg ((1+\varepsilon )Q_d(\varvec{\eta })\le -\frac{1}{2}(\varvec{\eta }-{\varvec{\hat{\eta }}}_d)^{\intercal }\mathcal {I}^{(d)}({\varvec{\hat{\eta }}}_d)(\mathrm {I}_q-R_d(\varvec{\eta }))(\varvec{\eta }-{\varvec{\hat{\eta }}}_d) \nonumber \\ {}&\qquad \le (1-\varepsilon )Q_d(\varvec{\eta }) \bigg )=1, \end{aligned}$$
  where
  $$\begin{aligned} Q_d(\varvec{\eta }):=-\frac{1}{2}(\varvec{\eta }-{\varvec{\hat{\eta }}}_d)^{\intercal }\mathcal {I}^{(d)}({\varvec{\hat{\eta }}}_d)(\varvec{\eta }-{\varvec{\hat{\eta }}}_d), \quad \varvec{\eta }\in B_\delta (\varvec{\eta }_0),\,\,d\in \mathbb {N}. \end{aligned}$$

Recall that if a matrix $\pmb A$ is symmetric and positive definite, then $\Vert \pmb A\Vert =\nu _{\max }(\pmb A)$ and $\Vert \pmb A^{-1}\Vert =\nu _{\min }(\pmb A)^{-1}$ hold, where $\nu _{\max }$ and $\nu _{\min }$ denote the largest and smallest eigenvalues of a matrix.

### Proof

An extended proof is provided in the web-appendix (p. 14).

Equation (A2) follows directly from a second-order Taylor expansion of $\ell ^{(d)}(\varvec{\eta }\,|\,{\varvec{\mathsf {Y}}}^{(d)})$ at ${\varvec{\hat{\eta }}}_d$. Theorem 5(i) and conditions (CS2’) and (CS5’) imply the existence of $\mathcal {I}^{(d)}({\varvec{\hat{\eta }}}_d)^{-1}$ with probability tending to one for $d\rightarrow \infty$ (compare Lemma W.6 in the web-appendix) and, therefore, (A3). Condition (CS5’) further implies for some constant $C_0>0$ and $d\rightarrow \infty$ that

$$\begin{aligned} \Vert R_d(\varvec{\eta })\Vert&=\bigg \Vert \bigg (\frac{1}{d}\mathcal {I}^{(d)}({\varvec{\hat{\eta }}}_d)\bigg )^{-1}\frac{1}{d}\Big (\mathcal {I}^{(d)}({\varvec{\hat{\eta }}}_d)+H_d(\varvec{\eta }^*_d)\Big )\bigg \Vert \\&\le \bigg \Vert \bigg (\frac{1}{d}\mathcal {I}^{(d)}({\varvec{\hat{\eta }}}_d)\bigg )^{-1}\bigg \Vert \cdot \bigg \Vert \frac{1}{d}\Big (\mathcal {I}^{(d)}({\varvec{\hat{\eta }}}_d)+H_d(\varvec{\eta }^*_d)\Big )\bigg \Vert \\&\le \frac{1}{C_0}\bigg \Vert \frac{1}{d}\Big (\mathcal {I}^{(d)}({\varvec{\hat{\eta }}}_d)+H_d(\varvec{\eta }^*_d)\Big )\bigg \Vert . \end{aligned}$$

One can show that the conditions imposed imply that

$$\begin{aligned} \left\{ \frac{1}{d}\mathcal {I}^{(d)}(\,\cdot \,)\right\} _{d\in \mathbb {N}},\quad \quad \left\{ \frac{1}{d}H_d(\,\cdot \,)\right\} _{d\in \mathbb {N}} \end{aligned}$$

are equicontinuous in every compact and convex region in $\Theta$ (compare Lemma W.4 in the web-appendix on p. 11). Kolmogorov’s strong law of large numbers leads then to

$$\begin{aligned} \Vert R_d(\varvec{\eta })\Vert \le C_1\Vert \varvec{\eta }_0-\varvec{\eta }_d^*\Vert +C_2\Vert \varvec{\eta }_0-{\varvec{\hat{\eta }}}_d\Vert +o_{\mathsf {P}_{\varvec{\eta }}}(1), \end{aligned}$$

for $d\rightarrow \infty$ and some appropriate constants $C_1,C_2>0$. Since the MLE is consistent and for every $\varvec{\eta }\in B_\delta (\varvec{\eta }_0)$ it holds

$$\begin{aligned} \Vert \varvec{\eta }_d^*-\varvec{\eta }_0\Vert \le \Vert \varvec{\eta }_0-{\varvec{\hat{\eta }}}_d\Vert +\Vert \varvec{\eta }-\varvec{\eta }_0\Vert , \end{aligned}$$

(recall $\varvec{\eta }^*_d$ lies between $\varvec{\eta }$ and ${\hat{\varvec{\eta }_d}}$), we get for $\varepsilon :=2C_1\delta$

$$\begin{aligned} \sup _{\varvec{\eta }\in B_\delta (\varvec{\eta }_0)}\Vert R_d(\varvec{\eta })\Vert <\varepsilon +o_{\mathsf {P}_{\varvec{\eta }}}(1). \end{aligned}$$

Notice that

$$\begin{aligned} \Big |\pmb x^{\intercal }\pmb A\pmb B\pmb x\Big |\le \sqrt{\frac{\nu _{\max }(\pmb A)}{\nu _{\min }(\pmb A)}}\Vert \pmb B\Vert \pmb x^{\intercal }\pmb A\pmb x, \end{aligned}$$

for every $\pmb x\in \mathbb {R}^q$, symmetric and positive definite $\pmb A\in \mathbb {R}^{q\times q}$ and any further matrix $\pmb B\in \mathbb {R}^{q\times q}$. The final part follows by selecting $\pmb A=\mathcal {I}^{(d)}({\varvec{\hat{\eta }}}_d)$ and $\pmb B=R_d(\varvec{\eta }_d^*)$ for $d\rightarrow \infty$. $\square$

### Lemma 3

Let ${{\tilde{\Phi }}}(B):=\mathsf {P}({\varvec{\mathsf {Z}}}\in B)$ for all $B\in \mathcal {B}^q$ with ${\varvec{\mathsf {Z}}}\sim \mathcal {N}_q({\varvec{\mathsf {0}}},{\varvec{\mathsf {I}}}_q)$. Consider a sequence $\{Y_i\}_{i\in \mathbb {N}}$ for a fixed $\varvec{\eta }_0\in \Theta$, for which conditions (CS1’ [i]), (CS2’) through (CS5’), and either (CS1’ [ii]) or (28) are satisfied. Then, the following holds.

1. For every function *f* that is either absolutely bounded by a constant $c>0$ or for which the integral $\int _{\mathbb {R}^q}f(\varvec{\eta })\mathfrak {h}(\varvec{\eta })\mathrm {d}\varvec{\eta }$ exists, and for every $\delta >0$, it holds
  A5 $$\begin{aligned} \frac{\int _{\mathbb {R}^q\setminus B_\delta (\varvec{\eta }_0)}f(\varvec{\eta })P^{(d)}({\varvec{\mathsf {Y}}}^{(d)}\mid \varvec{\eta })\mathfrak {h}(\varvec{\eta })\,\mathrm {d}\varvec{\eta }}{P^{(d)}({\varvec{\mathsf {Y}}}^{(d)}\mid {\varvec{\hat{\eta }}}_d)\det ({\widehat{\varvec{\Sigma }}}^{1/2}_d)}\overset{\mathsf {P}_{\varvec{\eta }_0}}{\longrightarrow } 0,\quad \quad d\longrightarrow \infty . \end{aligned}$$
2. Consider a sequence $\{G_d\}_{d\in \mathbb {N}}$ with $G_d:(\Theta ,\mathcal {B}(\Theta ))\rightarrow (\Theta ,\mathcal {B}(\Theta ))$ satisfying either
  A6 $$\begin{aligned} \lim _{d\rightarrow \infty }\mathsf {P}_{\varvec{\eta }_0}\left( G_d(B)\subset B_\delta (\varvec{\eta }_0)\right) =1 , \ \ \ \text {for all} \ \ \ \delta >0, \end{aligned}$$
  or
  A7 $$\begin{aligned} \lim _{d\rightarrow \infty }\mathsf {P}_{\varvec{\eta }_0}\left( G_d(B)\supset B_\delta (\varvec{\eta }_0)\right) =1, \ \ \ \text {for one} \ \ \ \delta >0, \end{aligned}$$
  for all bounded $B\in \mathcal {B}^q$. Then, for $d\longrightarrow \infty$, it holds
  $$\begin{aligned}&\frac{\int _{G_d(B)}P^{(d)}({\varvec{\mathsf {Y}}}^{(d)}\mid \varvec{\eta })\mathfrak {h}(\varvec{\eta })\,\mathrm {d}\varvec{\eta }}{P^{(d)}({\varvec{\mathsf {Y}}}^{(d)}\mid {\varvec{\hat{\eta }}}_d)\det ({\hat{\varvec{\Sigma }}}^{1/2}_d)}\\&\quad -{{\tilde{\Phi }}}\left( \mathcal {I}^{(d)}({\varvec{\hat{\eta }}}_d)^{1/2}(G_d(B)-{\varvec{\hat{\eta }}}_d)\right) \mathfrak {h}(\varvec{\eta }_0)(2\pi )^{q/2}=o_{\mathsf {P}_{\varvec{\eta }_0}} (1). \end{aligned}$$
  In particular, in case of (A7), it holds
  $$\begin{aligned} {{\tilde{\Phi }}}\left( \mathcal {I}^{(d)}({\varvec{\hat{\eta }}}_d)^{1/2}(G_d(B)-{\varvec{\hat{\eta }}}_d)\right) = \mathsf {P}\left( {\varvec{\mathsf {Z}}}\in \mathcal {I}^{(d)}({\varvec{\hat{\eta }}}_d)^{1/2}(G_d(B)-{\varvec{\hat{\eta }}}_d)\right) \ \overset{\mathsf {P}_{\varvec{\eta }_0}}{\longrightarrow } \ 1. \end{aligned}$$

### Proof

A more detailed version of the proof can be found in the web-appendix (p. 17). 1. With regard to the left-hand side of (A5), note that, in terms of the log-likelihood function, it can be written as

A8 $$\begin{aligned}&\frac{\int _{\mathbb {R}^q\setminus B_\delta (\varvec{\eta }_0)}f(\varvec{\eta })P^{(d)}({\varvec{\mathsf {Y}}}^{(d)}\mid \varvec{\eta })\mathfrak {h}(\varvec{\eta })\,\mathrm {d}\varvec{\eta }}{P^{(d)}({\varvec{\mathsf {Y}}}^{(d)}\mid {\varvec{\hat{\eta }}}_d)\det ({\widehat{\varvec{\Sigma }}}^{1/2}_d)}=\exp \bigg (\ell ^{(d)}(\varvec{\eta }_0\,|{\varvec{\mathsf {Y}}}^{(d)})-\ell ^{(d)}({\varvec{\hat{\eta }}}_d\,|{\varvec{\mathsf {Y}}}^{(d)})\bigg )\frac{ {T_d } }{\det \big ({\widehat{\varvec{\Sigma }}}^{1/2}_d\big )}, \end{aligned}$$

where

$$\begin{aligned} {T_d }:=\int _{\mathbb {R}^q\backslash B_\delta (\varvec{\eta }_0)}f(\varvec{\eta })\exp \bigg (\ell ^{(d)}(\varvec{\eta }\,|{\varvec{\mathsf {Y}}}^{(d)})-\ell ^{(d)}(\varvec{\eta }_0\,|{\varvec{\mathsf {Y}}}^{(d)})\bigg )\mathfrak {h}(\varvec{\eta }) \,\mathrm {d}\varvec{\eta }, \end{aligned}$$

while it always fulfills

$$\begin{aligned} \left| \frac{\int _{\mathbb {R}^q\setminus B_\delta (\varvec{\eta }_0)}f(\varvec{\eta })P^{(d)}({\varvec{\mathsf {Y}}}^{(d)}\mid \varvec{\eta })\mathfrak {h}(\varvec{\eta })\,\mathrm {d}\varvec{\eta }}{P^{(d)}({\varvec{\mathsf {Y}}}^{(d)}\mid {\varvec{\hat{\eta }}}_d)\det ({\widehat{\varvec{\Sigma }}}^{1/2}_d)}\right| \le \left| \frac{{T_d }}{\det \big ({\widehat{\varvec{\Sigma }}}^{1/2}_d\big )}\right| . \end{aligned}$$

If $\mathcal {H}$ is improper and *f* is bounded by a constant, (28) directly implies

$$\begin{aligned} \left| \frac{{T_d }}{\det \big ({\widehat{\varvec{\Sigma }}}^{1/2}_d\big )}\right| =o_{\mathsf {P}_{\varvec{\eta }_0}}(1). \end{aligned}$$

In any other case, Lemma 1 leads to

$$\begin{aligned} \lim _{d\rightarrow \infty }\mathsf {P}_{\varvec{\eta }_0}\bigg (|{T_d }|\le \mathsf {E}(|f(\varvec{\eta })|)\exp (dc(\delta ))\bigg )=1. \end{aligned}$$

Finally, from the polynomial grows of $\det \big ({\widehat{\varvec{\Sigma }}}^{1/2}_d\big )^{-1}$ , it follows

$$\begin{aligned} \frac{\mathsf {E}(|f(\varvec{\eta })|)\exp (dc(\delta ))}{\det \big ({\widehat{\varvec{\Sigma }}}^{1/2}_d\big )}\overset{\mathsf {P}_{\varvec{\eta }_0}}{\longrightarrow }0. \end{aligned}$$

2. Let $B\in \mathcal {B}^q$ be an arbitrary bounded Borel set and define for $\delta >0$ and $d\in \mathbb {N}$ the set

$$\begin{aligned} M_{\delta ,d}:=B_\delta (\varvec{\eta }_0)\cap G_d(B) \end{aligned}$$

and the integral

$$\begin{aligned} {V_d }:=&\int _{M_{\delta ,d}}P^{(d)}({\varvec{\mathsf {Y}}}^{(d)}\,|\,\varvec{\eta })\mathfrak {h}(\varvec{\eta }) \,\mathrm {d}\varvec{\eta }. \end{aligned}$$

Using the definition of $R_d(\varvec{\eta })$ in Lemma 2, it holds

A9 $$\begin{aligned} \begin{aligned}&\frac{{V_d }}{P^{(d)}({\varvec{\mathsf {Y}}}^{(d)}\,|\,{\varvec{\hat{\eta }}}_d)\det \big ({\widehat{\varvec{\Sigma }}}^{1/2}_d\big )}\\ {}&=\frac{\mathfrak {h}(\varvec{\eta }_0)}{\det \big ({\widehat{\varvec{\Sigma }}}^{1/2}_d\big )}\int _{M_{\delta ,d}}\frac{\mathfrak {h}(\varvec{\eta })}{\mathfrak {h}(\varvec{\eta }_0)}\exp \Bigg (-\frac{1}{2}(\varvec{\eta }-{\varvec{\hat{\eta }}}_d)^{\intercal }\mathcal {I}^{(d)}({\varvec{\hat{\eta }}}_d)(\mathrm {I}-R_d(\varvec{\eta }))(\varvec{\eta }-{\varvec{\hat{\eta }}}_d)\Bigg )\,\mathrm {d}\varvec{\eta }. \end{aligned} \end{aligned}$$

By (CS1’), i.e., the continuity of $\mathfrak {h}$ and $\mathfrak {h}(\varvec{\eta }_0)>0$, it follows that for every $\varepsilon _1>0$, it exists a $\delta _1>0$, such that

A10 $$\begin{aligned} 1-\varepsilon _1\le \inf _{\varvec{\eta }\in M_{\delta _1,d}}\frac{\mathfrak {h}(\varvec{\eta })}{\mathfrak {h}(\varvec{\eta }_0)}\le \sup _{\varvec{\eta }\in M_{\delta _1,d}}\frac{\mathfrak {h}(\varvec{\eta })}{\mathfrak {h}(\varvec{\eta }_0)}\le 1+\varepsilon _1. \end{aligned}$$

Furthermore, by Lemma 2 we get for any $\varepsilon _2>0$ and appropriate $\delta _2=\delta _2(\varepsilon _2)>0$:

A11 $$\begin{aligned}&(1-o_{\mathsf {P}_{\varvec{\eta }_0}}(1))\int _{M_{\delta _2,d}}\exp \Bigg (-\frac{1+\varepsilon _2}{2}(\varvec{\eta }-{\varvec{\hat{\eta }}}_d)^{\intercal }\mathcal {I}^{(d)}({\varvec{\hat{\eta }}}_d)(\varvec{\eta }-{\varvec{\hat{\eta }}}_d)\Bigg )\,\mathrm {d}\varvec{\eta }\nonumber \\&\le \int _{M_{\delta _2,d}}\exp \Bigg (-\frac{1}{2}(\varvec{\eta }-{\varvec{\hat{\eta }}}_d)^{\intercal }\mathcal {I}^{(d)}({\varvec{\hat{\eta }}}_d)(\mathrm {I}-R_d(\varvec{\eta }^*_d))(\varvec{\eta }-{\varvec{\hat{\eta }}}_d)\Bigg )\,\mathrm {d}\varvec{\eta }\\&\le (1+o_{\mathsf {P}_{\varvec{\eta }_0}}(1))\int _{M_{\delta _2,d}}\exp \Bigg (-\frac{1-\varepsilon _2}{2}(\varvec{\eta }-{\varvec{\hat{\eta }}}_d)^{\intercal }\mathcal {I}^{(d)}({\varvec{\hat{\eta }}}_d)(\varvec{\eta }-{\varvec{\hat{\eta }}}_d)\Bigg )\,\mathrm {d}\varvec{\eta }.\nonumber \end{aligned}$$

Next, (A10) and (A11) imply

A12 $$\begin{aligned}&{{\tilde{\Phi }}}\bigg (\sqrt{1+\varepsilon _2}\mathcal {I}^{(d)}({\varvec{\hat{\eta }}}_d)^{1/2}\big (M_{\delta _2,d}-{\varvec{\hat{\eta }}}_d\big )\bigg )\mathfrak {h}(\varvec{\eta }_0)(2\pi )^{q/2}\frac{1-\varepsilon _1}{(1+\varepsilon _2)^{q/2}}\big (1-o_{\mathsf {P}_{\varvec{\eta }_0}}(1)\big )\nonumber \\&\le \frac{{V_d }}{P^{(d)}({\varvec{\mathsf {Y}}}^{(d)}\,|\,{\varvec{\hat{\eta }}}_d)\det \big ({\widehat{\varvec{\Sigma }}}^{1/2}_d\big )}\\&\le {{\tilde{\Phi }}}\bigg (\sqrt{1-\varepsilon _2}\mathcal {I}^{(d)}({\varvec{\hat{\eta }}}_d)^{1/2}\big (M_{\delta _2,d}-{\varvec{\hat{\eta }}}_d\big )\bigg )\mathfrak {h}(\varvec{\eta }_0)(2\pi )^{q/2}\frac{1+\varepsilon _1}{(1-\varepsilon _2)^{q/2}}\big (1+o_{\mathsf {P}_{\varvec{\eta }_0}}(1)\big ).\nonumber \end{aligned}$$

In the case of (A6), it holds $\lim _{d\rightarrow \infty }\mathsf {P}_{\varvec{\eta }_0}\big ( G_d(B)=M_{\delta _2,d}\big )=1$. Selecting $\varepsilon _1$ and $\varepsilon _2$ arbitrarily small leads to

$$\begin{aligned} \frac{\int _{G_d(B)}P^{(d)}({\varvec{\mathsf {Y}}}^{(d)}\mid \varvec{\eta })\mathfrak {h}(\varvec{\eta })\,\mathrm {d}\varvec{\eta }}{P^{(d)}({\varvec{\mathsf {Y}}}^{(d)}\mid {\varvec{\hat{\eta }}}_d)\det ({\hat{\varvec{\Sigma }}}^{1/2}_d)}={{\tilde{\Phi }}}(\mathcal {I}^{(d)}({\varvec{\hat{\eta }}}_d)^{1/2}(G_d(B)-{\varvec{\hat{\eta }}}_d))\mathfrak {h}(\varvec{\eta }_0)(2\pi )^{q/2}+o_{\mathsf {P}_{\varvec{\eta }_0}}(1). \end{aligned}$$

In the case of equation (A7), we get for each $\delta _2<\delta$: $\lim _{d\rightarrow \infty }\mathsf {P}_{\varvec{\eta }_0}\big ( B_{\delta _2}(\varvec{\eta }_0)=M_{\delta _2,d}\big )=1$. Condition (CS5’) implies

$$\begin{aligned} {{\tilde{\Phi }}}\bigg (\sqrt{1+\varepsilon _2}\mathcal {I}^{(d)}({\varvec{\hat{\eta }}}_d)^{1/2}\big (B_{\delta _2}(\varvec{\eta }_0)-{\varvec{\hat{\eta }}}_d\big )\bigg )\overset{\mathsf {P}_{\varvec{\eta }_0}}{\longrightarrow }1. \end{aligned}$$

Finally, the further valid selection of arbitrary small $\varepsilon _1,\varepsilon _2>0$ in (A12) and the application of Lemma 3(1.) on $f={11}_{G_d(B)\setminus B_{\delta _2}(\varvec{\eta }_0)}$ completes the proof. $\square$

For $G_d(B):=\mathbb {R}^q$, Lemma 3(2.) leads directly to the following Corollary.

### Corollary 1

Suppose a sequence $\{Y_i\}_{i\in \mathbb {N}}$ for a fixed $\varvec{\eta }_0\in \Theta$, for which conditions (CS1’) through (CS5’) hold. Then holds for $d\rightarrow \infty$

$$\begin{aligned} \Bigg (\frac{P({\varvec{\mathsf {Y}}}^{(d)})}{P^{(d)}({\varvec{\mathsf {Y}}}^{(d)}\,|\,{\varvec{\hat{\eta }}}_d)\det \big ({\widehat{\varvec{\Sigma }}}^{1/2}_d\big )}\Bigg )^{-1}\ \overset{\mathsf {P}_{\varvec{\eta }_0}}{\longrightarrow } \ \frac{1}{\mathfrak {h}(\varvec{\eta }_0)(2\pi )^{q/2}}. \end{aligned}$$

### Proof of Theorem 5(iii)

An extended proof can be found in the web-appendix (p. 21).

Analogously to the proof of Lemma 2, we can assume without loss of generality that $\mathcal {I}^{(d)}({\varvec{\hat{\eta }}}_d)^{-1/2}$ exists. Set

$$\begin{aligned} G_d(B):=\bigg \{{\widehat{\varvec{\Sigma }}}^{1/2}_d\pmb x+{\varvec{\hat{\eta }}}_d\,:\,\pmb x\in B\bigg \}\equiv \mathcal {I}^{(d)}({\varvec{\hat{\eta }}}_d)^{-1/2}B+{\varvec{\hat{\eta }}}_d,B\in \mathcal {B}^q,\,\,d\in \mathbb {N}. \end{aligned}$$

Then, (26) for bounded *B* follows directly from the reformulation

$$\begin{aligned}&\mathsf {P}\left( \left. \mathcal {I}^{(d)}({\varvec{\hat{\eta }}}_d)^{1/2}\left( \varvec{\eta }-{\varvec{\hat{\eta }}}_d\right) \in B \,\right| \, {\varvec{\mathsf {Y}}}^{(d)}\right) \\&\quad \quad =\frac{\int _{G_d(B)}P^{(d)}({\varvec{\mathsf {Y}}}^{(d)}\,|\,\varvec{\eta })\mathfrak {h}(\varvec{\eta }) \,\mathrm {d}\varvec{\eta }}{P^{(d)}({\varvec{\mathsf {Y}}}^{(d)}\,|\,{\varvec{\hat{\eta }}}_d)\det \big ({\widehat{\varvec{\Sigma }}}^{1/2}_d\big )}\Bigg (\frac{P({\varvec{\mathsf {Y}}}^{(d)})}{P^{(d)}({\varvec{\mathsf {Y}}}^{(d)}\,|\,{\varvec{\hat{\eta }}}_d)\det \big ({\widehat{\varvec{\Sigma }}}^{1/2}_d\big )}\Bigg )^{-1} \end{aligned}$$

due to Lemma 3 part 2 and Corollary 1. The case of unbounded *B* can be shown by considering a decomposition $B=\bigcup _{m\in \mathbb {N}}B_m$ for bounded and pairwise disjoint $\{B_m\}_{m\in \mathbb {N}}$ (compare to the web-appendix, p. 21–22). Next, set for all $d\in \mathbb {N}$, $\varepsilon >0$ and $\varvec{\eta }'\in \Theta$

$$\begin{aligned} H_{d,\varepsilon }(\varvec{\eta }'):=\mathsf {P}\Bigg (\Big |\int _{G_d(B)}h(\varvec{\eta }\,|\,{\varvec{\mathsf {Y}}}^{(d)})\,\mathrm {d}\varvec{\eta }- \int _B\phi (\pmb x)\,\mathrm {d}(\pmb x)\Big |>\varepsilon \,\Bigg |\,\varvec{\eta }_0=\varvec{\eta }'\Bigg ), \end{aligned}$$

where $\phi$ is the pdf of $\mathcal {N}_q({\varvec{\mathsf {0}}}, {\varvec{\mathsf {I}}}_q)$. Then, (27) follows from

$$\begin{aligned} \lim _{d\rightarrow \infty }\mathsf {P}\Bigg (\Big |\int _{G_d(B)}h(\varvec{\eta }\,|\,{\varvec{\mathsf {Y}}}^{(d)})\,\mathrm {d}\varvec{\eta }-\int _B\phi (\pmb x)\,\mathrm {d}(\pmb x)\Big |>\varepsilon \Bigg )&=\lim _{d\rightarrow \infty }\int _{\Theta } H_{d,\varepsilon }\,\mathrm {d}\mathcal {G}\\&=\int _{\Theta }\lim _{d\rightarrow \infty } H_{d,\varepsilon }\,\mathrm {d}\mathcal {G}=0, \end{aligned}$$

for each $\varepsilon >0$, which is valid due to (26) and Lebesgue’s theorem of dominated convergence. $\square$

### Proof of Theorem 6

A more detailed proof is provided in the web-appendix (p. 23).

Part (i) follows directly from Lemma 3 and Corollary 1 by using the reformulation

A13 $$\begin{aligned} \mathcal {H}(B|\,{\varvec{\mathsf {Y}}}^{(d)})=\frac{\int _{B}P^{(d)}({\varvec{\mathsf {Y}}}^{(d)}\,|\,\varvec{\eta })\mathfrak {h}(\varvec{\eta }) \,\mathrm {d}\varvec{\eta }}{P^{(d)}({\varvec{\mathsf {Y}}}^{(d)}\,|\,{\varvec{\hat{\eta }}}_d)\det \big ({\widehat{\varvec{\Sigma }}}^{1/2}_d\big )}\Bigg (\frac{P({\varvec{\mathsf {Y}}}^{(d)})}{P^{(d)}({\varvec{\mathsf {Y}}}^{(d)}\,|\,{\varvec{\hat{\eta }}}_d)\det \big ({\widehat{\varvec{\Sigma }}}^{1/2}_d\big )}\Bigg )^{-1}, \end{aligned}$$

for an arbitrary $B\in \mathcal {B}^q$ with $\varvec{\eta }_0\not \in \partial B$.

Next, we prove part (ii). In a first step, the existence of $\mathsf {E}(f(\varvec{\eta })\mid {\varvec{\mathsf {Y}}}^{(d)})$ for all functions $f:\Theta \rightarrow \mathbb {R}$, which are continuous and for which the integral $\int _{\Theta }f(\varvec{\eta })\mathfrak {h}(\varvec{\eta })\,\mathrm {d}\varvec{\eta }$ exists, will be proved. In a second step its consistency for $f(\varvec{\eta }_0)$ will be discussed.

For every $d\in \mathbb {N}$, it holds

$$\begin{aligned} \int _\Theta |f(\varvec{\eta })|\mathcal {H}(\mathrm {d}\varvec{\eta }\mid {\varvec{\mathsf {y}}}^{(d)})&=\frac{\int _\Theta |f(\varvec{\eta })|P^{(d)}({\varvec{\mathsf {y}}}^{(d)}\mid \varvec{\eta })\mathfrak {h}(\varvec{\eta })\,\mathrm {d}\varvec{\eta }}{P^{(d)}({\varvec{\mathsf {y}}}^{(d)})}\le \frac{\int _\Theta |f(\varvec{\eta })|\mathfrak {h}(\varvec{\eta })\,\mathrm {d}\varvec{\eta }}{P^{(d)}({\varvec{\mathsf {y}}}^{(d)})}<\infty , \end{aligned}$$

for all ${\varvec{\mathsf {y}}}^{(d)}\in \{0,1\}^d$, since $P^{(d)}({\varvec{\mathsf {y}}}^{(d)}\mid \varvec{\eta })\in (0,1)$, $P^{(d)}({\varvec{\mathsf {y}}}^{(d)})$ is positive and independent of $\varvec{\eta }\in \Theta$, and $\int _{\Theta }f(\varvec{\eta })\mathfrak {h}(\varvec{\eta })\,\mathrm {d}\varvec{\eta }$ exists if and only if $\int _{\Theta }f(\varvec{\eta })\mathfrak {h}(\varvec{\eta })\,\mathrm {d}\varvec{\eta }$ exists. Hence, $\mathsf {E}(f(\varvec{\eta })\mid {\varvec{\mathsf {Y}}}^{(d)})$ exists. Furthermore, it remains integrable for $d\rightarrow \infty$, as shown next. Notice that the last statement does not follow directly, since $P^{(d)}({\varvec{\mathsf {y}}}^{(d)})\longrightarrow 0$ for any sequence $\{y_{i}\}_{i\in \mathbb {N}}$ and $d\longrightarrow \infty$.

Adjusting representation (A13), we have

$$\begin{aligned} \int _\Theta |f(\varvec{\eta })|\mathcal {H}(\mathrm {d}\varvec{\eta }\mid {\varvec{\mathsf {Y}}}^{(d)})=\frac{\int _\Theta |f(\varvec{\eta })|P^{(d)}({\varvec{\mathsf {Y}}}^{(d)}\mid \varvec{\eta }) \mathfrak {h}(\varvec{\eta })\,\mathrm {d}\varvec{\eta }}{P^{(d)}({\varvec{\mathsf {Y}}}^{(d)} \mid {\varvec{\hat{\eta }}}_d)\det ({\widehat{\varvec{\Sigma }}}_d)^{1/2}} \left( \frac{P^{(d)}({\varvec{\mathsf {Y}}}^{(d)})}{P^{(d)}({\varvec{\mathsf {Y}}}^{(d)} \mid {\varvec{\hat{\eta }}}_d)\det \big ({\widehat{\varvec{\Sigma }}}_d\big )^{1/2}}\right) ^{-1}. \end{aligned}$$

Lemma 3 and Corollary 1 imply

$$\begin{aligned} \lim _{d\rightarrow \infty } \mathsf {P}_{\varvec{\eta }_0}\bigg (\int _\Theta |f(\varvec{\eta })|\mathcal {H}(\mathrm {d}\varvec{\eta }\mid {\varvec{\mathsf {Y}}}^{(d)})<C_1\bigg )=1, \end{aligned}$$

with $\sup _{\varvec{\eta }\in B_\delta (\varvec{\eta }_0)}|f(\varvec{\eta })|=:C_1<\infty$ for an arbitrary $\delta >0$.

Further, for every $\delta >0$ it holds

$$\begin{aligned}&\Bigg |\int _\Theta f(\varvec{\eta })\mathcal {H}(\mathrm {d}\varvec{\eta }\mid {\varvec{\mathsf {Y}}}^{(d)})-f(\varvec{\eta }_0)\Bigg |=\Bigg |\int _\Theta f(\varvec{\eta })\mathcal {H}(\mathrm {d}\varvec{\eta }\mid {\varvec{\mathsf {Y}}}^{(d)})-f(\varvec{\eta }_0)\mathcal {H}(\,\Theta \mid {\varvec{\mathsf {Y}}}^{(d)})\Bigg |\\&\quad \quad \le \Bigg |\int _{B_\delta (\varvec{\eta }_0)} \big (f(\varvec{\eta })-f(\varvec{\eta }_0)\big )\mathcal {H}(\mathrm {d}\varvec{\eta }\mid {\varvec{\mathsf {Y}}}^{(d)}) \Bigg |+ \Bigg |\int _{\Theta \setminus B_\delta (\varvec{\eta }_0)} f(\varvec{\eta })\mathcal {H}(\mathrm {d}\varvec{\eta }\mid {\varvec{\mathsf {Y}}}^{(d)}) \Bigg |\\ {}&\quad \quad \quad \quad + |f(\varvec{\eta }_0)|\cdot \mathcal {H}(\,\Theta \setminus B_\delta (\varvec{\eta }_0)\mid {\varvec{\mathsf {Y}}}^{(d)}), \end{aligned}$$

where the last two terms converge to zero in probability by Lemma 3, Corollary 1 and part (i). Last, the continuity of *f* implies for each $\varepsilon >0$ and appropriate $\delta ' =\delta '(\varepsilon )$:

$$\begin{aligned} \Bigg |\int _{B_{\delta '}(\varvec{\eta }_0)} \big (f(\varvec{\eta })-f(\varvec{\eta }_0)\big )\mathcal {H}(\mathrm {d}\varvec{\eta }\mid {\varvec{\mathsf {Y}}}^{(d)}) \Bigg |\le \varepsilon \cdot \mathcal {H}(B_{\delta '}(\varvec{\eta }_0)\mid {\varvec{\mathsf {Y}}}^{(d)})\le \varepsilon . \end{aligned}$$

The second part follows directly by considering the mappings $\varvec{\eta }\mapsto \eta _j$, $j\in \{1,\ldots ,q\}$, in the first part, which are continuous and by assumption integrable. $\square$
