# Supplementary material for: Asymptotic Posterior Normality of Multivariate Latent Traits in an IRT Model
Source: Psychometrika. 2022 Feb 11;87(3):1146–72. doi: 10.1007/s11336-021-09838-2 (PMC9433366; doi:10.1007/s11336-021-09838-2)
Supplement: Supplementary file 1 — (pdf 409 KB) [file 11336_2021_9838_MOESM1_ESM.pdf]

Web-Appendix of
Asymptotic Posterior Normality of Multivariate Latent Traits in an IRT Model

Mia J. K. Kornely and Maria Kateri
RWTH Aachen University

Psychometrika
https://doi.org/10.1007/s11336-021-09838-2

Detailed Proofs

In this web-appendix, we provide detailed proofs of the main results (Theorems 6 and 7) and the required Lemmas 1–3, given in the appendix of the paper, along with some necessary preliminary lemmas. For a preliminary version of the proofs see Chapter 3 in Kornely (2021).

The proofs that follow rely on Kolmogorov's version of the strong law of large numbers (SLLN) for non-identically distributed random variables (cf. Serfling, 1980), which, for completeness, is given below.

Theorem W.1. (Kolmogorov's strong law of large numbers) Let $\{X_i\}_{i\in\mathbb{N}}$ be a sequence of independent random variables with $\mathsf{E}(X_i) = \mu_i \in \mathbb{R}$ and $0 < \mathsf{Var}(X_i) = \sigma^2 < \infty$. If

$$\sum_{i=1}^{\infty} \frac{\sigma_i^2}{i^2} < \infty,$$

then

$$\frac{1}{d}\sum_{i=1}^{d} X_i - \frac{1}{d}\sum_{i=1}^{d} \mu_i \to 0, \qquad \mathsf{P}\text{ - almost surely}$$

for $d \to \infty$.

A preliminary result, needed for the proof of Lemma 1, is the equicontinuity of the item response functions on every compact subset of Θ, which is shown next.

Lemma W.1. Let a sequence of mappings $\{P_i\}_{i\in\mathbb{N}}$ be given so that the condition (CS2′) is satisfied. Additionally, let K be an arbitrary compact set for which a convex and compact set K^+ exists such that $K \subseteq K^+ \subseteq \Theta$. Then the family of mappings $\{P_i|_K\}_{i\in\mathbb{N}}$ is *equicontinuous*, where $P_i|_K$ denotes the restriction of P_i on K for $i \in \mathbb{N}$.

Proof. The proof is based on arguments of Chang and Stout (1991, Lemma 3.1).

Denote by ∇P_i the gradient of P_i. Then, for a point $\tilde{\boldsymbol{\eta}} \in \{(1-c)\boldsymbol{\eta} + c\boldsymbol{\eta}',\, c \in [0,1]\}$ on the line between $\boldsymbol{\eta}$ and $\boldsymbol{\eta}'$, we get from the multivariate mean value theorem and the Cauchy–Schwarz inequality that

$$|P_i(\boldsymbol{\eta}) - P_i(\boldsymbol{\eta}')| = |\nabla P_i(\tilde{\boldsymbol{\eta}})^{\intercal}(\boldsymbol{\eta} - \boldsymbol{\eta}')| \leq \|\nabla P_i(\tilde{\boldsymbol{\eta}})\| \cdot \|\boldsymbol{\eta} - \boldsymbol{\eta}'\|.$$

Due to (CS2′), if restricted to K^+, all $\left|\frac{\partial P_i}{\partial \eta_k}\right|$ are uniformly bounded for all $i \in \mathbb{N}$, $1 \leq k \leq q$. Then there is a finite number $\zeta(K^+)$ such that

$$\sup_{(i,\boldsymbol{\eta}) \in \mathbb{N} \times K^+} \|\nabla P_i(\boldsymbol{\eta})\| = \zeta(K^+)$$

and hence for all $\boldsymbol{\eta}, \boldsymbol{\eta}' \in K^+$

$$|P_i(\boldsymbol{\eta}) - P_i(\boldsymbol{\eta}')| \leq \zeta(K^+)\|\boldsymbol{\eta} - \boldsymbol{\eta}'\| \quad \text{for all } i \in \mathbb{N}. \tag{W1}$$

Especially

$$|P_i(\boldsymbol{\eta}) - P_i(\boldsymbol{\eta}')| < \varepsilon \tag{W2}$$

holds for all $\boldsymbol{\eta}, \boldsymbol{\eta}' \in K^+$ with $\|\boldsymbol{\eta} - \boldsymbol{\eta}'\| < \delta = \frac{\varepsilon}{\zeta(K^+)}$, for $\varepsilon > 0$ and all $i \in \mathbb{N}$. Notice, (W1) and (W2) are still true, if we take $\boldsymbol{\eta}, \boldsymbol{\eta}' \in K$, where $K \subset K^+$. Hence, the family of maps $\{P_i|_K\}_{i \in \mathbb{N}}$ is equicontinuous for any compact set K, for which a convex and compact set $K^+ \subset \Theta$ exists such that $K \subset K^+$.

Notice that sets K, as considered in Lemma W.1, are in fact all compact subsets of Θ due to the requird convexity of Θ.

Kolmogorov's SLLN can be used to show that that $\frac{1}{d}\left(\ell^{(d)}(\boldsymbol{\eta} \,|\, \mathbf{Y}^{(d)}) - \ell^{(d)}(\boldsymbol{\eta}_0 \,|\, \mathbf{Y}^{(d)})\right)$ is asymptotically negative, as shown in the next lemma. Using the equicontinuity of the item response functions, i.e. Lemma W.1, this can then extended to the supremum of $\boldsymbol{\eta} \in \Theta$ to obtain Lemma 1.

Lemma W.2. Let a sequence $\{Y_i\}_{i \in \mathbb{N}} \sim \mathcal{P}(\boldsymbol{\eta}_0)$, for a sequence $\{P_i\}_{i \in \mathbb{N}}$ and a fixed $\boldsymbol{\eta}_0 \in \Theta$, be given so that the conditions (CS1′[i]), (CS2′) and (CS3′) are satisfied. Then

$$\limsup_{d \to \infty} \frac{1}{d}\left(\ell^{(d)}(\boldsymbol{\eta} \,|\, \mathbf{Y}^{(d)}) - \ell^{(d)}(\boldsymbol{\eta}_0 \,|\, \mathbf{Y}^{(d)})\right) \leq c(\boldsymbol{\eta}) < 0, \quad \mathsf{P}_{\boldsymbol{\eta}_0}\text{–almost surely,} \tag{W3}$$

holds for every $\boldsymbol{\eta} \in \Theta \setminus \{\boldsymbol{\eta}_0\}$, where $c(\boldsymbol{\eta})$ is the constant of condition (CS3′).

Proof. The proof is based on arguments of Chang and Stout (1991, Lemma 3.1).

Notice that for each $i \in \mathbb{N}$,

$$\begin{aligned}\mathsf{Var}_{\boldsymbol{\eta}_0}(\log Z_i(\boldsymbol{\eta},\boldsymbol{\eta}_0)) &= \mathsf{Var}_{\boldsymbol{\eta}_0}(Y_i)(\lambda_i(\boldsymbol{\eta}) - \lambda_i(\boldsymbol{\eta}_0))^2 \\ &= P_i(\boldsymbol{\eta}_0)(1 - P_i(\boldsymbol{\eta}_0)(\lambda_i(\boldsymbol{\eta}) - \lambda_i(\boldsymbol{\eta}_0))^2\end{aligned}$$

holds. Due to (CS2′), for each $\boldsymbol{\eta} \in \Theta$ there is a constant $0 < M(\boldsymbol{\eta},\boldsymbol{\eta}_0) < \infty$ such that

$$\sup_{i\in\mathbb{N}} \mathsf{Var}_{\boldsymbol{\eta}_0}(\log Z_i(\boldsymbol{\eta},\boldsymbol{\eta}_0)) \leq 2\sup_{i\in\mathbb{N}} |\lambda_i(\boldsymbol{\eta})|^2 + 2\sup_{i\in\mathbb{N}} |\lambda_i(\boldsymbol{\eta}_0)|^2 = M(\boldsymbol{\eta},\boldsymbol{\eta}_0) < \infty.$$

Hence, we get

$$\sum_{i=1}^{\infty} \frac{\mathsf{Var}_{\boldsymbol{\eta}_0}(\log Z_i(\boldsymbol{\eta},\boldsymbol{\eta}_0))}{i^2} \leq M(\boldsymbol{\eta},\boldsymbol{\eta}_0) \sum_{i=1}^{\infty} \frac{1}{i^2} < \infty.$$

This enables the application of Theorem W.1 on the sequence $\{\log Z_i(\boldsymbol{\eta},\boldsymbol{\eta}_0)\}_{i\in\mathbb{N}}$, to obtain

$$\frac{1}{d}\sum_{i=1}^{d} \log Z_i(\boldsymbol{\eta},\boldsymbol{\eta}_0) - \frac{1}{d}\sum_{i=1}^{d} \mathsf{E}_{\boldsymbol{\eta}_0}(\log Z_i(\boldsymbol{\eta},\boldsymbol{\eta}_0)) \overset{\mathsf{P}_{\boldsymbol{\eta}_0}-\text{a.s.}}{\longrightarrow} 0, \quad d \to \infty.$$

It follows from the last step and (CS3′) that there is a constant $c(\boldsymbol{\eta}) < 0$ so that (W3) holds.

Now we can prove Lemma 1.

Proof of Lemma 1. This proof is based on arguments by Chang and Stout (1991, Lemma 3.1).

For each $i \in \mathbb{N}$, we define the map $H_i : \Theta^2 \to \mathbb{R}_{\geq 0}$ by

$$(\boldsymbol{\eta},\boldsymbol{\eta}') \mapsto \left|\log\left(\frac{P_i(\boldsymbol{\eta})}{P_i(\boldsymbol{\eta}')}\right)\right| + \left|\log\left(\frac{1-P_i(\boldsymbol{\eta})}{1-P_i(\boldsymbol{\eta}')}\right)\right| =: H_i(\boldsymbol{\eta},\boldsymbol{\eta}').$$

Since the image of P_i does not include $\{0,1\}$, H_i is continuous. Moreover, for any $\boldsymbol{\eta}' \in \Theta$ and any $\delta > 0$ such that $\bar{B}_\delta(\boldsymbol{\eta}') \subset \Theta$, the map

$$\bar{B}_\delta(\boldsymbol{\eta}') \to \mathbb{R}_{\geq 0}, \quad \boldsymbol{\eta} \mapsto H_i(\boldsymbol{\eta},\boldsymbol{\eta}')$$

is continuous on the compact set $\bar{B}_\delta(\boldsymbol{\eta}')$ and thus has a maximum value, where $\bar{B}$ denotes the closure of the set B. We denote this maximum value by

$$\hat{H}_i(\delta,\boldsymbol{\eta}') := \max_{\boldsymbol{\eta}\in\bar{B}_\delta(\boldsymbol{\eta}')} H_i(\boldsymbol{\eta},\boldsymbol{\eta}').$$

In the following we assume, that $\delta > 0$ is sufficiently small so that $\bar{B}_\delta(\boldsymbol{\eta}') \subset \Theta$ for the selected value $\boldsymbol{\eta}' \in \Theta$. Letting $\delta \to 0$ yields $\bar{B}_\delta(\boldsymbol{\eta}') \to \{\boldsymbol{\eta}'\}$ and therefore, $\lim_{\delta\to 0} \hat{H}_i(\delta, \boldsymbol{\eta}') = 0$ for each $i \in \mathbb{N}$ and $\boldsymbol{\eta}' \in \Theta$. Since $Y_i \in \{0, 1\}$, we get from the triangle inequality

$$\begin{aligned}
|\log Z_i(\boldsymbol{\eta}, \boldsymbol{\eta}')| &= \left|\log\left(P_i(\boldsymbol{\eta})^{Y_i}(1 - P_i(\boldsymbol{\eta})^{1-Y_i}\right) - \log\left(P_i(\boldsymbol{\eta}')^{Y_i}(1 - P_i(\boldsymbol{\eta}')^{1-Y_i}\right)\right| \\
&= \left|Y_i \log\left(\frac{P_i(\boldsymbol{\eta})}{P_i(\boldsymbol{\eta}')}\right) + (1 - Y_i)\log\left(\frac{1 - P_i(\boldsymbol{\eta})}{1 - P_i(\boldsymbol{\eta}')}\right)\right| \\
&\leq \left|\log\left(\frac{P_i(\boldsymbol{\eta})}{P_i(\boldsymbol{\eta}')}\right)\right| + \left|\log\left(\frac{1 - P_i(\boldsymbol{\eta})}{1 - P_i(\boldsymbol{\eta}')}\right)\right| = H_i(\boldsymbol{\eta}, \boldsymbol{\eta}') \leq \hat{H}_i(\delta, \boldsymbol{\eta}'), \qquad \text{(W4)}
\end{aligned}$$

for $\delta \geq \|\boldsymbol{\eta} - \boldsymbol{\eta}'\|$. For any fixed $\boldsymbol{\eta}' \in \Theta$ we get

$$\begin{aligned}
\hat{H}_i(\delta, \boldsymbol{\eta}') &= \max_{\boldsymbol{\eta}\in\bar{B}_\delta(\boldsymbol{\eta}')} \left\{\left|\log\left(\frac{P_i(\boldsymbol{\eta})}{P_i(\boldsymbol{\eta}')}\right)\right| + \left|\log\left(\frac{1 - P_i(\boldsymbol{\eta})}{1 - P_i(\boldsymbol{\eta}')}\right)\right|\right\} \\
&\leq \max_{\boldsymbol{\eta}\in\bar{B}_\delta(\boldsymbol{\eta}')} \left\{\left|\log\left(\frac{P_i(\boldsymbol{\eta})}{P_i(\boldsymbol{\eta}')}\right)\right|\right\} + \max_{\boldsymbol{\eta}\in\bar{B}_\delta(\boldsymbol{\eta}')} \left\{\left|\log\left(\frac{1 - P_i(\boldsymbol{\eta})}{1 - P_i(\boldsymbol{\eta}')}\right)\right|\right\}.
\end{aligned}$$

Applying the multivariate mean value theorem to $\log(\,\cdot\,)$ we get

$$\begin{aligned}
\max_{\boldsymbol{\eta}\in\bar{B}_\delta(\boldsymbol{\eta}')} \left\{\left|\log\left(\frac{P_i(\boldsymbol{\eta})}{P_i(\boldsymbol{\eta}')}\right)\right|\right\} &= \max_{\boldsymbol{\eta}\in\bar{B}_\delta(\boldsymbol{\eta}')} \left\{\left|\log\left(P_i(\boldsymbol{\eta})\right) - \log\left(P_i(\boldsymbol{\eta}')\right)\right|\right\} \\
&= \max_{\boldsymbol{\eta}\in\bar{B}_\delta(\boldsymbol{\eta}')} \left\{\frac{1}{\xi(P_i(\boldsymbol{\eta}), P_i(\boldsymbol{\eta}'))}\left|P_i(\boldsymbol{\eta}) - P_i(\boldsymbol{\eta}')\right|\right\}, \qquad \text{(W5)}
\end{aligned}$$

where $\xi(P_i(\boldsymbol{\eta}), P_i(\boldsymbol{\eta}'))$ is a point between $P_i(\boldsymbol{\eta})$ and $P_i(\boldsymbol{\eta}')$. Let $\zeta_0(K)$ and $\zeta_1(K)$ be given for each compact $K \subset \Theta$ as in (CS2′). Additionally, due to Lemma W.1, for each compact and convex set $K \subseteq \Theta$ there is a $\zeta_3(K) > 0$ such that

$$\left|P_i(\boldsymbol{\eta}) - P_i(\boldsymbol{\eta}')\right| \leq \zeta_3(K)\|\boldsymbol{\eta} - \boldsymbol{\eta}'\|, \qquad \text{(W6)}$$

for all $i \in \mathbb{N}$. Combining (W5), the analogously derived version for $1 - P_i(\boldsymbol{\eta})$ instead of $P_i(\boldsymbol{\eta})$, (W6) and equation (20) in condition (CS2'), we obtain

$$\begin{aligned}
&\max_{\boldsymbol{\eta}\in\bar{B}_\delta(\boldsymbol{\eta}')} \left\{\left|\log\left(\frac{P_i(\boldsymbol{\eta})}{P_i(\boldsymbol{\eta}')}\right)\right|\right\} + \max_{\boldsymbol{\eta}\in\bar{B}_\delta(\boldsymbol{\eta}')} \left\{\left|\log\left(\frac{1 - P_i(\boldsymbol{\eta})}{1 - P_i(\boldsymbol{\eta}')}\right)\right|\right\} \\
&\qquad \leq \left(\frac{\zeta_3(K)}{\zeta_0(K)} + \frac{\zeta_3(K)}{1 - \zeta_1(K)}\right)\delta =: C(K)\delta,
\end{aligned}$$

for all compact and convex $K \supseteq \bar{B}_\delta(\boldsymbol{\eta}')$. Therefore, for each $\varepsilon > 0$ there is a $\delta > 0$ so that $\frac{1}{d}\sum_{i=1}^d \hat{H}_i(\delta, \boldsymbol{\eta}') < \varepsilon$, for all $d \in \mathbb{N}$. This implies

$$\lim_{\delta\to 0}\lim_{d\to\infty} \frac{1}{d}\sum_{i=1}^{d} \hat{H}_i(\delta, \boldsymbol{\eta}') = 0. \qquad \text{(W7)}$$

Next, we will show that for any $\boldsymbol{\eta}_i \neq \boldsymbol{\eta}_0$ there is a sufficiently small $\delta_i > 0$ and a sufficiently large $c_i < 0$ such that

$$\lim_{d\to\infty} \mathsf{P}_{\boldsymbol{\eta}_0}\left(\sup_{\boldsymbol{\eta}\in\bar{B}_{\delta_i}(\boldsymbol{\eta}_i)} \frac{1}{d}\left(\ell^{(d)}(\boldsymbol{\eta}\,|\,\mathbf{Y}^{(d)}) - \ell^{(d)}(\boldsymbol{\eta}_0\,|\,\mathbf{Y}^{(d)})\right) < c_i < 0 \right) = 1. \tag{W8}$$

First, for any $\boldsymbol{\eta} \in \bar{B}_{\delta_i}(\boldsymbol{\eta}_i)$ equation (W4) implies

$$\begin{aligned}
\frac{1}{d}\left(\ell^{(d)}(\boldsymbol{\eta}\,|\,\mathbf{Y}^{(d)}) - \ell^{(d)}(\boldsymbol{\eta}_0\,|\,\mathbf{Y}^{(d)})\right) &\leq \frac{1}{d}\left(\ell^{(d)}(\boldsymbol{\eta}_i\,|\,\mathbf{Y}^{(d)}) - \ell^{(d)}(\boldsymbol{\eta}_0\,|\,\mathbf{Y}^{(d)})\right) \\
&\quad + \frac{1}{d}\left(\ell^{(d)}(\boldsymbol{\eta}\,|\,\mathbf{Y}^{(d)}) - \ell^{(d)}(\boldsymbol{\eta}_i\,|\,\mathbf{Y}^{(d)})\right) \\
&\leq \frac{1}{d}\left(\ell^{(d)}(\boldsymbol{\eta}_i\,|\,\mathbf{Y}^{(d)}) - \ell^{(d)}(\boldsymbol{\eta}_0\,|\,\mathbf{Y}^{(d)})\right) + \frac{1}{d}\sum_{i=1}^{d} \hat{H}_i(\delta, \boldsymbol{\eta}_i).
\end{aligned}$$

Since $\boldsymbol{\eta}_i \in \Theta \setminus \{\boldsymbol{\eta}_0\}$, for $\boldsymbol{\eta} = \boldsymbol{\eta}_i$ we get from Lemma W.2 that

$$\limsup_{d\to\infty} \frac{1}{d}\left(\ell^{(d)}(\boldsymbol{\eta}_i\,|\,\mathbf{Y}^{(d)}) - \ell^{(d)}(\boldsymbol{\eta}_0\,|\,\mathbf{Y}^{(d)})\right) \leq c(\boldsymbol{\eta}_i) < 0 \quad \mathsf{P}_{\boldsymbol{\eta}_0}\text{– almost surely.}$$

Equation (W7) implies, for all $\boldsymbol{\eta}' = \boldsymbol{\eta}_i$ and for each $\varepsilon > 0$, that there is a sufficiently small $\delta > 0$ such that $\limsup_{d\to\infty} \frac{1}{d}\sum_{i=1}^{d} \hat{H}_i(\delta, \boldsymbol{\eta}_i) < \varepsilon$. Therefore, there is a $\delta > 0$ sufficiently small and a negative number c_i, for example $c_i = \frac{c(\boldsymbol{\eta}_i)}{2}$ so that (W8) holds.

Equation (W8) still holds if we replace $\overline{B}_\delta(\boldsymbol{\eta}_i)$ by an arbitrary subset of $\overline{B}_\delta(\boldsymbol{\eta}_i)$. Especially, for all $\boldsymbol{\eta}_i \neq \boldsymbol{\eta}_0$, there exists a sufficiently small δ_i and a constant $c_i < 0$ such that for all compact sets $K_i \subset \overline{B}_{\delta_i}(\boldsymbol{\eta}_i)$

$$\lim_{d\to\infty} \mathsf{P}_{\boldsymbol{\eta}_0}\left(\sup_{\boldsymbol{\eta}\in K_i} \frac{1}{d}\left(\ell^{(d)}(\boldsymbol{\eta}\,|\,\mathbf{Y}^{(d)}) - \ell^{(d)}(\boldsymbol{\eta}_0\,|\,\mathbf{Y}^{(d)})\right) < c_i \right) = 1 \tag{W9}$$

holds.

Next, we carry out a case-by-case analysis. First, we assume that Θ is compact. Then for each $\delta > 0$ such that $\Theta\setminus B_\delta(\boldsymbol{\eta}_0) \neq \emptyset$, we define $\Theta_0 := \Theta\setminus B_\delta(\boldsymbol{\eta}_0)$, which is compact, too. For each $\delta' > 0$, $\bigcup_{\boldsymbol{\eta}\in\Theta_0} B_{\delta'}(\boldsymbol{\eta})$ is a covering of Θ_0 and since Θ_0 is compact, there are $\boldsymbol{\eta}_1, \ldots, \boldsymbol{\eta}_N \in \Theta_0$ for an $N \in \mathbb{N}$ such that $\Theta_0 \subset \bigcup_{j=1}^{N} B_{\delta'}(\boldsymbol{\eta}_j)$. Notice that also $\Theta_0 = \bigcup_{j=1}^{N} K_i$ holds, where $K_i := \bar{B}_{\delta'}(\boldsymbol{\eta}_j) \cap \Theta_0$, $i = 1, \ldots, N$. Particularly, for every K_i there is a compact and convex superset $\tilde{K}_i \supset K_i$ with $\tilde{K}_i \subset \Theta$ due to (CS1′[i]). Then, we can define the events

$$A_i^{(d)} := \left\{ \omega \in \Omega\colon \sup_{\boldsymbol{\eta}\in K_i} \frac{1}{d}\left(\ell^{(d)}(\boldsymbol{\eta}\,|\,\mathbf{Y}^{(d)}(\omega)) - \ell^{(d)}(\boldsymbol{\eta}_0\,|\,\mathbf{Y}^{(d)}(\omega))\right) < c_i \right\}, \tag{W10}$$

for some $c_1, \ldots, c_N < 0$. Equation (W9) implies that $\mathsf{P}(A_i^{(d)}) \to 1$ for $d \to \infty$ and

$$\lim_{d\to\infty} \mathsf{P}_{\boldsymbol{\eta}_0}\left(\bigcap_{i=1}^{N} A_i^{(d)}\right) = 1. \tag{W11}$$

Equation (W11) is still true, if we replace in (W10) the constant c_i by $k = \max\{c_1, \ldots, c_N\} < 0$. We hence get

$$\lim_{d\to\infty} \mathsf{P}_{\boldsymbol{\eta}_0}\left(\sup_{\boldsymbol{\eta}\in\Theta\setminus B_\delta(\boldsymbol{\eta}_0)} \frac{1}{d}\left(\ell^{(d)}(\boldsymbol{\eta}\,|\,\mathbf{Y}^{(d)}) - \ell^{(d)}(\boldsymbol{\eta}_0\,|\,\mathbf{Y}^{(d)})\right) < k\right) = 1. \tag{W12}$$

Now, we assume that Θ is unbounded. Then we can define the sequence $\{\Theta_j\}_{j\in\mathbb{N}_0}$ with $\bigcup_{j\in\mathbb{N}_0}\Theta_j = \Theta \setminus B_\delta(\boldsymbol{\eta}_0)$ by setting

$$\Theta_j = \big\{\boldsymbol{\eta}\in\Theta\colon \delta + j \le \|\boldsymbol{\eta} - \boldsymbol{\eta}_0\| \le \delta + j + 1\big\}, \qquad j \in \mathbb{N}_0.$$

On each Θ_j, $j \in \mathbb{N}_0$, we can apply the analysis of the first case with Θ_j instead of $\Theta \setminus B_\delta(\boldsymbol{\eta}_0)$ and $k_j := \sup_{\boldsymbol{\eta}\in\Theta_j} c(\boldsymbol{\eta})/2 < 0$ instead of $k < 0$. Letting $d \to \infty$ we get with probability tending to one

$$\begin{aligned}
&\sup_{\boldsymbol{\eta}\in\Theta\setminus B_\delta(\boldsymbol{\eta}_0)} \frac{1}{d}\left(\ell^{(d)}(\boldsymbol{\eta}\,|\,\mathbf{Y}^{(d)}) - \ell^{(d)}(\boldsymbol{\eta}_0\,|\,\mathbf{Y}^{(d)})\right)\\
&\quad= \sup_{j\in\mathbb{N}_0}\left(\sup_{\boldsymbol{\eta}\in\Theta_j} \frac{1}{d}\left(\ell^{(d)}(\boldsymbol{\eta}\,|\,\mathbf{Y}^{(d)}) - \ell^{(d)}(\boldsymbol{\eta}_0\,|\,\mathbf{Y}^{(d)})\right)\right)\\
&\quad\le \sup_{j\in\mathbb{N}_0} k_j \le \sup_{\boldsymbol{\eta}\in\Theta\setminus B_\delta(\boldsymbol{\eta}_0)} c(\boldsymbol{\eta})/2 =: 2\cdot k(\delta).
\end{aligned}$$

Since condition (CS3′) implies $\sup_{\boldsymbol{\eta}\in\Theta\setminus B_\delta(\boldsymbol{\eta}_0)} c(\boldsymbol{\eta})/2 < 0$, the proof is completed by setting $k(\delta) := \sup_{\boldsymbol{\eta}\in\Theta\setminus B_\delta(\boldsymbol{\eta}_0)} c(\boldsymbol{\eta})/3 < 0$.

The following lemma can be found in Witting and Müller-Funk (1995, "Hilfssatz" 6.7 part b), p. 173).[1] This lemma is needed to show that there exist measurable solutions of the likelihood equations and, in particular, the MLE is actually a random vector. Thus, equations that contain the MLE can be manipulated as for any random vector.

Lemma W.3. Consider a function $g(\cdot\,,\cdot)$ so that for each fixed $\boldsymbol{\eta}\in\Theta\subset\mathbb{R}^q$ the mapping $g(\cdot,\boldsymbol{\eta})\colon(\mathbb{R}^d,\mathcal{B}^d)\to(\mathbb{R}^d,\mathcal{B}^d)$ is measurable and for each fixed $\boldsymbol{x}\in\mathbb{R}^d$ the mapping

[1]The lemma is translated to English and adapted to our notation.

$g(\boldsymbol{x}, \cdot)\colon \Theta \to \mathbb{R}^d$ is continuous. Assume that Θ is compact or that there is a sequence of compact sets $\{U_i\}_{i\in\mathbb{N}}$ with $\Theta = \bigcup_{i\in\mathbb{N}} U_i$. Further, assume that there is a mapping $\vartheta\colon \mathbb{R}^d \to \Theta$ such that $\boldsymbol{x} \mapsto g(\boldsymbol{x}, \vartheta(\boldsymbol{x})) =: h(\boldsymbol{x})$ is measurable. Then there is a measurable mapping $\tilde{\vartheta}\colon (\mathbb{R}^d, \mathcal{B}^d) \to (\Theta, \mathcal{B}(\Theta))$ such that $h(\boldsymbol{x}) = g(\boldsymbol{x}, \tilde{\vartheta}(\boldsymbol{x}))$ for all $\boldsymbol{x} \in \mathbb{R}^d$.

Next, we prove the consistency of the MLE and the MAP (Theorem 5 (i) and (ii)), which is required to prove Lemmas 2 and 3 and, consequently, Thereom 5 (iii) and Theorem 6.

Proof of Theorem 5 (i) and (ii). We start with part (i). The proof of the consistency of the MLE is based on the proof of Corollary 3.1 of Chang and Stout (1991). Similar to showing the existence of the MLE in classic maximum likelihood theory (cf. Lehmann and Casella, 1998), we define the set

$$\begin{aligned} C_{d,\delta} :=& \{\mathbf{y} \in \{0,1\}^d \,|\, \ell^{(d)}(\boldsymbol{c}\,|\,\mathbf{y}) < \ell^{(d)}(\boldsymbol{\eta}_0\,|\,\mathbf{y}) \text{ for all } \boldsymbol{c} \in \partial B_\delta(\boldsymbol{\eta}_0)\} \\ =& \left\{\mathbf{y} \in \{0,1\}^d \,\middle|\, \frac{1}{d}\ell^{(d)}(\boldsymbol{c}\,|\,\mathbf{y}) < \frac{1}{d}\ell^{(d)}(\boldsymbol{\eta}_0\,|\,\mathbf{y}) \text{ for all } \boldsymbol{c} \in \partial B_\delta(\boldsymbol{\eta}_0)\right\}, \end{aligned}$$

for $d \in \mathbb{N}$ and $\delta > 0$ such that $B_\delta(\boldsymbol{\eta}_0) \subset \Theta$, where ∂B denotes the boundary of the set B. By the definition of $C_{d,\delta}$, at least at the point $\boldsymbol{\eta}_0 \in B_\delta(\boldsymbol{\eta}_0)$, the mapping $\ell^{(d)}(\cdot\,|\,\mathbf{y})$ takes a larger value in the interior of the open ball $B_\delta(\boldsymbol{\eta}_0)$ than on the boundary $\partial B_\delta(\boldsymbol{\eta}_0)$ for all $\mathbf{y} \in C_{d,\delta}$. So, for all $\mathbf{y} \in C_{d,\delta}$, $\ell^{(d)}(\cdot\,|\,\mathbf{y})$ has at least one local maximum in $B_\delta(\boldsymbol{\eta}_0)$. For all $\mathbf{y} \in C_{d,\delta}$, we denote by $M_{\mathbf{y}} \subset B_\delta(\boldsymbol{\eta}_0)$ the set of all local maximum points of $\ell^{(d)}(\cdot\,|\,\mathbf{y})\big|_{B_\delta(\boldsymbol{\eta}_0)}$. Since $\ell^{(d)}(\cdot\,|\,\mathbf{y})\big|_{B_\delta(\boldsymbol{\eta}_0)} \in C^2(B_\delta(\boldsymbol{\eta}_0), \mathbb{R})$ due to (CS2′), $\ell^{(d)}(\cdot\,|\,\mathbf{y})\big|_{B_\delta(\boldsymbol{\eta}_0)}$ is continuously differentiable and so for all $\mathbf{y} \in C_{d,\delta}$ each point $\boldsymbol{\eta}^* \in M_{\mathbf{y}}$ satisfies the likelihood equations, i.e. $\nabla\ell^{(d)}(\boldsymbol{\eta}^*\,|\,\mathbf{y}) = \mathbf{0}$. Notice that $\partial B_\delta(\boldsymbol{\eta}_0) \subset \Theta \setminus B_\delta(\boldsymbol{\eta}_0)$ for all $\delta > 0$ with $B_\delta(\boldsymbol{\eta}_0) \subset \Theta$. Lemma 1 now implies $\mathsf{P}_{\boldsymbol{\eta}_0}(\mathbf{Y}^{(d)} \in C_{d,\delta}) \overset{d\to\infty}{\longrightarrow} 1$ and thus

$$\lim_{d\to\infty} \mathsf{P}_{\boldsymbol{\eta}_0}\left(\frac{1}{d}\ell^{(d)}(\boldsymbol{\eta}\,|\,\mathbf{Y}^{(d)}) < \frac{1}{d}\ell^{(d)}(\boldsymbol{\eta}_0\,|\,\mathbf{Y}^{(d)})\right) = 1, \qquad \text{for all } \boldsymbol{\eta} \in \Theta \setminus B_\delta(\boldsymbol{\eta}_0).$$

Therefore, with probability tending to one for $d \to \infty$, at least one of the local maxima of $\ell^{(d)}(\cdot\,|\,\mathbf{y})$ in the interior of $B_\delta(\boldsymbol{\eta}_0)$ has to be a global maximum of $\ell^{(d)}(\cdot\,|\,\mathbf{y})$.

Next, we have to select a specific maximum point in a way that it is a measurable mapping $(\{0,1\}^d, \mathrm{Pow}(\{0,1\}^d)) \to (\Theta, \mathcal{B}(\Theta))$, where Pow denotes the power set, to define a sequence of random variables, which are statistics of the response variables.

We write $\Theta_\delta := \overline{B}_\delta(\boldsymbol{\eta}_0) \cap \Theta \subset \Theta$ for a restricted compact parameter space.[2] Notice that

$$\ell^{(d)}(\cdot \,|\, \mathbf{y})\big|_{B_\delta(\boldsymbol{\eta}_0)} \colon (\Theta_\delta, \mathcal{B}(\Theta_\delta)) \to (\mathbb{R}, \mathcal{B}^1)$$

is continuous for each fixed $\mathbf{y} \in \{0,1\}^d$ and

$$\ell^{(d)}(\boldsymbol{\eta} \,|\, \cdot) \colon (\{0,1\}^d, \mathrm{Pow}(\{0,1\}^d)) \to (\mathbb{R}, \mathcal{B}^1)$$

is measurable for each fixed $\boldsymbol{\eta} \in \Theta_\delta$. Then by the remark of Witting and Müller-Funk (1995, page 173)

$$(\{0,1\}^d, \mathrm{Pow}(\{0,1\}^d)) \to (\mathbb{R}, \mathcal{B}^1), \quad \mathbf{y} \mapsto \sup_{\boldsymbol{\eta} \in \Theta_\delta} \ell^{(d)}(\boldsymbol{\eta} \,|\, \mathbf{y})$$

is a measurable mapping. Further, since Θ_δ is compact, for each $\mathbf{y} \in \{0,1\}^d$ the mapping $\ell^{(d)}(\cdot \,|\, \mathbf{y})$ takes the maximum over Θ_δ and we can select an $\boldsymbol{\eta}^* \in \Theta_\delta$ such that

$$\sup_{\boldsymbol{\eta} \in \Theta_\delta} \ell^{(d)}(\boldsymbol{\eta} \,|\, \mathbf{y}) = \ell^{(d)}(\boldsymbol{\eta}^* \,|\, \mathbf{y}).$$

We may now apply Lemma W.3 to ensure the existence of a measurable mapping

$$\tilde{\boldsymbol{\eta}}_d \colon (\{0,1\}^d, \mathrm{Pow}(\{0,1\}^d)) \to (\Theta, \mathcal{B}(\Theta))$$

such that $\sup_{\boldsymbol{\eta} \in \Theta_\delta} \ell^{(d)}(\boldsymbol{\eta} \,|\, \mathbf{y}) = \ell^{(d)}(\tilde{\boldsymbol{\eta}}_d(\mathbf{y}) \,|\, \mathbf{y})$ for all $\mathbf{y} \in \{0,1\}^d$. This enables us to well define the sequence $\{\hat{\boldsymbol{\eta}}_d\}_{d \in \mathbb{N}}$ of MLEs by setting

$$\hat{\boldsymbol{\eta}}_d := \tilde{\boldsymbol{\eta}}_d(\mathbf{Y}^{(d)}) \tag{W13}$$

for each $d \in \mathbb{N}$. By the previous part,

$$\lim_{d \to \infty} \mathsf{P}_{\boldsymbol{\eta}_0}\left(\left\{ \nabla \ell^{(d)}(\hat{\boldsymbol{\eta}}_d \mid \mathbf{Y}^{(d)}) = \mathbf{0} \right\} \cap \left\{ \ell^{(d)}(\hat{\boldsymbol{\eta}}_d \,|\, \mathbf{y}) = \sup_{\boldsymbol{\eta} \in \Theta} \ell^{(d)}(\boldsymbol{\eta} \,|\, \mathbf{y}) \right\} \right) = 1,$$

i.e., for $d \to \infty$, this probability is tending to one so that $\hat{\boldsymbol{\eta}}_d$ is a local maximum in Θ_δ and a global maximum of $\ell^{(d)}(\,\cdot \mid \mathbf{Y}^{(d)})$ (and thus the maximum likelihood estimator (MLE)).

[2]If Θ is compact, we do not have to restrict Θ. Nevertheless, we do it for the simplicity of the formulation. In fact, by Witting and Müller-Funk (1995, page 173), the following argument is in principle true even if Θ is unbounded. But then the corresponding random variables can become infinte, which we want to avoid.

It remains to prove the consistency. We assume that $\hat{\boldsymbol{\eta}}_d$ is the (restricted) MLE for $Y_1, \dots, Y_d$, $d \in \mathbb{N}$, given in (W13). Since it is the maximum of $\ell^{(d)}(\cdot \,|\, \mathbf{Y}^{(d)})\big|_{B_\delta(\boldsymbol{\eta}_0)}$ we have

$$\ell^{(d)}(\hat{\boldsymbol{\eta}}_d \,|\, \mathbf{Y}^{(d)}) - \ell^{(d)}(\boldsymbol{\eta}_0 \,|\, \mathbf{Y}^{(d)}) = \log\left(\frac{P^{(d)}(\mathbf{Y}^{(d)} \,|\, \hat{\boldsymbol{\eta}}_d)}{P^{(d)}(\mathbf{Y}^{(d)} \,|\, \boldsymbol{\eta}_0)}\right) \geq 0. \tag{W14}$$

Therefore, it is sufficient to proof for all $\varepsilon > 0$ and $\delta > 0$ that there is an $N(\varepsilon, \delta) \in \mathbb{N}$ such that $\mathsf{P}_{\boldsymbol{\eta}_0}(\|\hat{\boldsymbol{\eta}}_d - \boldsymbol{\eta}_0\| < \delta) > 1 - \varepsilon$, for all $d > N(\varepsilon, \delta)$. Suppose $\hat{\boldsymbol{\eta}}_d$ is not consistent, then there exists $\varepsilon_0 > 0$ and $\delta_0 > 0$ such that for any $N \in \mathbb{N}$ there exists some $d > N$ satisfying $\mathsf{P}_{\boldsymbol{\eta}_0}(\|\hat{\boldsymbol{\eta}}_d - \boldsymbol{\eta}_0\| > \delta_0) > \varepsilon_0$. Therefore, we can obtain a subsequence $\{\hat{\boldsymbol{\eta}}_{d_i}\}_{i \in \mathbb{N}}$ so that $\mathsf{P}_{\boldsymbol{\eta}_0}(\|\hat{\boldsymbol{\eta}}_{d_i} - \boldsymbol{\eta}_0\| > \delta_0) > \varepsilon_0$, for all $i \in \mathbb{N}$. This especially implies

$$\varepsilon_0 \leq \limsup_{d \to \infty} \mathsf{P}_{\boldsymbol{\eta}_0}(\|\hat{\boldsymbol{\eta}}_d - \boldsymbol{\eta}_0\| > \delta_0) \leq \mathsf{P}_{\boldsymbol{\eta}_0}(A_0), \tag{W15}$$

with

$$A_0 := \bigcap_{d \in \mathbb{N}} \bigcup_{m \geq d} \left\{ \omega \in \Omega \,\middle|\, \|\hat{\boldsymbol{\eta}}_m(\omega) - \boldsymbol{\eta}_0\| > \delta_0 \right\},$$

since $\limsup_{d \to \infty} \mathsf{P}_{\boldsymbol{\eta}_0}(E_d) \leq \mathsf{P}_{\boldsymbol{\eta}_0}(\limsup_{d \to \infty} E_d)$ for all sequences of events $\{E_d\}_{d \in \mathbb{N}}$. For all $\omega \in A_0$ we get $\hat{\boldsymbol{\eta}}_d(\omega) \in \Theta \backslash B_{\delta_0}(\boldsymbol{\eta}_0)$ for an infinite number of $d \in \mathbb{N}$ and this implies

$$\sup_{\boldsymbol{\eta} \in \Theta \backslash B_{\delta_0}(\boldsymbol{\eta}_0)} \frac{1}{d} \left(\ell^{(d)}(\boldsymbol{\eta} \,|\, \mathbf{Y}^{(d)}) - \ell^{(d)}(\hat{\boldsymbol{\eta}}_d \,|\, \mathbf{Y}^{(d)}) \right) \geq 0$$

for infinitely many $d \in \mathbb{N}$. In particular, it holds

$$A_0 \subset \bigcap_{d \in \mathbb{N}} \bigcup_{m \geq d} \left\{ \omega \in \Omega \,\middle|\, \sup_{\boldsymbol{\eta} \in \Theta \backslash B_{\delta_0}(\boldsymbol{\eta}_0)} \frac{1}{m} \left(\ell^{(m)}(\boldsymbol{\eta} \,|\, \mathbf{Y}^{(m)}(\omega)) - \ell^{(m)}(\hat{\boldsymbol{\eta}}_m(\omega) \,|\, \mathbf{Y}^{(m)}(\omega)) \right) \geq 0 \right\}.$$

Now, (W14) and (W15) imply

$$\varepsilon_0 \leq \mathsf{P}_{\boldsymbol{\eta}_0}(A_1) \tag{W16}$$

for the event

$$A_1 := \bigcap_{d \in \mathbb{N}} \bigcup_{m \geq d} \left\{ \omega \in \Omega \,\middle|\, \sup_{\boldsymbol{\eta} \in \Theta \backslash B_{\delta_0}(\boldsymbol{\eta}_0)} \frac{1}{m} \left(\ell^{(m)}(\boldsymbol{\eta} \,|\, \mathbf{Y}^{(m)}(\omega)) - \ell^{(m)}(\boldsymbol{\eta}_0 \,|\, \mathbf{Y}^{(m)}(\omega)) \right) \geq 0 \right\}.$$

But, according to Lemma 1, there is a $c = c(\delta_0) < 0$ such that for the event

$$B := \bigcap_{d \in \mathbb{N}} \bigcup_{m \geq d} \left\{ \omega \in \Omega \,\middle|\, \sup_{\boldsymbol{\eta} \in \Theta \backslash B_{\delta_0}(\boldsymbol{\eta}_0)} \frac{1}{m} \left(\ell^{(m)}(\boldsymbol{\eta} \,|\, \mathbf{Y}^{(m)}(\omega)) - \ell^{(m)}(\boldsymbol{\eta}_0 \,|\, \mathbf{Y}^{(m)}(\omega)) \right) < c \right\}$$

we get

$$\begin{aligned}1 &= \lim_{d\to\infty} \mathsf{P}_{\boldsymbol{\eta}_0}\left(\sup_{\boldsymbol{\eta}\in\Theta\setminus B_{\delta_0}(\boldsymbol{\eta}_0)} \frac{1}{d}\left(\ell^{(d)}(\boldsymbol{\eta}\,|\,\mathbf{Y}^{(d)}) - \ell^{(d)}(\boldsymbol{\eta}_0\,|\,\mathbf{Y}^{(d)})\right) < c < 0\right)\\ &= \limsup_{d\to\infty} \mathsf{P}_{\boldsymbol{\eta}_0}\left(\sup_{\boldsymbol{\eta}\in\Theta\setminus B_{\delta_0}(\boldsymbol{\eta}_0)} \frac{1}{d}\left(\ell^{(d)}(\boldsymbol{\eta}\,|\,\mathbf{Y}^{(d)}) - \ell^{(d)}(\boldsymbol{\eta}_0\,|\,\mathbf{Y}^{(d)})\right) < c < 0\right)\\ &\leq \mathsf{P}_{\boldsymbol{\eta}_0}(B),\end{aligned} \tag{W17}$$

where we can replace "$\leq$" in the last step by "$=$". Notice that $A_1 \cap B = \emptyset$, since

$$0 \leq \frac{1}{d}\left(\ell^{(d)}(\boldsymbol{\eta}\,|\,\mathbf{Y}^{(d)}) - \ell^{(d)}(\boldsymbol{\eta}_0\,|\,\mathbf{Y}^{(d)})\right) \leq c < 0$$

is impossible. Combining (W16) and (W17) results in

$$1 \geq \mathsf{P}_{\boldsymbol{\eta}_0}(A_1 \cup B) = \mathsf{P}_{\boldsymbol{\eta}_0}(A_1) + \mathsf{P}_{\boldsymbol{\eta}_0}(B) \geq \varepsilon_0 + 1 > 1,$$

which is a contradiction and proves the consistency of the MLE.

For part (ii), we show that Lemma 1 is still valid if we replace ℓ with $\tilde{\ell}$. The remaining steps are identical to the proof of part (i), simply replacing ℓ by $\tilde{\ell}$, its maximum $\hat{\boldsymbol{\eta}}_d$ by $\tilde{\boldsymbol{\eta}}_d$ and equation (W14) by

$$\tilde{\ell}^{(d)}(\tilde{\boldsymbol{\eta}}_d\,|\,\mathbf{Y}^{(d)}) - \tilde{\ell}^{(d)}(\boldsymbol{\eta}_0\,|\,\mathbf{Y}^{(d)}) \geq 0.$$

For any $d \in \mathbb{N}$ and $\delta > 0$, we get

$$\begin{aligned}&\sup_{\boldsymbol{\eta}\in\Theta\setminus B_{\delta}(\boldsymbol{\eta}_0)} \frac{1}{d}\left(\tilde{\ell}^{(d)}(\boldsymbol{\eta}\,|\,\mathbf{Y}^{(d)}) - \tilde{\ell}^{(d)}(\boldsymbol{\eta}_0\,|\,\mathbf{Y}^{(d)})\right)\\ &\quad\leq \sup_{\boldsymbol{\eta}\in\Theta\setminus B_{\delta}(\boldsymbol{\eta}_0)} \left(\frac{1}{d}\left(\ell^{(d)}(\boldsymbol{\eta}\,|\,\mathbf{Y}^{(d)}) - \ell^{(d)}(\boldsymbol{\eta}_0\,|\,\mathbf{Y}^{(d)})\right)\right)\\ &\qquad+ \sup_{\boldsymbol{\eta}\in\Theta\setminus B_{\delta}(\boldsymbol{\eta}_0)} \left(\frac{1}{d}\left(\log\mathcal{W}(\boldsymbol{\eta}) - \log\mathcal{W}(\boldsymbol{\eta}_0)\right)\right)\\ &\quad\leq \sup_{\boldsymbol{\eta}\in\Theta\setminus B_{\delta}(\boldsymbol{\eta}_0)} \left(\frac{1}{d}\left(\ell^{(d)}(\boldsymbol{\eta}\,|\,\mathbf{Y}^{(d)}) - \ell^{(d)}(\boldsymbol{\eta}_0\,|\,\mathbf{Y}^{(d)})\right)\right) + \frac{1}{d}\left(\log(C_1) - \log\mathcal{W}(\boldsymbol{\eta}_0)\right).\end{aligned} \tag{W18}$$

Clearly, for any $C_2 \in (0,\infty)$ there is a $D \in \mathbb{N}$ such that

$$\frac{1}{d}\left(\log(C_1) - \log\mathcal{W}(\boldsymbol{\eta}_0)\right) = \frac{1}{d}\log\left(\frac{C_1}{\mathcal{W}(\boldsymbol{\eta}_0)}\right) < C_2, \tag{W19}$$

for all $d > D$. Thus, we can apply Lemma 1 and conclude from (W18) and (W19) with $C_2 := -\tilde{k}(\delta)$ for $\tilde{k}(\delta) := k(\delta)/2$ with $k(\delta)$ from Lemma 1 for all $\delta > 0$:

$$\lim_{d\to\infty} \mathsf{P}_{\boldsymbol{\eta}_0}\left(\sup_{\boldsymbol{\eta}\in\Theta\setminus B_\delta(\boldsymbol{\eta}_0)} \frac{1}{d}\left(\tilde{\ell}^{(d)}(\boldsymbol{\eta}\,|\,\mathbf{Y}^{(d)}) - \tilde{\ell}^{(d)}(\boldsymbol{\eta}_0\,|\,\mathbf{Y}^{(d)})\right) < \tilde{k}(\delta)\right) = 1.$$

Lemmas W.4 to W.6 that follow, are required for the proof of Lemma 2. We start with the equicontinuity of the sequence of Hessians of λ_i, $i \in \mathbb{N}$, and the sequence of item information matrices $\mathcal{I}_i$, $i \in \mathbb{N}$.

Lemma W.4. Suppose that the conditions (CS2′) and (CS4′) hold. If restricted to any convex and compact set $K \subseteq \Theta$, the two families of mappings $\{\nabla\nabla^\intercal\lambda_i\}_{i\in\mathbb{N}}$ and $\{\mathcal{I}_i\}_{i\in\mathbb{N}}$ are equicontinuous.

Proof. The proofs for $\{\nabla\nabla^\intercal\lambda_i\}_{i\in\mathbb{N}}$ and $\{\mathcal{I}_i\}_{i\in\mathbb{N}}$ can be formulated equivalently. Hence, let $F^{(i)}$ either be $\mathcal{I}_i$ or $\nabla\nabla^\intercal\lambda_i$ for all $i \in \mathbb{N}$ to simplify the notation. Further, we denote by $F_{jk}^{(i)}$ the (j,k)th component of $F^{(i)}$ for $j,k = 1,\ldots,q$ and all $i \in \mathbb{N}$. Conditions (CS2′) and (CS4′) imply that $\nabla F_{jk}^{(i)}(\boldsymbol{\eta})$ exists for all $j,k = 1,\ldots,q$, $i \in \mathbb{N}$ and $\boldsymbol{\eta} \in K$, and that there is a $C < \infty$ such that

$$\sup\left\{\left|\frac{\partial F_{jk}^{(i)}(\boldsymbol{\eta})}{\partial\eta_\ell}\right| : j,k,\ell = 1,\ldots,q,\ i \in \mathbb{N},\ \boldsymbol{\eta} \in K\right\} \le C. \tag{W20}$$

Due to the multivariate mean value theorem and the convexity of K, for all $\boldsymbol{\eta}_1, \boldsymbol{\eta}_2 \in K$, $j,k = 1,\ldots,q$ and $i \in \mathbb{N}$, there is a $c \in [0,1]$ so that

$$F_{jk}^{(i)}(\boldsymbol{\eta}_1) - F_{jk}^{(i)}(\boldsymbol{\eta}_2) = \nabla F_{jk}^{(i)}(\tilde{\boldsymbol{\eta}})^\intercal(\boldsymbol{\eta}_1 - \boldsymbol{\eta}_2),$$

where $\tilde{\boldsymbol{\eta}} = c\boldsymbol{\eta}_1 + (1-c)\boldsymbol{\eta}_2$. Then, by the Cauchy–Schwarz–inequality and (W20), we get

$$|F_{jk}^{(i)}(\boldsymbol{\eta}_1) - F_{jk}^{(i)}(\boldsymbol{\eta}_2)| \le \|\nabla F_{jk}^{(i)}(\tilde{\boldsymbol{\eta}})\| \cdot \|\boldsymbol{\eta}_1 - \boldsymbol{\eta}_2\| \le C\sqrt{q}\|\boldsymbol{\eta}_1 - \boldsymbol{\eta}_2\|. \tag{W21}$$

Notice that the right-hand side of (W21) is independent of i,j,k. We therefore obtain the following estimate for the maximum-norm of $F^{(i)}(\boldsymbol{\eta}_1) - F^{(i)}(\boldsymbol{\eta}_2)$

$$\|F^{(i)}(\boldsymbol{\eta}_1) - F^{(i)}(\boldsymbol{\eta}_2)\|_{\max} := \max_{1\le j,k\le q} |F_{jk}^{(i)}(\boldsymbol{\eta}_1) - F_{jk}^{(i)}(\boldsymbol{\eta}_2)| \le C\sqrt{q}\|\boldsymbol{\eta}_1 - \boldsymbol{\eta}_2\|,$$

for each $i \in \mathbb{N}$. The equivalence of all norms on $\mathbb{R}^{d\times d}$ implies that there is a $C' > 0$ so that

$$\|F^{(i)}(\boldsymbol{\eta}_1) - F^{(i)}(\boldsymbol{\eta}_2)\| \leq C'\|F^{(i)}(\boldsymbol{\eta}_1) - F^{(i)}(\boldsymbol{\eta}_2)\|_{\max} \leq C'C\sqrt{q}\|\boldsymbol{\eta}_1 - \boldsymbol{\eta}_2\|$$

for all $i \in \mathbb{N}$ and all $\boldsymbol{\eta}_1, \boldsymbol{\eta}_2 \in K$. The final step is done analogously to (W2) in the proof of the equicontinuity of the item response functions.

The next lemma is required for the approximation of the quadratic form of the Hessian of the log-likelihood by the quadratic form of the test information matrix, Q_d in Lemma 2. In particular, this is used for the final step in the proof of Lemma 2 where the valid quadratic approximation of the log-likelihood-ratio using the test information matrix is shown.

Lemma W.5. Let $\boldsymbol{A} \in \mathbb{R}^{q\times q}$ be symmetric and positive definite, $\boldsymbol{x} \in \mathbb{R}^q$ and $\boldsymbol{B} \in \mathbb{R}^{q\times q}$ for $q \in \mathbb{N}$. Then

$$|\boldsymbol{x}^\intercal \boldsymbol{A}\boldsymbol{B}\boldsymbol{x}| \leq \sqrt{\frac{\nu_{\max}(\boldsymbol{A})}{\nu_{\min}(\boldsymbol{A})}}\|\boldsymbol{B}\|\,\boldsymbol{x}^\intercal\boldsymbol{A}\boldsymbol{x},$$

where $\|\boldsymbol{B}\|$ denotes the spectral norm of the matrix $\boldsymbol{B}$.

Proof. We first observe that

$$\boldsymbol{x}^\intercal\boldsymbol{A}\boldsymbol{x} \geq \nu_{\min}(\boldsymbol{A})\|\boldsymbol{x}\|^2, \tag{W22}$$

due to the Courant-Fischer theorem.

Since $\boldsymbol{A}$ is symmetric and positive definite, we can define a scalar product and a norm by setting

$$\langle \boldsymbol{v}, \boldsymbol{w}\rangle_{\boldsymbol{A}} := \boldsymbol{v}^\intercal\boldsymbol{A}\boldsymbol{w}, \quad \text{and} \quad \|\boldsymbol{v}\|_{\boldsymbol{A}} := \sqrt{\langle \boldsymbol{v}, \boldsymbol{v}\rangle_{\boldsymbol{A}}}, \qquad \boldsymbol{v}, \boldsymbol{w} \in \mathbb{R}^q.$$

Additionally, we define the matrix norm

$$\|\boldsymbol{V}\|_{\boldsymbol{A}} := \sup_{\boldsymbol{v}\in\mathbb{R}^q\setminus\{\boldsymbol{0}\}} \frac{\|\boldsymbol{V}\boldsymbol{v}\|_{\boldsymbol{A}}}{\|\boldsymbol{v}\|_{\boldsymbol{A}}}, \qquad \boldsymbol{V} \in \mathbb{R}^{q\times q}. \tag{W23}$$

Since each symmetric and positive definite matrix has a unique symmetric and positive definite square root matrix, we get $\|\boldsymbol{V}\boldsymbol{v}\|_{\boldsymbol{A}} = \|\boldsymbol{A}^{1/2}\boldsymbol{V}\boldsymbol{v}\|$. The induced matrix norm is always compatible to the corresponding vector norm and hence we get from the sub-multiplicativity

$$\|\boldsymbol{V}\boldsymbol{v}\|_{\boldsymbol{A}} \leq \|\boldsymbol{A}^{1/2}\| \cdot \|\boldsymbol{V}\boldsymbol{v}\| = \nu_{\max}(\boldsymbol{A}^{1/2})\|\boldsymbol{V}\boldsymbol{v}\| = \nu_{\max}^{1/2}(\boldsymbol{A})\|\boldsymbol{V}\boldsymbol{v}\|. \tag{W24}$$

Applying (W22) and (W24) to (W23), we get an estimate for an upper bound

$$\|\boldsymbol{V}\|_{\boldsymbol{A}} \leq \sup_{\boldsymbol{v}\in\mathbb{R}^q\setminus\{\boldsymbol{0}\}} \frac{\nu_{\max}^{1/2}(\boldsymbol{A})\|\boldsymbol{V}\boldsymbol{v}\|}{\nu_{\min}^{1/2}(\boldsymbol{A})\|\boldsymbol{v}\|} = \sqrt{\frac{\nu_{\max}(\boldsymbol{A})}{\nu_{\min}(\boldsymbol{A})}}\|\boldsymbol{V}\|.$$

Finally, by the Cauchy-Schwarz's inequality and the sub-multiplicativity of compatible matrix norms we obtain

$$\langle \boldsymbol{x}, \boldsymbol{B}\boldsymbol{x}\rangle_{\boldsymbol{A}}^2 \leq \|\boldsymbol{x}\|_{\boldsymbol{A}}^2 \|\boldsymbol{B}\boldsymbol{x}\|_{\boldsymbol{A}}^2 \leq \|\boldsymbol{B}\|_{\boldsymbol{A}}^2 \|\boldsymbol{x}\|_{\boldsymbol{A}}^4 \leq \frac{\nu_{\max}(\boldsymbol{A})}{\nu_{\min}(\boldsymbol{A})}\|\boldsymbol{B}\|^2 \langle \boldsymbol{x}, \boldsymbol{x}\rangle_{\boldsymbol{A}}^2.$$

This is equivalent to

$$\Leftrightarrow |\langle \boldsymbol{x}, \boldsymbol{B}\boldsymbol{x}\rangle_{\boldsymbol{A}}| \leq \sqrt{\frac{\nu_{\max}(\boldsymbol{A})}{\nu_{\min}(\boldsymbol{A})}}\|\boldsymbol{B}\| \langle \boldsymbol{x}, \boldsymbol{x}\rangle_{\boldsymbol{A}},$$

which completes the proof.

Next, we prove that conditions (CS2′) and (CS5′) ensure the asymptotic regularity of $d^{-1}\mathcal{I}^{(d)}(\boldsymbol{\eta})$ at each $\boldsymbol{\eta} \in \Theta$, result that is later used to ensure the existence of $d\mathcal{I}^{(d)}(\hat{\boldsymbol{\eta}}_d)^{-1}$, where $\hat{\boldsymbol{\eta}}_d$ denotes the MLE of $\boldsymbol{\eta}$ based on $\mathbf{Y}^{(d)}$.

Lemma W.6. Suppose that conditions (CS2′) and (CS5′) hold. Then

$$\liminf_{d\to\infty} \nu_{\min}\left(\frac{1}{d}\mathcal{I}^{(d)}(\boldsymbol{\eta})\right) > 0$$

for all $\boldsymbol{\eta} \in \Theta$.

Proof. First, consider symmetric positive semi–definite matrices $\boldsymbol{A}_1, \boldsymbol{A}_2$, such that $\boldsymbol{A} := \boldsymbol{A}_1 + \boldsymbol{A}_2$ is positive definite. Assume that c_1, c_2 are positive constants and without loss of generality consider that $c_2 \geq c_1$. We set $\boldsymbol{B} := c_1\boldsymbol{A}_1 + c_2\boldsymbol{A}_2$. Then the Courant-Fischer theorem implies

$$\begin{aligned}
\nu_{\min}(\boldsymbol{B}) = \min_{\boldsymbol{x}:\ \|\boldsymbol{x}\|\neq 0} \frac{\boldsymbol{x}^\intercal\boldsymbol{B}\boldsymbol{x}}{\boldsymbol{x}^\intercal\boldsymbol{x}} &= \min_{\boldsymbol{x}:\ \|\boldsymbol{x}\|\neq 0} \frac{\boldsymbol{x}^\intercal\big(c_1\boldsymbol{A} + (c_2 - c_1)\boldsymbol{A}_2\big)\boldsymbol{x}}{\boldsymbol{x}^\intercal\boldsymbol{x}} \\
&= \min_{\boldsymbol{x}:\ \|\boldsymbol{x}\|\neq 0} \frac{c_1\boldsymbol{x}^\intercal\boldsymbol{A}\boldsymbol{x} + (c_2 - c_1)\boldsymbol{x}^\intercal\boldsymbol{A}_2\boldsymbol{x}}{\boldsymbol{x}^\intercal\boldsymbol{x}} \\
&\geq \min_{\boldsymbol{x}:\ \|\boldsymbol{x}\|\neq 0} \frac{c_1\boldsymbol{x}^\intercal\boldsymbol{A}\boldsymbol{x}}{\boldsymbol{x}^\intercal\boldsymbol{x}} = c_1\nu_{\min}(\boldsymbol{A}) > 0.
\end{aligned}$$

This generalizes directly to finite sums. Now consider an arbitrary $\boldsymbol{\eta} \in \Theta$. Condition (CS5′) implies that there is a $c > 0$ and $D \in \mathbb{N}$ such that

$$\nu_{\min}\left(\frac{1}{d}\sum_{i=1}^{d} \nabla\lambda_i(\boldsymbol{\eta})\nabla^\intercal\lambda_i(\boldsymbol{\eta})\right) > c$$

for all $d > D$. Additionally, condition (CS2′) implies that there is a $c' > 0$ so that

$$\inf_{i\in\mathbb{N}} P_i(\boldsymbol{\eta})\big(1 - P_i(\boldsymbol{\eta})\big) > c'.$$

Thus, we get $\nu_{\min}\left(\frac{1}{d}\mathcal{I}^{(d)}(\boldsymbol{\eta})\right) > c \cdot c'$ for all $d > D$ and consequently

$$\liminf_{d\to\infty} \nu_{\min}\left(\frac{1}{d}\mathcal{I}^{(d)}(\boldsymbol{\eta})\right) \geq c \cdot c' > 0.$$

We are now able to prove Lemmas 2 and 3, provided in the appendix of the paper.

Proof of Lemma 2. This proof is based on arguments by Chang and Stout (1991, Lemma 3.2).

We get the first part directly by using a second order Taylor expansion of $\ell^{(d)}(\cdot\,|\,\mathbf{Y}^{(d)})$ at $\hat{\boldsymbol{\eta}}_d$, since $\nabla\ell^{(d)}(\hat{\boldsymbol{\eta}}_d\,|\,\mathbf{Y}^{(d)}) = \mathbf{0}$. Theorem 5 (i) implies that for each $\varepsilon > 0$ and $\delta > 0$ there is an $N(\varepsilon,\delta) \in \mathbb{N}$ such that $\mathsf{P}_{\boldsymbol{\eta}_0}(\hat{\boldsymbol{\eta}}_d \in B_\delta(\boldsymbol{\eta}_0)) > 1 - \varepsilon$ for all $d > N(\varepsilon,\delta)$. Therefore, we assume without loss of generality that $\|\hat{\boldsymbol{\eta}}_d - \boldsymbol{\eta}_0\| \leq \delta$ for the discussed d and δ. Further, due to Lemma W.6, there is an $N \in \mathbb{N}$ so that

$$\inf_{\boldsymbol{\eta}\in B_\delta(\boldsymbol{\eta}_0)} \nu_{\min}\left(\frac{1}{d}\mathcal{I}_d(\boldsymbol{\eta})\right) > 0,$$

for all $d > N$. Notice that $\|A^{-1}\| = 1/\nu_{\min}(A)$ holds for the selected matrix norm (i.e. the spectral norm) for all $A \in \mathbb{R}^{q\times q}$ with $\det(A) \neq 0$. Therefore, we assume without restriction that $\frac{1}{d}\mathcal{I}_d(\hat{\boldsymbol{\eta}}_d)$ is regular and that there is a constant $C_0 > 0$ (which is independent of d) such that

$$\left\|\left(\frac{1}{d}\mathcal{I}_d(\hat{\boldsymbol{\eta}}_d)\right)^{-1}\right\| \leq \frac{1}{C_0}, \tag{W25}$$

for $d > N$.

Using the sub-multiplicative property of matrix norms and (W25), we get

$$\begin{aligned}
\|R_d(\boldsymbol{\eta})\| &= \left\|\left(\frac{1}{d}\mathcal{I}_d(\hat{\boldsymbol{\eta}}_d)\right)^{-1}\frac{1}{d}(\mathcal{I}_d(\hat{\boldsymbol{\eta}}_d) + \nabla\nabla^{\mathsf{T}}\ell^{(d)}(\boldsymbol{\eta}_d^*\,|\,\mathbf{Y}^{(d)}))\right\| \\
&\leq \left\|\left(\frac{1}{d}\mathcal{I}_d(\hat{\boldsymbol{\eta}}_d)\right)^{-1}\right\| \cdot \left\|\frac{1}{d}(\mathcal{I}_d(\hat{\boldsymbol{\eta}}_d) + \nabla\nabla^{\mathsf{T}}\ell^{(d)}(\boldsymbol{\eta}_d^*\,|\,\mathbf{Y}^{(d)}))\right\| \\
&\leq \frac{1}{C_0}\left\|\frac{1}{d}(\mathcal{I}_d(\hat{\boldsymbol{\eta}}_d) + \nabla\nabla^{\mathsf{T}}\ell^{(d)}(\boldsymbol{\eta}_d^*\,|\,\mathbf{Y}^{(d)}))\right\|.
\end{aligned}$$

We now study a decomposition of $\|\frac{1}{d}(\mathcal{I}_d(\hat{\boldsymbol{\eta}}_d) + H_d(\boldsymbol{\eta}_d^*))\|$ in order to prove (A4). Notice first that

$$\nabla\nabla^\intercal \ell^{(d)}(\boldsymbol{\eta}\,|\,\mathbf{Y}^{(d)}) = \sum_{i=1}^{d} \nabla\nabla^\intercal \lambda_i(\boldsymbol{\eta})(Y_i - P_i(\boldsymbol{\eta})) - \mathcal{I}^{(d)}(\boldsymbol{\eta})$$

for all $\boldsymbol{\eta} \in \Theta$. The triangle inequality next implies

$$\begin{aligned}
C_0\|R_d(\boldsymbol{\eta})\| \le & \left\|\frac{1}{d}\Big(\mathcal{I}_d(\hat{\boldsymbol{\eta}}_d) + \nabla\nabla^\intercal \ell^{(d)}(\boldsymbol{\eta}_d^*\,|\,\mathbf{Y}^{(d)})\Big)\right\| \\
= & \left\|\frac{1}{d}\sum_{i=1}^{d} \nabla\nabla^\intercal \lambda_i(\boldsymbol{\eta}_d^*)(Y_i - P_i(\boldsymbol{\eta}_d^*)) - \frac{1}{d}\mathcal{I}^{(d)}(\boldsymbol{\eta}_d^*) + \frac{1}{d}\mathcal{I}^{(d)}(\hat{\boldsymbol{\eta}}_d)\right\| \\
= & \Bigg\|\frac{1}{d}\sum_{i=1}^{d} \Big(\nabla\nabla^\intercal \lambda_i(\boldsymbol{\eta}_d^*) - \nabla\nabla^\intercal \lambda_i(\boldsymbol{\eta}_0) + \nabla\nabla^\intercal \lambda_i(\boldsymbol{\eta}_0)\Big) \\
& \quad \times \Big(Y_i - P_i(\boldsymbol{\eta}_d^*) - P_i(\boldsymbol{\eta}_0) + P_i(\boldsymbol{\eta}_0)\Big) - \frac{1}{d}\mathcal{I}^{(d)}(\boldsymbol{\eta}_d^*) + \frac{1}{d}\mathcal{I}^{(d)}(\hat{\boldsymbol{\eta}}_d)\Bigg\| \\
= & \Bigg\|\frac{1}{d}\sum_{i=1}^{d} \nabla\nabla^\intercal \lambda_i(\boldsymbol{\eta}_0)(Y_i - P_i(\boldsymbol{\eta}_0)) + \frac{1}{d}\sum_{i=1}^{d} \nabla\nabla^\intercal \lambda_i(\boldsymbol{\eta}_0)(P_i(\boldsymbol{\eta}_0) - P_i(\boldsymbol{\eta}_d^*)) \\
& + \frac{1}{d}\sum_{i=1}^{d} \big(\nabla\nabla^\intercal \lambda_i(\boldsymbol{\eta}_d^*) - \nabla\nabla^\intercal \lambda_i(\boldsymbol{\eta}_0)\big)(Y_i - P_i(\boldsymbol{\eta}_d^*)) + \frac{1}{d}\Big(-\mathcal{I}^{(d)}(\boldsymbol{\eta}_d^*) + \mathcal{I}^{(d)}(\hat{\boldsymbol{\eta}}_d)\Big)\Bigg\| \\
\le & \left\|\frac{1}{d}\sum_{i=1}^{d} \nabla\nabla^\intercal \lambda_i(\boldsymbol{\eta}_0)(Y_i - P_i(\boldsymbol{\eta}_0))\right\| + \frac{1}{d}\sum_{i=1}^{d} \left\|\nabla\nabla^\intercal \lambda_i(\boldsymbol{\eta}_0)(P_i(\boldsymbol{\eta}_0) - P_i(\boldsymbol{\eta}_d^*))\right\| \\
& + \frac{1}{d}\sum_{i=1}^{d} \left\|\big(\nabla\nabla^\intercal \lambda_i(\boldsymbol{\eta}_d^*) - \nabla\nabla^\intercal \lambda_i(\boldsymbol{\eta}_0)\big)(Y_i - P_i(\boldsymbol{\eta}_d^*))\right\| \qquad \text{(W26)} \\
& + \frac{1}{d}\sum_{i=1}^{d} \left\| - \mathcal{I}_i(\boldsymbol{\eta}_d^*) + \mathcal{I}_i(\hat{\boldsymbol{\eta}}_d)\right\|.
\end{aligned}$$

Since $Y_i \in \{0,1\}$, $P_i \in (0,1)$, $i \in \mathbb{N}$, and due to Lemma W.4, there are constants $C_1, C_2 > 0$ such that

$$\| - \mathcal{I}_i(\boldsymbol{\eta}_d^*) + \mathcal{I}_i(\hat{\boldsymbol{\eta}}_d)\| \le C_1\|\boldsymbol{\eta}_d^* - \hat{\boldsymbol{\eta}}_d\|, \tag{W27}$$

and

$$\begin{aligned}
\|(\nabla\nabla^\intercal \lambda_i(\boldsymbol{\eta}_d^*) - \nabla\nabla^\intercal \lambda_i(\boldsymbol{\eta}_0))(Y_i - P_i(\boldsymbol{\eta}_d^*))\| &\le \|\nabla\nabla^\intercal \lambda_i(\boldsymbol{\eta}_d^*) - \nabla\nabla^\intercal \lambda_i(\boldsymbol{\eta}_0)\| \\
&\le C_2\|\boldsymbol{\eta}_d^* - \boldsymbol{\eta}_0\|, \qquad \text{(W28)}
\end{aligned}$$

for $i \in \mathbb{N}$. Further, due to (CS2′), the norm-equivalence and Lemma W.1, there are constants

$C_4, C_5 > 0$ such that

$$\begin{aligned}\|\nabla\nabla^\intercal\lambda_i(\boldsymbol{\eta}_0)(P_i(\boldsymbol{\eta}_0)-P_i(\boldsymbol{\eta}_d^*))\| &\le C_4\|\nabla\nabla^\intercal\lambda_i(\boldsymbol{\eta}_0)(P_i(\boldsymbol{\eta}_0)-P_i(\boldsymbol{\eta}_d^*))\|_{\max}\\ &\le C_4\|C_3\mathbf{1}_{q\times q}(P_i(\boldsymbol{\eta}_0)-P_i(\boldsymbol{\eta}_d^*))\|_{\max}\\ &\le C_3C_4|P_i(\boldsymbol{\eta}_0)-P_i(\boldsymbol{\eta}_d^*)|\\ &\le C_3C_4C_5\|\boldsymbol{\eta}_0-\boldsymbol{\eta}_d^*\| \end{aligned}\tag{W29}$$

for all $i \in \mathbb{N}$ with the maximum-norm $\|\cdot\|_{\max}$, the $q\times q$ matrix matrix of ones $\mathbf{1}_{q\times q}$ and

$$C_3 := \sup_{(i,j,k,\boldsymbol{\eta})\in\mathbb{N}\times\{1,\ldots,q\}^2\times B_\delta(\boldsymbol{\eta}_0)}\left|\frac{\partial^2\lambda_i(\boldsymbol{\eta})}{\partial\eta_j\partial\eta_k}\right|.$$

Next, for each $(j,k)\in\{1,\ldots,q\}^2$ and $i\in\mathbb{N}$ we get

$$\mathsf{E}_{\boldsymbol{\eta}_0}\left(\frac{\partial^2\lambda_i(\boldsymbol{\eta}_0)}{\partial\eta_j\partial\eta_k}Y_i\right)=\frac{\partial^2\lambda_i(\boldsymbol{\eta}_0)}{\partial\eta_j\partial\eta_k}P_i(\boldsymbol{\eta}_0)$$

and

$$\mathsf{Var}_{\boldsymbol{\eta}_0}\left(\frac{\partial^2\lambda_i(\boldsymbol{\eta}_0)}{\partial\eta_j\partial\eta_k}Y_i\right)\le C_3^2\mathsf{Var}_{\boldsymbol{\eta}_0}(Y_i)=C_3^2P_i(\boldsymbol{\eta}_0)(1-P_i(\boldsymbol{\eta}_0))\le\frac{C_3^2}{4}.$$

Therefore,

$$\sum_{i=1}^{\infty}\frac{\mathsf{Var}\left(\frac{\partial^2\lambda_i(\boldsymbol{\eta}_0)}{\partial\eta_j\partial\eta_k}Y_i\right)}{i^2}\le\frac{C_3^2\pi^2}{24}<\infty.$$

By applying Theorem W.1, we obtain

$$\frac{1}{d}\sum_{i=1}^{d}\left(\frac{\partial^2\lambda_i(\boldsymbol{\eta}_0)}{\partial\eta_j\partial\eta_k}(Y_i-P_i(\boldsymbol{\eta}_0))\right)\overset{\mathsf{P}_{\boldsymbol{\eta}_0}\text{-a.s.}}{\longrightarrow}0.$$

Especially, each component of $\frac{1}{d}\sum_{i=1}^{d}\nabla\nabla^\intercal\lambda_i(\boldsymbol{\eta}_0)(Y_i-P_i(\boldsymbol{\eta}_0))$ is almost surely converging to zero and hence

$$\left\|\frac{1}{d}\sum_{i=1}^{d}\nabla\nabla^\intercal\lambda_i(\boldsymbol{\eta}_0)(Y_i-P_i(\boldsymbol{\eta}_0))\right\|\overset{\mathsf{P}_{\boldsymbol{\eta}_0}\text{-a.s.}}{\longrightarrow}0.\tag{W30}$$

Combining (W26) to (W30), it follows that

$$\begin{aligned}\|R_d(\boldsymbol{\eta})\| &\le \frac{o_{\mathsf{P}_{\boldsymbol{\eta}_0}}(1)+\frac{1}{d}\sum_{i=1}^{d}(C_2+C_3C_4C_5)\|\boldsymbol{\eta}_0-\boldsymbol{\eta}_d^*\|+\frac{1}{d}\sum_{i=1}^{d}C_1\|\boldsymbol{\eta}_d^*-\hat{\boldsymbol{\eta}}_d\|}{C_0}\\ &= \frac{C_2+C_3C_4C_5}{C_0}\|\boldsymbol{\eta}_0-\boldsymbol{\eta}_d^*\|+\frac{C_1}{C_0}\|\boldsymbol{\eta}_d^*-\boldsymbol{\eta}_0+\boldsymbol{\eta}_0-\hat{\boldsymbol{\eta}}_d\|+o_{\mathsf{P}_{\boldsymbol{\eta}_0}}(1)\\ &\le \frac{C_2+C_3C_4C_5}{C_0}\|\boldsymbol{\eta}_0-\boldsymbol{\eta}_d^*\|+\frac{C_1}{C_0}(\|\boldsymbol{\eta}_d^*-\boldsymbol{\eta}_0\|+\|\boldsymbol{\eta}_0-\hat{\boldsymbol{\eta}}_d\|)+o_{\mathsf{P}_{\boldsymbol{\eta}_0}}(1),\end{aligned}$$

where

$$\|\boldsymbol{\eta}_d^* - \boldsymbol{\eta}_0\| \leq \max(\|\hat{\boldsymbol{\eta}}_d - \boldsymbol{\eta}_0\|, \|\boldsymbol{\eta} - \boldsymbol{\eta}_0\|) \leq \|\hat{\boldsymbol{\eta}}_d - \boldsymbol{\eta}_0\| + \|\boldsymbol{\eta} - \boldsymbol{\eta}_0\|,$$

since $\boldsymbol{\eta}_d^* \in \{a\hat{\boldsymbol{\eta}}_d + (1-a)\boldsymbol{\eta} : a \in [0,1]\}$. For any $\varepsilon > 0$ and d sufficiently large, we can set

$$\delta = \frac{C_0}{2C_1 + C_2 + C_3C_4C_5}\frac{\varepsilon}{2}$$

to obtain the second part of Lemma 2.

Finally, we shall prove its third part. By assumption, $\mathcal{I}_d(\hat{\boldsymbol{\eta}}_d)$ is always symmetric and for $d > N$ positive definite. Now, let $d > N$ be fixed, then

$$-\frac{1}{2}(\boldsymbol{\eta} - \hat{\boldsymbol{\eta}}_d)^\intercal \mathcal{I}_d(\hat{\boldsymbol{\eta}}_d)(\mathrm{I}_q - R_d(\boldsymbol{\eta}))(\boldsymbol{\eta} - \hat{\boldsymbol{\eta}}_d) = Q_d(\boldsymbol{\eta}) + \frac{1}{2}(\boldsymbol{\eta} - \hat{\boldsymbol{\eta}}_d)^\intercal \mathcal{I}_d(\hat{\boldsymbol{\eta}}_d)R_d(\boldsymbol{\eta})(\boldsymbol{\eta} - \hat{\boldsymbol{\eta}}_d).$$

Therefore, it is sufficient to show that for each $\varepsilon > 0$, it exists a $\delta > 0$ such that

$$\lim_{d\to\infty} \mathsf{P}_{\boldsymbol{\eta}_0}\left(\left|(\boldsymbol{\eta} - \hat{\boldsymbol{\eta}}_d)^\intercal \mathcal{I}_d(\hat{\boldsymbol{\eta}}_d)R_d(\boldsymbol{\eta})(\boldsymbol{\eta} - \hat{\boldsymbol{\eta}}_d)\right| \leq -2\varepsilon Q_d(\boldsymbol{\eta})\right) = 1,$$

for all $\boldsymbol{\eta} \in B_\delta(\boldsymbol{\eta}_0)$. Using Lemma W.5 with $\boldsymbol{A} = \mathcal{I}_d(\hat{\boldsymbol{\eta}}_d)$, $\boldsymbol{B} = R_d(\boldsymbol{\eta})$ and $\boldsymbol{x} = \boldsymbol{\eta} - \hat{\boldsymbol{\eta}}_d$, we get

$$\left|\frac{1}{2}(\boldsymbol{\eta} - \hat{\boldsymbol{\eta}}_d)^\intercal \mathcal{I}_d(\hat{\boldsymbol{\eta}}_d)R_d(\boldsymbol{\eta})(\boldsymbol{\eta} - \hat{\boldsymbol{\eta}}_d)\right| \leq -\sqrt{\frac{\nu_{\max}(\mathcal{I}_d(\hat{\boldsymbol{\eta}}_d))}{\nu_{\min}(\mathcal{I}_d(\hat{\boldsymbol{\eta}}_d))}}\,\|R_d(\boldsymbol{\eta})\|\,Q_d(\boldsymbol{\eta}).$$

By assumption (CS5') and the consistency of the MLE $\hat{\boldsymbol{\eta}}_d$, there is a constant $C_1' > 0$ such that

$$\mathsf{P}_{\boldsymbol{\eta}_0}\left(\limsup_{d\to\infty}\sqrt{\frac{\nu_{\max}(\mathcal{I}_d(\hat{\boldsymbol{\eta}}_d))}{\nu_{\min}(\mathcal{I}_d(\hat{\boldsymbol{\eta}}_d))}} \leq C_1'\right) = \mathsf{P}_{\boldsymbol{\eta}_0}\left(\limsup_{d\to\infty}\sqrt{\frac{\nu_{\max}(1/d\cdot\mathcal{I}_d(\hat{\boldsymbol{\eta}}_d))}{\nu_{\min}(1/d\cdot\mathcal{I}_d(\hat{\boldsymbol{\eta}}_d))}} \leq C_1'\right) = 1.$$

We complete the proof by applying the second part with $\varepsilon = \varepsilon_1/C_1'$ for an arbitrary $\varepsilon_1 > 0$.

Proof of Lemma 3. This proof is partially based on arguments by Chang and Stout (1991, Theorem 3.1).

1. Note that

$$\begin{aligned}&\frac{\int_{\mathbb{R}^q\setminus B_\delta(\boldsymbol{\eta}_0)} f(\boldsymbol{\eta})P^{(d)}(\mathbf{Y}^{(d)} \mid \boldsymbol{\eta})\mathfrak{h}(\boldsymbol{\eta})\,\mathrm{d}\boldsymbol{\eta}}{P^{(d)}(\mathbf{Y}^{(d)} \mid \hat{\boldsymbol{\eta}}_d)\det(\widehat{\boldsymbol{\Sigma}}_d^{1/2})}\\ &\quad = \exp\left(\ell^{(d)}(\boldsymbol{\eta}_0 \,|\mathbf{Y}^{(d)}) - \ell^{(d)}(\hat{\boldsymbol{\eta}}_d \,|\mathbf{Y}^{(d)})\right)\frac{T_d}{\det\left(\widehat{\boldsymbol{\Sigma}}_d^{1/2}\right)},\end{aligned} \tag{W31}$$

where

$$T_d := \int_{\mathbb{R}^q\setminus B_\delta(\boldsymbol{\eta}_0)} f(\boldsymbol{\eta})\exp\left(\ell^{(d)}(\boldsymbol{\eta}\,|\mathbf{Y}^{(d)}) - \ell^{(d)}(\boldsymbol{\eta}_0\,|\mathbf{Y}^{(d)})\right)\mathfrak{h}(\boldsymbol{\eta})\,\mathrm{d}\boldsymbol{\eta}. \tag{W32}$$

Since $\hat{\boldsymbol{\eta}}_d$ is a maximum of $\ell^{(d)}(\,\cdot\,|\mathbf{Y}^{(d)})$, it always holds

$$\exp\left(\ell^{(d)}(\boldsymbol{\eta}_0\,|\mathbf{Y}^{(d)}) - \ell^{(d)}(\hat{\boldsymbol{\eta}}_d\,|\mathbf{Y}^{(d)})\right) \leq 1$$

and therefore

$$\left|\frac{\int_{\mathbb{R}^q\setminus B_\delta(\boldsymbol{\eta}_0)} f(\boldsymbol{\eta}) P^{(d)}(\mathbf{Y}^{(d)} \mid \boldsymbol{\eta})\mathfrak{h}(\boldsymbol{\eta})\,\mathrm{d}\boldsymbol{\eta}}{P^{(d)}(\mathbf{Y}^{(d)} \mid \hat{\boldsymbol{\eta}}_d)\det\left(\widehat{\boldsymbol{\Sigma}}_d^{1/2}\right)}\right| \leq \left|\frac{T_d}{\det\left(\widehat{\boldsymbol{\Sigma}}_d^{1/2}\right)}\right|.$$

Condition (CS2') implies that all eigenvalues $\nu_i(d^{-1}\mathcal{I}^{(d)}(\boldsymbol{\eta}))$, $i = 1, \ldots, q$, of $d^{-1}\mathcal{I}^{(d)}(\boldsymbol{\eta})$ are bounded for all $\boldsymbol{\eta}$ in an arbitrary compact subset of Θ and all $d \in \mathbb{N}$. Utilizing this fact and by the consistency of $\hat{\boldsymbol{\eta}}_d$ for $d \to \infty$, we get

$$\begin{aligned}\det\left(\widehat{\boldsymbol{\Sigma}}_d^{1/2}\right)^{-1} = \sqrt{\det\left(\widehat{\boldsymbol{\Sigma}}_d^{-1}\right)} &= \sqrt{d^q \det\left(\frac{1}{d}\mathcal{I}^{(d)}(\hat{\boldsymbol{\eta}}_d)\right)} \\ &= d^{q/2}\sqrt{\prod_{i=1}^{q}\nu_i\left(\frac{1}{d}\mathcal{I}^{(d)}(\hat{\boldsymbol{\eta}}_d)\right)} = \mathcal{O}_{\mathsf{P}_{\eta_0}}\left(d^{q/2}\right). \qquad \text{(W33)}\end{aligned}$$

First, suppose that $\mathcal{H}$ is improper in a way that the posterior is proper and that equation (28) of the paper's discussion is satisfied. Further suppose that there is a constant $C_f > 0$ in such a way that $|f(\boldsymbol{\eta})| < C_f$ for all $\boldsymbol{\eta} \in \Theta$, i.e. f is bounded by a constant in absolute value. Then we get from the definition of T_d in (W32) and from (28)

$$|T_d| \leq C_f \frac{\int_{\mathbb{R}^q\setminus B_\delta(\boldsymbol{\eta}_0)} P^{(d)}(\mathbf{Y}^{(d)} \mid \boldsymbol{\eta})\mathfrak{h}(\boldsymbol{\eta})\,\mathrm{d}\boldsymbol{\eta}}{P^{(d)}(\mathbf{Y}^{(d)} \mid \boldsymbol{\eta}_0)} = o_{\mathsf{P}_{\eta_0}}\left(d^{-q/2}\right). \qquad \text{(W34)}$$

Combining (W33) and (W34)

directly implies

$$\left|\frac{T_d}{\det\left(\widehat{\boldsymbol{\Sigma}}_d^{1/2}\right)}\right| = o_{\mathsf{P}_{\eta_0}}(1).$$

Otherwise, one of the following cases holds by the conditions of Lemma 3(1.):

(i) f is bounded by a constant in absolute value and $\mathcal{H}$ is proper,

(ii) f is $\mathcal{H}$-integrable and $\mathcal{H}$ is proper,

(iii) f is $\mathcal{H}$-integrable and $\mathcal{H}$ is improper.

Each of these cases results in the fact that f is $\mathcal{H}$-integrable and hence $\mathsf{E}(|f \circ \boldsymbol{\eta}|) < \infty$. Lemma 1 implies that there is a $c(\delta) < 0$ such that

$$\lim_{d\to\infty} \mathsf{P}_{\boldsymbol{\eta}_0}\left(\sup_{\boldsymbol{\eta}\in\Theta\setminus B_\delta(\boldsymbol{\eta}_0)} \frac{1}{d}\left(\ell^{(d)}(\boldsymbol{\eta}\,|\mathbf{Y}^{(d)}) - \ell^{(d)}(\boldsymbol{\eta}_0\,|\mathbf{Y}^{(d)})\right) < c(\delta)\right) = 1. \tag{W35}$$

Further,

$$\begin{aligned}\left|\int_{\mathbb{R}^q\setminus B_\delta(\boldsymbol{\eta}_0)} f(\boldsymbol{\eta})\exp(dc(\delta))\mathfrak{h}(\boldsymbol{\eta})\,\mathrm{d}\boldsymbol{\eta}\right| &= \exp(dc(\delta))\left|\int_{\mathbb{R}^q\setminus B_\delta(\boldsymbol{\eta}_0)} f(\boldsymbol{\eta})\mathfrak{h}(\boldsymbol{\eta})\,\mathrm{d}\boldsymbol{\eta}\right| \\ &\le \exp(dc(\delta))\int_{\mathbb{R}^q\setminus B_\delta(\boldsymbol{\eta}_0)} \left|f(\boldsymbol{\eta})\right|\mathfrak{h}(\boldsymbol{\eta})\,\mathrm{d}\boldsymbol{\eta} \\ &\le \exp(dc(\delta))\mathsf{E}(|f\circ\boldsymbol{\eta}|). \end{aligned} \tag{W36}$$

Combining (W35) and (W36) results in

$$\lim_{d\to\infty} \mathsf{P}_{\boldsymbol{\eta}_0}\left(|T_d| \le \exp(dc(\delta))\mathsf{E}(|f\circ\boldsymbol{\eta}|)\right) = 1.$$

The facts that $d \mapsto \exp(dc(\delta))$ is decreasing faster than any polynomial and that $\det\left(\widehat{\boldsymbol{\Sigma}}_d^{1/2}\right)^{-1}$ grows in polynomial order, due to (W33), imply

$$\frac{\mathsf{E}(|f\circ\boldsymbol{\eta}|)\exp(dc(\delta))}{\det\left(\widehat{\boldsymbol{\Sigma}}_d^{1/2}\right)} \xrightarrow{\mathsf{P}_{\boldsymbol{\eta}_0}} 0,$$

which completes the proof of the first part.

2. Let $B \in \mathcal{B}^q$ be an arbitrary bounded Borel set and define for $\delta > 0$ and $d \in \mathbb{N}$ the set

$$M_{\delta,d} := B_\delta(\boldsymbol{\eta}_0) \cap G_d(B)$$

and the integral

$$V_d := \int_{M_{\delta,d}} P^{(d)}(\mathbf{Y}^{(d)}\,|\,\boldsymbol{\eta})\mathfrak{h}(\boldsymbol{\eta})\,\mathrm{d}\boldsymbol{\eta}.$$

By the definition of $R_d(\boldsymbol{\eta})$ in Lemma 2, we get

$$\begin{aligned}&\frac{V_d}{P^{(d)}(\mathbf{Y}^{(d)}\,|\,\hat{\boldsymbol{\eta}}_d)\det\left(\widehat{\boldsymbol{\Sigma}}_d^{1/2}\right)} \\ &= \frac{\mathfrak{h}(\boldsymbol{\eta}_0)}{\det\left(\widehat{\boldsymbol{\Sigma}}_d^{1/2}\right)}\int_{M_{\delta,d}} \frac{\mathfrak{h}(\boldsymbol{\eta})}{\mathfrak{h}(\boldsymbol{\eta}_0)}\exp\left(-\frac{1}{2}(\boldsymbol{\eta}-\hat{\boldsymbol{\eta}}_d)^\intercal\mathcal{I}_d(\hat{\boldsymbol{\eta}}_d)(\mathrm{I}-R_d(\boldsymbol{\eta}))(\boldsymbol{\eta}-\hat{\boldsymbol{\eta}}_d)\right)\mathrm{d}\boldsymbol{\eta}.\end{aligned} \tag{W37}$$

From (CS1′), i.e. the continuity of $\mathfrak{h}$ and $\mathfrak{h}(\boldsymbol{\eta}_0) > 0$, follows that for every $\varepsilon_1 > 0$, there is a $\delta_1 > 0$ such that

$$1-\varepsilon_1 \le \inf_{\boldsymbol{\eta}\in B_{\delta_1}(\boldsymbol{\eta}_0)} \frac{\mathfrak{h}(\boldsymbol{\eta})}{\mathfrak{h}(\boldsymbol{\eta}_0)} \le \inf_{\boldsymbol{\eta}\in M_{\delta_1,d}} \frac{\mathfrak{h}(\boldsymbol{\eta})}{\mathfrak{h}(\boldsymbol{\eta}_0)} \le \sup_{\boldsymbol{\eta}\in M_{\delta_1,d}} \frac{\mathfrak{h}(\boldsymbol{\eta})}{\mathfrak{h}(\boldsymbol{\eta}_0)} \le \sup_{\boldsymbol{\eta}\in B_{\delta_1}(\boldsymbol{\eta}_0)} \frac{\mathfrak{h}(\boldsymbol{\eta})}{\mathfrak{h}(\boldsymbol{\eta}_0)} \le 1+\varepsilon_1. \tag{W38}$$

Furthermore, for any $\varepsilon_2 > 0$ and appropriate $\delta_2 = \delta_2(\varepsilon_2) > 0$, we get from Lemma 2 that

$$\begin{aligned}&(1 - o_{\mathsf{P}_{\eta_0}}(1)) \int_{M_{\delta_2,d}} \frac{\mathfrak{h}(\boldsymbol{\eta})}{\mathfrak{h}(\boldsymbol{\eta}_0)} \exp\left(-\frac{1+\varepsilon_2}{2}(\boldsymbol{\eta} - \hat{\boldsymbol{\eta}}_d)^\intercal \mathcal{I}_d(\hat{\boldsymbol{\eta}}_d)(\boldsymbol{\eta} - \hat{\boldsymbol{\eta}}_d)\right) \mathrm{d}\boldsymbol{\eta} \\ &\le \int_{M_{\delta_2,d}} \frac{\mathfrak{h}(\boldsymbol{\eta})}{\mathfrak{h}(\boldsymbol{\eta}_0)} \exp\left(-\frac{1}{2}(\boldsymbol{\eta} - \hat{\boldsymbol{\eta}}_d)^\intercal \mathcal{I}_d(\hat{\boldsymbol{\eta}}_d)(\mathrm{I} - R_d(\boldsymbol{\eta}_d^*))(\boldsymbol{\eta} - \hat{\boldsymbol{\eta}}_d)\right) \mathrm{d}\boldsymbol{\eta} \\ &\le (1 + o_{\mathsf{P}_{\eta_0}}(1)) \int_{M_{\delta_2,d}} \frac{\mathfrak{h}(\boldsymbol{\eta})}{\mathfrak{h}(\boldsymbol{\eta}_0)} \exp\left(-\frac{1-\varepsilon_2}{2}(\boldsymbol{\eta} - \hat{\boldsymbol{\eta}}_d)^\intercal \mathcal{I}_d(\hat{\boldsymbol{\eta}}_d)(\boldsymbol{\eta} - \hat{\boldsymbol{\eta}}_d)\right) \mathrm{d}\boldsymbol{\eta}.\end{aligned} \tag{W39}$$

Next, the transformation theorem, (W38) and (W39) imply

$$\begin{aligned}&\tilde{\Phi}_q\left(\sqrt{1+\varepsilon_2}\,\mathcal{I}_d(\hat{\boldsymbol{\eta}}_d)^{1/2}\big(M_{\delta_2,d} - \hat{\boldsymbol{\eta}}_d\big)\right)\mathfrak{h}(\boldsymbol{\eta}_0)(2\pi)^{q/2}\frac{1-\varepsilon_1}{(1+\varepsilon_2)^{q/2}}\big(1 - o_{\mathsf{P}_{\eta_0}}(1)\big) \\ &\le \frac{V_d}{P^{(d)}(\mathbf{Y}^{(d)} \mid \hat{\boldsymbol{\eta}}_d) \det\big(\widehat{\boldsymbol{\Sigma}}_d^{1/2}\big)} \\ &\le \tilde{\Phi}_q\left(\sqrt{1-\varepsilon_2}\,\mathcal{I}_d(\hat{\boldsymbol{\eta}}_d)^{1/2}\big(M_{\delta_2,d} - \hat{\boldsymbol{\eta}}_d\big)\right)\mathfrak{h}(\boldsymbol{\eta}_0)(2\pi)^{q/2}\frac{1+\varepsilon_1}{(1-\varepsilon_2)^{q/2}}\big(1 + o_{\mathsf{P}_{\eta_0}}(1)\big).\end{aligned} \tag{W40}$$

In the case of (A6), it holds that $\lim_{d\to\infty} \mathsf{P}_{\eta_0}\big(G_d(B) = M_{\delta_2,d}\big) = 1$. Selecting ε_1 and ε_2 arbitrarily small leads to

$$\frac{\int_{G_d(B)} P^{(d)}(\mathbf{Y}^{(d)} \mid \boldsymbol{\eta})\mathfrak{h}(\boldsymbol{\eta})\,\mathrm{d}\boldsymbol{\eta}}{P^{(d)}(\mathbf{Y}^{(d)} \mid \hat{\boldsymbol{\eta}}_d)\det(\hat{\boldsymbol{\Sigma}}_d^{1/2})} = \tilde{\Phi}_q(\mathcal{I}_d(\hat{\boldsymbol{\eta}}_d)^{1/2}(G_d(B) - \hat{\boldsymbol{\eta}}_d))\mathfrak{h}(\boldsymbol{\eta}_0)(2\pi)^{q/2} + o_{\mathsf{P}_{\eta_0}}(1).$$

In the case of (A7), we get for each $\delta_2 < \delta$: $\lim_{d\to\infty} \mathsf{P}_{\eta_0}\big(B_{\delta_2}(\boldsymbol{\eta}_0) = M_{\delta_2,d}\big) = 1$. Condition (CS5′) implies that[3]

$$\sqrt{1+\varepsilon_2}\,\mathcal{I}_d(\hat{\boldsymbol{\eta}}_d)^{1/2}\big(B_{\delta_2}(\boldsymbol{\eta}_0) - \hat{\boldsymbol{\eta}}_d\big) \xrightarrow{\mathsf{P}_{\eta_0}} \mathbb{R}^d,$$

which leads to

$$\tilde{\Phi}_q\left(\sqrt{1+\varepsilon_2}\,\mathcal{I}_d(\hat{\boldsymbol{\eta}}_d)^{1/2}\big(B_{\delta_2}(\boldsymbol{\eta}_0) - \hat{\boldsymbol{\eta}}_d\big)\right) \xrightarrow{\mathsf{P}_{\eta_0}} 1.$$

Finally, the further valid selection of arbitrarily small $\varepsilon_1, \varepsilon_2 > 0$ in (W40) and the application of the Lemma 3 (1.) to $f = \mathbb{1}_{G_d(B)\setminus B_{\delta_2}(\eta_0)}$ completes the proof.

Lemma 3 (2.) and a utilization of the continuous mapping theorem with $\mathbb{R}\setminus\{0\} \to \mathbb{R}$, $x \mapsto x^{-1}$ directly imply Corollary 1 by setting $G_d(B) := \mathbb{R}^q$, $d \in \mathbb{N}$. Now we have accomplished all required preliminary steps in order to prove Theorem 5 (iii) and Theorem 7.

[3] This convergence is defined as follows: Let $\{A_d\}_{d\in\mathbb{N}}$ be a sequence of random sets with $A_d \subset \mathbb{R}^q$. Then we say $A_d \xrightarrow{\mathsf{P}_{\eta_0}} \mathbb{R}^q$, if for every $\varepsilon > 0$ and every compact $K \subset \mathbb{R}^q$, there exists an $N \in \mathbb{N}$ such that $\mathsf{P}_{\eta_0}\big(K \subset A_d\big) > 1 - \varepsilon$, for all $d > N$.

Proof of Theorem 5 (iii). This proof is partially based on arguments by Chang and Stout (1991, Theorems 3.1 and 3.3).

We start with the proof for the convergence in $\mathsf{P}_{\boldsymbol{\eta}_0}$ with restriction to bounded B. In the following step, this will then be extended to unbounded B and, finally, the convergence in P will be proved. Analogously to the proof of Lemma 2, we can assume without loss of generality that $\mathcal{I}^{(d)}(\hat{\boldsymbol{\eta}}_d)^{-1/2}$ exists. Set

$$G_d(B) := \left\{\widehat{\boldsymbol{\Sigma}}_d^{1/2}\boldsymbol{x} + \hat{\boldsymbol{\eta}}_d \,:\, \boldsymbol{x} \in B\right\} \equiv \mathcal{I}^{(d)}(\hat{\boldsymbol{\eta}}_d)^{-1/2}B + \hat{\boldsymbol{\eta}}_d, \qquad B \in \mathcal{B}^q,\ d \in \mathbb{N}. \tag{W41}$$

To show Theorem 5 (iii) for bounded B we utilize the reformulation

$$\begin{aligned} &\mathsf{P}\left(\mathcal{I}^{(d)}(\hat{\boldsymbol{\eta}}_d)^{1/2}\,(\boldsymbol{\eta} - \hat{\boldsymbol{\eta}}_d) \in B \,\middle|\, \mathbf{Y}^{(d)}\right) \\ &\quad = \frac{\int_{G_d(B)} P^{(d)}(\mathbf{Y}^{(d)} \mid \boldsymbol{\eta})\mathfrak{h}(\boldsymbol{\eta})\,\mathrm{d}\boldsymbol{\eta}}{P^{(d)}(\mathbf{Y}^{(d)} \mid \hat{\boldsymbol{\eta}}_d)\det\left(\widehat{\boldsymbol{\Sigma}}_d^{1/2}\right)}\left(\frac{P(\mathbf{Y}^{(d)})}{P^{(d)}(\mathbf{Y}^{(d)} \mid \hat{\boldsymbol{\eta}}_d)\det\left(\widehat{\boldsymbol{\Sigma}}_d^{1/2}\right)}\right)^{-1} \end{aligned} \tag{W42}$$

for each $d \in \mathbb{N}$. Since $\|\hat{\boldsymbol{\eta}}_d - \boldsymbol{\eta}_0\| \xrightarrow{\mathsf{P}_{\boldsymbol{\eta}_0}} 0$, from (CS5′) and the fact that $\|\boldsymbol{A}^{-1}\| = 1/\nu_{\min}(\boldsymbol{A})$ for any regular symmetric matrix $\boldsymbol{A}$, we get that

$$\left\|\widehat{\boldsymbol{\Sigma}}_d\right\| = \left\|\mathcal{I}^{(d)}(\hat{\boldsymbol{\eta}}_d)^{-1}\right\| = \frac{1}{d}\left\|\left(\frac{1}{d}\mathcal{I}^{(d)}(\hat{\boldsymbol{\eta}}_d)\right)^{-1}\right\| = \mathcal{O}_{\mathsf{P}_{\boldsymbol{\eta}_0}}\left(\frac{1}{d}\right).$$

This implies $\left\|\widehat{\boldsymbol{\Sigma}}_d\right\| \xrightarrow{\mathsf{P}_{\boldsymbol{\eta}_0}} 0$ and especially $\widehat{\boldsymbol{\Sigma}}_d \xrightarrow{\mathsf{P}_{\boldsymbol{\eta}_0}} \mathbf{0}$. Hence, for each $\delta_2 > 0$ and $\varepsilon > 0$, there is an $N \in \mathbb{N}$ such that $\mathsf{P}_{\boldsymbol{\eta}_0}(G_d(B) \subset B_{\delta_2}(\boldsymbol{\eta}_0)) > 1 - \varepsilon$ for all $d > N$, i.e. condition (A6) of Lemma 3 (2.) is satisfied, where G_d is defined in (W41). We can therefore apply Lemma 3 (2.) and get

$$\frac{\int_{G_d(B)} P^{(d)}(\mathbf{Y}^{(d)} \mid \boldsymbol{\eta})\mathfrak{h}(\boldsymbol{\eta})\,\mathrm{d}\boldsymbol{\eta}}{P^{(d)}(\mathbf{Y}^{(d)} \mid \hat{\boldsymbol{\eta}}_d)\det\left(\widehat{\boldsymbol{\Sigma}}_d^{1/2}\right)} \xrightarrow{\mathsf{P}_{\boldsymbol{\eta}_0}} \tilde{\Phi}_q(\mathcal{I}_d(\hat{\boldsymbol{\eta}}_d)^{1/2}(G_d(B) - \hat{\boldsymbol{\eta}}_d))\mathfrak{h}(\boldsymbol{\eta}_0)(2\pi)^{q/2}, \tag{W43}$$

for $d \to \infty$. Further, from the definition of G_d, we get

$$\mathcal{I}_d(\hat{\boldsymbol{\eta}}_d)^{1/2}\big(G_d(B) - \hat{\boldsymbol{\eta}}_d\big) = \mathcal{I}_d(\hat{\boldsymbol{\eta}}_d)^{1/2}\big(\mathcal{I}_d(\hat{\boldsymbol{\eta}}_d)^{-1/2}B + \hat{\boldsymbol{\eta}}_d - \hat{\boldsymbol{\eta}}_d\big) = B. \tag{W44}$$

Combining (W42), (W43), (W44) and Corollary 1 results in Theorem 5 (iii) for bounded B and convergence in $\mathsf{P}_{\boldsymbol{\eta}_0}$, i.e. (26) for bounded B.

Next, we show the case of unbounded $B \in \mathcal{B}^q$. In order to show this, we define the sequence of random probability measures $\{\Psi_d\}_{d\in\mathbb{N}}$ on $(\mathbb{R}^q, \mathcal{B}^q)$ by setting

$$\Psi_d(A) := \int_A |\det(\mathcal{I}_d(\hat{\boldsymbol{\eta}}_d)^{1/2})|\, h(G_d^{-1}(\boldsymbol{\eta}) \mid \mathbf{Y}^{(d)})\,\mathrm{d}\boldsymbol{\eta}, \qquad A \in \mathcal{B}^q, d \in \mathbb{N}.$$

Due to the transformation theorem,

$$\Psi_d(A) = \int_{G_d(A)} h(\boldsymbol{\eta} \,|\, \mathbf{Y}^{(d)}) \,\mathrm{d}\boldsymbol{\eta}$$

holds for all $d \in \mathbb{N}$ and $A \in \mathcal{B}^q$. Notice that Ψ_d is the posterior probability distribution of the affine transformation $G_d^{-1}(\boldsymbol{\eta})$ of $\boldsymbol{\eta}$ given $\mathbf{Y}^{(d)}$. So, $\Psi_d(A)$ is well-defined and finite for each Borel set $A \in \mathcal{B}^q$ and $d \in \mathbb{N}$.

Now, let B be an unbounded Borel set, then it is always possible to decompose it into a sequence $\{B_m\}_{m\in\mathbb{N}}$ with B_m bounded, $\bigcup_{m\in\mathbb{N}} B_m = B$ and $B_m \cap B_M = \emptyset$ for all $m, M \in \mathbb{N}$ with $m \neq M$. From the definition of convergence in probability and the proof for the case of bounded B given above, we get

$$\lim_{d\to\infty} \mathsf{P}_{\boldsymbol{\eta}_0}(|\Psi_d(B_m) - \tilde{\Phi}_q(B_m)| < \varepsilon) = 1, \quad \text{for all } m \in \mathbb{N}, \tag{W45}$$

for any $\varepsilon > 0$. Especially, (W45) also holds for $\varepsilon = \frac{6\varepsilon'}{\pi^2 m^2}$ with $\varepsilon' > 0$. Since the probability of each of these events is tending to one, this is also true for the (countable) intersection of these events. Thus, we get

$$\begin{aligned} |\Psi_d(B) - \tilde{\Phi}_q(B)| = \left| \sum_{m\in\mathbb{N}} \Big(\Psi_d(B_m) - \tilde{\Phi}_q(B_m)\Big) \right| &\leq \sum_{m\in\mathbb{N}} \left| \Psi_d(B_m) - \tilde{\Phi}_q(B_m) \right| \\ &< \sum_{m\in\mathbb{N}} \frac{6\varepsilon'}{\pi^2 m^2} = \varepsilon', \end{aligned}$$

with probability tending to one for $d \to \infty$ for any $\varepsilon' > 0$ and this completes the proof of (26).

For the convergence in P, let $B \in \mathcal{B}^q$ be chosen arbitrarily. We define

$$H_{d,\varepsilon}(\boldsymbol{\eta}') := \mathsf{P}\left(\left| \Psi_d(B) - \tilde{\Phi}_q(B) \right| > \varepsilon \,\middle|\, \boldsymbol{\eta}_0 = \boldsymbol{\eta}' \right)$$

for all $d \in \mathbb{N}$, $\varepsilon > 0$ and $\boldsymbol{\eta}' \in \Theta$, where ϕ_q is the pdf of $\mathcal{N}_q(\mathbf{0}, \mathrm{I}_q)$. Notice that $0 \leq H_{d,\varepsilon} \leq 1$ is true for all $d \in \mathbb{N}$ and $\varepsilon > 0$. Hence, for each $\varepsilon > 0$, $\boldsymbol{\eta} \mapsto 1$ dominates the sequence $\{H_{d,\varepsilon}\}_{d\in\mathbb{N}}$, while $\boldsymbol{\eta} \mapsto 1$ is always $\mathcal{G}$-integrable for a proper $\mathcal{G}$. An application of Lebesgue's theorem of dominated convergence to $\{H_{d,\varepsilon}\}_{d\in\mathbb{N}}$ results in

$$\lim_{d\to\infty} \mathsf{P}\left(\left| \Psi_d(B) - \tilde{\Phi}_q(B) \right| > \varepsilon \right) = \lim_{d\to\infty} \int_\Theta H_{d,\varepsilon} \,\mathrm{d}\mathcal{G} = \int_\Theta \lim_{d\to\infty} H_{d,\varepsilon} \,\mathrm{d}\mathcal{G} = 0, \tag{W46}$$

since $\lim_{d\to\infty} H_{d,\varepsilon}(\boldsymbol{\eta}_0, \varepsilon) = 0$ for all $\varepsilon > 0$ and $\boldsymbol{\eta}_0 \in \mathbb{R}^q$, due to (26), and this finishes the proof.

Proof of Theorem 6. We first prove part (i). Similar to (W42), we utilize the reformulation

$$\mathcal{H}(B|\,\mathbf{Y}^{(d)}) = \frac{\int_B P^{(d)}(\mathbf{Y}^{(d)} \mid \boldsymbol{\eta})\mathfrak{h}(\boldsymbol{\eta})\,\mathrm{d}\boldsymbol{\eta}}{P^{(d)}(\mathbf{Y}^{(d)} \mid \hat{\boldsymbol{\eta}}_d)\det\big(\widehat{\boldsymbol{\Sigma}}_d^{1/2}\big)}\left(\frac{P(\mathbf{Y}^{(d)})}{P^{(d)}(\mathbf{Y}^{(d)} \mid \hat{\boldsymbol{\eta}}_d)\det\big(\widehat{\boldsymbol{\Sigma}}_d^{1/2}\big)}\right)^{-1} \tag{W47}$$

for an arbitrary $B \in \mathcal{B}^q$ with $\boldsymbol{\eta}_0 \notin \partial B$ and each $d \in \mathbb{N}$. If $\boldsymbol{\eta}_0 \in B$, then condition (A7) of Lemma 3 (2.) is satisfied with $G_d(B) := B$ for all $d \in \mathbb{N}$. However, if $\boldsymbol{\eta}_0 \notin B$, then the condition of Lemma 3 (1.) is satisfied with $f := \mathbb{1}_B$. We therefore get

$$\frac{\int_B P^{(d)}(\mathbf{Y}^{(d)} \mid \boldsymbol{\eta})\mathfrak{h}(\boldsymbol{\eta})\,\mathrm{d}\boldsymbol{\eta}}{P^{(d)}(\mathbf{Y}^{(d)} \mid \hat{\boldsymbol{\eta}}_d)\det\big(\widehat{\boldsymbol{\Sigma}}_d^{1/2}\big)} \xrightarrow{\mathsf{P}_{\eta_0}} \mathfrak{h}(\boldsymbol{\eta}_0)(2\pi)^{q/2}\mathbb{1}_B(\boldsymbol{\eta}_0), \tag{W48}$$

for $d \to \infty$. The claim follows from a combination of (W47), (W48) and Corollary 1.

Next, we prove part (ii). In a first step, the existence of $\mathsf{E}(f(\boldsymbol{\eta}) \mid \mathbf{Y}^{(d)})$ for all functions $f\colon \Theta \to \mathbb{R}$, which are continuous and for which the integral $\int_\Theta f(\boldsymbol{\eta})\mathfrak{h}(\boldsymbol{\eta})\,\mathrm{d}\boldsymbol{\eta}$ exists, will be proved. In a second step its consistency for $f(\boldsymbol{\eta}_0)$ will be discussed.

We get

$$\int_\Theta |f(\boldsymbol{\eta})|\mathcal{H}(\mathrm{d}\boldsymbol{\eta} \mid \mathbf{y}^{(d)}) = \frac{\int_\Theta |f(\boldsymbol{\eta})|P^{(d)}(\mathbf{y}^{(d)} \mid \boldsymbol{\eta})\mathfrak{h}(\boldsymbol{\eta})\,\mathrm{d}\boldsymbol{\eta}}{P^{(d)}(\mathbf{y}^{(d)})} \le \frac{\int_\Theta |f(\boldsymbol{\eta})|\mathfrak{h}(\boldsymbol{\eta})\,\mathrm{d}\boldsymbol{\eta}}{P^{(d)}(\mathbf{y}^{(d)})} < \infty,$$

for each $d \in \mathbb{N}$ and for all $\mathbf{y}^{(d)} \in \{0,1\}^d$, because $P^{(d)}(\mathbf{y}^{(d)} \mid \boldsymbol{\eta}) \in (0,1)$, $P^{(d)}(\mathbf{y}^{(d)})$ is positive and independent of $\boldsymbol{\eta} \in \Theta$, and $\int_\Theta |f(\boldsymbol{\eta})|\mathfrak{h}(\boldsymbol{\eta})\,\mathrm{d}\boldsymbol{\eta}$ exists if and only if $\int_\Theta f(\boldsymbol{\eta})\mathfrak{h}(\boldsymbol{\eta})\,\mathrm{d}\boldsymbol{\eta}$ exists. Hence, $\mathsf{E}(f(\boldsymbol{\eta}) \mid \mathbf{Y}^{(d)})$ exists. Furthermore, it remains integrable for $d \to \infty$, as shown next. Notice that the last statement does not follow directly, because $P^{(d)}(\mathbf{y}^{(d)}) \to 0$ for any sequence $\{y_i\}_{i\in\mathbb{N}}$ and $d \to \infty$.

Similar to (W42), we start with the representation

$$\begin{aligned}&\int_\Theta |f(\boldsymbol{\eta})|\,\mathcal{H}(\mathrm{d}\boldsymbol{\eta} \mid \mathbf{Y}^{(d)}) \\ &\quad= \frac{\int_\Theta |f(\boldsymbol{\eta})|P^{(d)}(\mathbf{Y}^{(d)} \mid \boldsymbol{\eta})\mathfrak{h}(\boldsymbol{\eta})\,\mathrm{d}\boldsymbol{\eta}}{P^{(d)}(\mathbf{Y}^{(d)} \mid \hat{\boldsymbol{\eta}}_d)\det(\widehat{\boldsymbol{\Sigma}}_d)^{1/2}}\left(\frac{P^{(d)}(\mathbf{Y}^{(d)})}{P^{(d)}(\mathbf{Y}^{(d)} \mid \hat{\boldsymbol{\eta}}_d)\det\big(\widehat{\boldsymbol{\Sigma}}_d\big)^{1/2}}\right)^{-1},\end{aligned} \tag{W49}$$

for each $d \in \mathbb{N}$. We decompose for an arbitrary $\delta > 0$ as follows

$$\begin{aligned}\int_\Theta |f(\boldsymbol{\eta})|P^{(d)}(\mathbf{Y}^{(d)} \mid \boldsymbol{\eta})\mathfrak{h}(\boldsymbol{\eta})\,\mathrm{d}\boldsymbol{\eta} &= \int_{B_\delta(\boldsymbol{\eta}_0)} |f(\boldsymbol{\eta})|P^{(d)}(\mathbf{Y}^{(d)} \mid \boldsymbol{\eta})\mathfrak{h}(\boldsymbol{\eta})\,\mathrm{d}\boldsymbol{\eta} \\ &\quad+ \int_{\Theta\setminus B_\delta(\boldsymbol{\eta}_0)} |f(\boldsymbol{\eta})|P^{(d)}(\mathbf{Y}^{(d)} \mid \boldsymbol{\eta})\mathfrak{h}(\boldsymbol{\eta})\,\mathrm{d}\boldsymbol{\eta}.\end{aligned}$$

Due to the integrability of f with respect to $\mathcal{H}$, Lemma 3 (1.) implies

$$\frac{\int_{\Theta\setminus B_\delta(\boldsymbol{\eta}_0)} |f(\boldsymbol{\eta})| P^{(d)}(\mathbf{Y}^{(d)} \mid \boldsymbol{\eta})\mathfrak{h}(\boldsymbol{\eta})\,\mathrm{d}\boldsymbol{\eta}}{P^{(d)}(\mathbf{Y}^{(d)} \mid \hat{\boldsymbol{\eta}}_d)\det(\hat{\boldsymbol{\Sigma}})^{1/2}} \xrightarrow{\mathsf{P}_{\boldsymbol{\eta}_0}} 0, \qquad d\to\infty. \tag{W50}$$

Since f is continuous, we get

$$\sup_{\boldsymbol{\eta}\in B_\delta(\boldsymbol{\eta}_0)} |f(\boldsymbol{\eta})| =: C_1 < \infty. \tag{W51}$$

Hence,

$$\int_{B_\delta(\boldsymbol{\eta}_0)} |f(\boldsymbol{\eta})| P^{(d)}(\mathbf{Y}^{(d)} \mid \boldsymbol{\eta})\mathfrak{h}(\boldsymbol{\eta})\,\mathrm{d}\boldsymbol{\eta} \le C_1 \int_{B_\delta(\boldsymbol{\eta}_0)} P^{(d)}(\mathbf{Y}^{(d)} \mid \boldsymbol{\eta})\mathfrak{h}(\boldsymbol{\eta})\,\mathrm{d}\boldsymbol{\eta}. \tag{W52}$$

An application of Lemma 3 (2.) on the right-hand side results in

$$C_1\frac{\int_{B_\delta(\boldsymbol{\eta}_0)} P^{(d)}(\mathbf{Y}^{(d)} \mid \boldsymbol{\eta})\mathfrak{h}(\boldsymbol{\eta})\,\mathrm{d}\boldsymbol{\eta}}{P^{(d)}(\mathbf{Y}^{(d)} \mid \hat{\boldsymbol{\eta}}_d)\det\left(\widehat{\boldsymbol{\Sigma}}_d\right)^{1/2}} \xrightarrow{\mathsf{P}_{\boldsymbol{\eta}_0}} C_1\mathfrak{h}(\boldsymbol{\eta}_0)(2\pi)^{q/2}, \tag{W53}$$

because $G_d(B) \coloneqq B_\delta(\boldsymbol{\eta}_0)$, $d\in\mathbb{N}$, satisfies (A7). By summing up (W49)–(W53) and applying Corollary 1 to the second factor on the right-hand side of (W49) we obtain

$$\lim_{d\to\infty} \mathsf{P}_{\boldsymbol{\eta}_0}\left(\int_\Theta |f(\boldsymbol{\eta})|\,\mathcal{H}(\mathrm{d}\boldsymbol{\eta} \mid \mathbf{Y}^{(d)}) < C_1\right) = 1.$$

Next, we compute the value of $\mathsf{E}(f\circ\boldsymbol{\eta} \mid \mathbf{Y}^{(d)})$ for $d\to\infty$. Since $\mathcal{H}(\,\cdot \mid \mathbf{Y}^{(d)})$ is always a probability measure, for every $\delta>0$ it holds

$$\begin{aligned}
&\left|\int_\Theta f(\boldsymbol{\eta})\,\mathcal{H}(\mathrm{d}\boldsymbol{\eta} \mid \mathbf{Y}^{(d)}) - f(\boldsymbol{\eta}_0)\right| = \left|\int_\Theta f(\boldsymbol{\eta})\,\mathcal{H}(\mathrm{d}\boldsymbol{\eta} \mid \mathbf{Y}^{(d)}) - f(\boldsymbol{\eta}_0)\mathcal{H}(\,\Theta \mid \mathbf{Y}^{(d)})\right| \\
&= \left|\int_{B_\delta(\boldsymbol{\eta}_0)} \big(f(\boldsymbol{\eta}) - f(\boldsymbol{\eta}_0)\big)\,\mathcal{H}(\mathrm{d}\boldsymbol{\eta} \mid \mathbf{Y}^{(d)})\right. \\
&\quad \left. + \int_{\Theta\setminus B_\delta(\boldsymbol{\eta}_0)} f(\boldsymbol{\eta})\,\mathcal{H}(\mathrm{d}\boldsymbol{\eta} \mid \mathbf{Y}^{(d)}) - f(\boldsymbol{\eta}_0)\mathcal{H}(\,\Theta\setminus B_\delta(\boldsymbol{\eta}_0) \mid \mathbf{Y}^{(d)})\right| \\
&\le \left|\int_{B_\delta(\boldsymbol{\eta}_0)} \big(f(\boldsymbol{\eta}) - f(\boldsymbol{\eta}_0)\big)\,\mathcal{H}(\mathrm{d}\boldsymbol{\eta} \mid \mathbf{Y}^{(d)})\right| + \left|\int_{\Theta\setminus B_\delta(\boldsymbol{\eta}_0)} f(\boldsymbol{\eta})\,\mathcal{H}(\mathrm{d}\boldsymbol{\eta} \mid \mathbf{Y}^{(d)})\right| \\
&\quad + |f(\boldsymbol{\eta}_0)|\cdot\mathcal{H}(\,\Theta\setminus B_\delta(\boldsymbol{\eta}_0) \mid \mathbf{Y}^{(d)}).
\end{aligned}$$

Using a similar representation to (W49) and applying (W50) and Corollary 1 to it, leads to

$$\left|\int_{\Theta\setminus B_\delta(\boldsymbol{\eta}_0)} f(\boldsymbol{\eta})\,\mathcal{H}(\mathrm{d}\boldsymbol{\eta} \mid \mathbf{Y}^{(d)})\right| \xrightarrow{\mathsf{P}_{\boldsymbol{\eta}_0}} 0 \quad \text{for } d\to\infty.$$

Further, part (i) implies

$$|f(\boldsymbol{\eta}_0)| \cdot \mathcal{H}(\,\Theta \setminus B_\delta(\boldsymbol{\eta}_0) \mid \mathbf{Y}^{(d)}) \xrightarrow{\mathsf{P}_{\eta_0}} 0 \quad \text{for } d \to \infty.$$

Finally, since f is continuous at $\boldsymbol{\eta}_0$, for each $\varepsilon > 0$ there is a $\delta' > 0$ such that $|f(\boldsymbol{\eta}) - f(\boldsymbol{\eta}_0)| < \varepsilon$, for all $\boldsymbol{\eta} \in B_{\delta'}(\boldsymbol{\eta})$. Therefore, for every $\varepsilon > 0$ we get

$$\begin{aligned}\left| \int_{B_{\delta'}(\boldsymbol{\eta}_0)} \big(f(\boldsymbol{\eta}) - f(\boldsymbol{\eta}_0)\big)\, \mathcal{H}(\mathrm{d}\boldsymbol{\eta} \mid \mathbf{Y}^{(d)}) \right| &\le \int_{B_{\delta'}(\boldsymbol{\eta}_0)} \big|f(\boldsymbol{\eta}) - f(\boldsymbol{\eta}_0)\big|\, \mathcal{H}(\mathrm{d}\boldsymbol{\eta} \mid \mathbf{Y}^{(d)}) \\ &\le \varepsilon \cdot \mathcal{H}(B_{\delta'}(\boldsymbol{\eta}_0) \mid \mathbf{Y}^{(d)}) \le \varepsilon.\end{aligned}$$

Since $\delta > 0$ was chosen arbitrarily in the decomposition of Θ, we get for each $\varepsilon > 0$

$$\begin{aligned}\lim_{d\to\infty} \mathsf{P}_{\boldsymbol{\eta}_0}\Big(\big|\mathsf{E}(f \circ \boldsymbol{\eta} \mid \mathbf{Y}^{(d)}) - f(\boldsymbol{\eta}_0)\big| > \varepsilon\Big) &= 1 - \lim_{d\to\infty} \mathsf{P}_{\boldsymbol{\eta}_0}\Big(\big|\mathsf{E}(f \circ \boldsymbol{\eta} \mid \mathbf{Y}^{(d)}) - f(\boldsymbol{\eta}_0)\big| \le \varepsilon\Big) \\ &= 0,\end{aligned}$$

which is what we had to show for the consistency of $\mathsf{E}(f(\boldsymbol{\eta}) \mid \mathbf{Y}^{(d)})$. The consistency of $\mathsf{E}(\boldsymbol{\eta} \mid \mathbf{Y}^{(d)})$ follows directly by considering the mappings $\boldsymbol{\eta} \mapsto \eta_j$, $j \in \{1, \ldots, q\}$, in the first part, which are continuous and by assumption integrable.

References

Chang, H.-H. and Stout, W. (1991). The asymptotic posterior normality of the latent trait in an IRT model. Technical Report ONR Research Report 91-4, Department of Statistics, University of Illinois at Urbana-Champaign.

Kornely, M. J. K. (2021). *Multidimensional Modeling and Inference of Dichotomous Item Response Data.* PhD thesis, RWTH Aachen University, Germany.

Lehmann, E. L. and Casella, G. (1998). *Theory of Point Estimation.* Springer, New York, NY, second edition.

Serfling, R. J. (1980). *Approximation Theorems of Mathematical Statistics.* Probability and Statistics. John Wiley & Sons, New York, NY.

Witting, H. and Müller-Funk, U. (1995). *Mathematische Statistik II.* Teubner, Stuttgart.
